# Machine learning-based prediction of soiling losses in photovoltaic modules under different cleaning frequencies: an experimental investigation

**DOI:** 10.1038/s41598-026-45485-2

**Published:** 2026-04-14

**Authors:** Ashutosh Shukla, Rupendra Kumar Pachauri, Ranjan Walia, Vinay Gupta

**Affiliations:** 1 Electrical Cluster, School of Advanced Engineering, UPES, Dehradun, 248007 India; 2 Miyan Research Institute, International University of Business, Agriculture and Technology, Dhaka, 1230 Bangladesh; 3 UCRD & CSE-APEX, Chandigarh University, Mohali, Punjab India; 4 Department of Electrical Engineering, Manipal University Jaipur, Jaipur, India

**Keywords:** Soiling loss, Cleaning frequency, Stacking model, Performance prediction, Solar photovoltaic system, Power loss, Energy science and technology, Engineering, Environmental sciences

## Abstract

Accumulation of dust on solar panels lowers performance and limits energy production, particularly in dry locations. Dust accumulation on photovoltaic panels diminishes performance and reduces energy output, especially in arid regions. This study uses four identical modules in Roorkee, India, from October to December to examine the impact of cleaning frequency on photovoltaic (PV) performance. The reference panel is cleaned daily, while the remaining panels are cleaned weekly, biweekly, and monthly. Alongside short-circuit current measurements, environmental parameters including global horizontal irradiance, ambient temperature, wind speed, and relative humidity are continuously recorded. In this study, soiling loss (%) is examined as the primary performance indicator under various cleaning intervals to observe dust accumulation progression and its impact on the performance of the solar photovoltaic module. Experimental data are utilized to develop an empirical regression model that describes the trend of dust accumulation. The daily average soiling loss ranges between 0.17 and 0.21%. Furthermore, machine learning models, including Decision Tree, K-Nearest Neighbour, support vector regression, artificial neural network, and a stacking ensemble method, are developed for accurate prediction of soiling loss from environmental variables and cleaning frequency. The stacking model consistently achieves the best performance across all months, with root mean square error as low as 0.03–0.045, mean absolute error below 0.03, and R² = 0.999 compared to other models. Moreover, statistical analyses such as Bland–Altman plots and the Wilcoxon signed-rank test are employed to validate the significance and agreement of the predicted outcomes. The study highlights the benefits of data-driven solutions for predictive operation and maintenance of solar photovoltaic systems and provides valuable insights into the impact of cleaning frequency on reducing soiling losses.

## Introduction

**Fig. 1:**
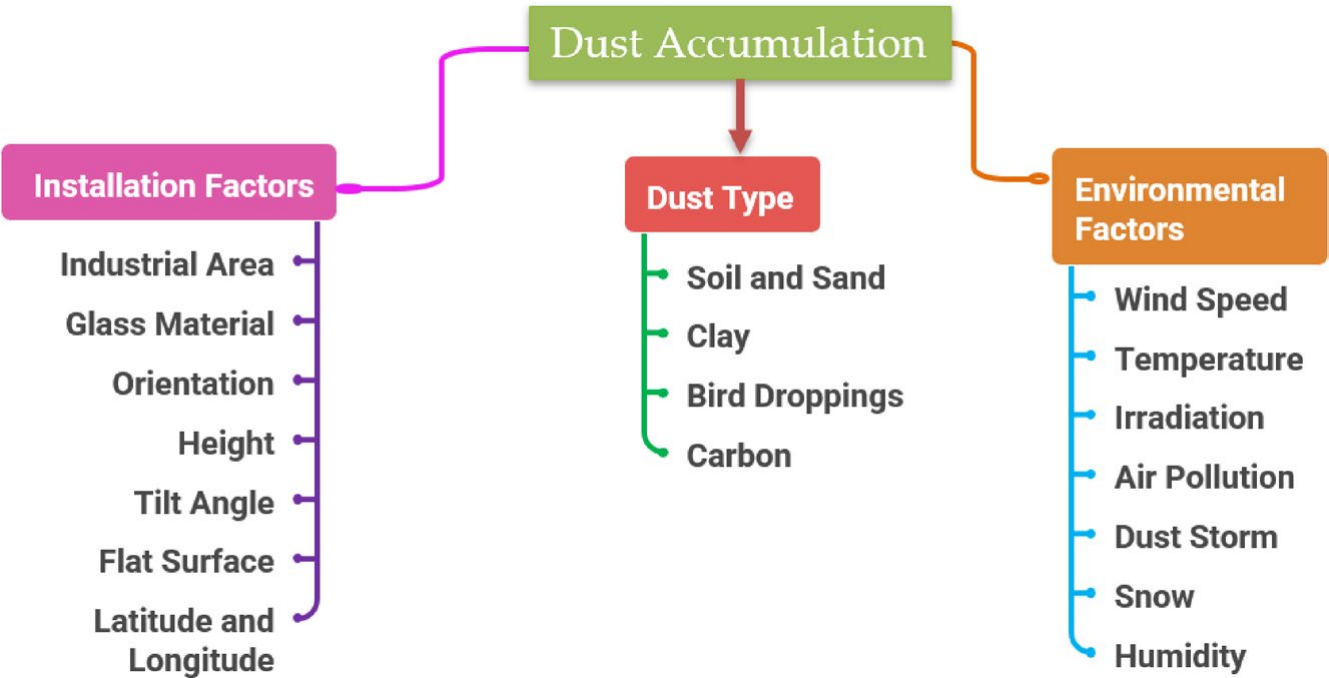
Factors contributing dust accumulation impacts on PV modules.

The solar energy extensively uses for heating purpose, desalination of water, cooling process and production of electrical energy, serving a diverse array of applications from home to industrial as well in agricultural sectors^1,2^. About half of the world’s electricity will come from wind and solar power alone by 2050^3^. Until then, around two-thirds of India’s electricity will come from solar and wind. The price of solar energy has dropped by around 85% since 2010^4^. The primary factors for India’s solar PV to develop exponentially are the sharp decline in solar energy prices and Indian government policies subsidies schemes to use solar PV technology to produce power. India has abundant solar insolation due its location inside the tropical belt, enabling it to fulfil its daily electrical requirements. It gets an average solar insolation of 4 to 7 kWh/m² and around 2300 to 3200 h of sunlight annually^5^. Numerous components affect the efficiency of solar photovoltaic power generating systems as shown in Fig. 1 such as type of material, spacing of solar cell, module area, tilt angle and orientation, environmental condition, surface dust of solar photovoltaic (PV) panels. The most often occurring element influencing the solar photovoltaic panel performance is surface dust^6–8^. The accumulation of dust on the surface of solar panels can result in changes in the electrical charecteristics of the panel array. These changes can cause the panels to have a reverse bias, which in turn can result in a loss of power generated by the panels^9^. The synthesized bio-derived TiO₂ nanoparticles using plant extract and demonstrated improved photovoltaic performance through enhanced light absorption and charge transport, highlighting the importance of material-level enhancements for solar cell efficiency^10^.The soiling rates vary between 0.05% and 0.55% per day in India^11,12^, while in Dhaka, Bangladesh, it is 0.78%^13^.

Large-scale solar farms in remote locations are particularly affected by the soiling issue. This is due to the fact that regular cleaning and inspection may be challenging and costly, given the expenses of labor and long-distance travel^14,15^. In order to determine the amount of power that is lost by dirty photovoltaic modules, it is desirable to have automated soiling detection. Inadequate maintenance of the cleanliness of solar photovoltaic panel surfaces will lead to significant economic losses. Consequently, utilising precise and effective techniques to identify dust buildup on the surfaces of solar photovoltaic panels is crucial. This facilitates prompt cleaning of the panels, thereby ensuring their safe and efficient functioning. Dust and ambient temperature energy losses were quantified using artificial neural network (ANN) and extreme learning machine (ELM) algorithms^16^. Both the ELM and ANN models predict 91.42 and 90.69% accurately. Multivariate Linear Regression (MLR) and ANN models were used to estimate dust-related energy and economic losses in solar panels^17^. ANN and MLR models estimated dust-related cost and energy losses at 89.97% and 86.78%, respectively. In another study Adaptive Neuro-Fuzzy Inference System (ANFIS) was used to predict the dust-exposed solar module performance^18^. The ANFIS model achieves root mean square error (RMSE) of 0.18719 and coefficient of determination (R²) of 0.99803 for monocrystalline silicon PV modules. In comparison, polycrystalline PV modules have an RMSE of 0.87098 and a R² of 0.99714. The study in^19^ predicted dust-induced PV panel performance deterioration in Qatar using ANN and MLR models. The ANN model has a R² of 0.537 and mean square error (MSE) of 0.0038, while the MLR model has a R² of 0.167 and an MSE of 0.0082. The ANN model outperformed the MLR model. Study in^20^ estimated dust losses using artificial neural networks. Modern technology allows ANN to estimate losses with normalized root mean square errors (NRMSE) of 6.79 and correlation coefficients (R) of 0.91. Another of^21^ utilised AMM, MLR, Interactive Multivariate Linear Regression Model (MLRWI) and Response Surface Methodology (RSM), to predict the loss caused by dust on solar PV module surface. The artificial neural network produced better predictive results than the other machine learning models. The results for R² and RMSE are 0.813 and 0.026, respectively. The separate studied carried out focusing on machine learning (ML) methods implemented and their performance as shown in Table 1.

**Table 1:** Taxonomy on machine learning models.

Ref.	Methods used	Best method	Input features	Accuracy (%)
^22^	LSTM KNN Hybrid LSTM + KNN model	Hybrid	Sunshine hour Humidity Temperature Solar radiation.	98.22
^23^	LSTM BPNN	LSTM	Season Humidity Temperature Solar radiation	96.30
^24^	Linear regression random forest Decision tree Multilayer perceptron, Long short-term memory	MLP	Sunshine hour Humidity Temperature Solar radiation.	98.03
^25^	SVR	SVR	PM 10 /2.5 Humidity Temperature Solar radiation.	91.02
^26^	ANN	ANN	Wind speed Humidity Temperature Solar radiation.	87.04
^27^	SARIMAX model Ensemble voting.	SARIMAX	Wind speed Humidity Cleaning frequency Solar radiation.	92.12
^28^	Linear Regressor Random forest XGBoost regression, KNN Regressor AdaBoost Regressor	XGBoost	Wind speed Temperature Solar radiation. Atmospheric pressure	97.88
^29^	LSTM GRU AutoEncoder LSTM Auto-GRU.	Auto-LSTM	Ambient temperature PV module temperature Wind speed Wind direction Solar radiation	98.90
^30^	ANN LSTM CNN RNU	RNN	Ambient temperature PV module temperature Wind speed Wind direction Solar radiation Sunshine hour	97.85
^31^	RF SVM LR DT NN	RF	Ambient temperature Wind speed DNI GHI DHI	98.64
^32^	CNN LSTM MLP Random forest	MLP	Wind speed Ambient temperature Solar radiation. Pressure Cloud cover	98.02
^33^	CNNs	CNNs	RGB images of solar panels	83.37
^34^	LR RF DT MLP LSTM	MLP	Ambient temperature PV module temperature Wind speed Wind direction Solar radiation	98.72
^35^	CNN SVM RF KNN naïve-Bayes Decision tree	RF	Long-term irradiance variability PVsyst model uncertainty Satellite irradiance modelling	93.50

**Fig. 2:**
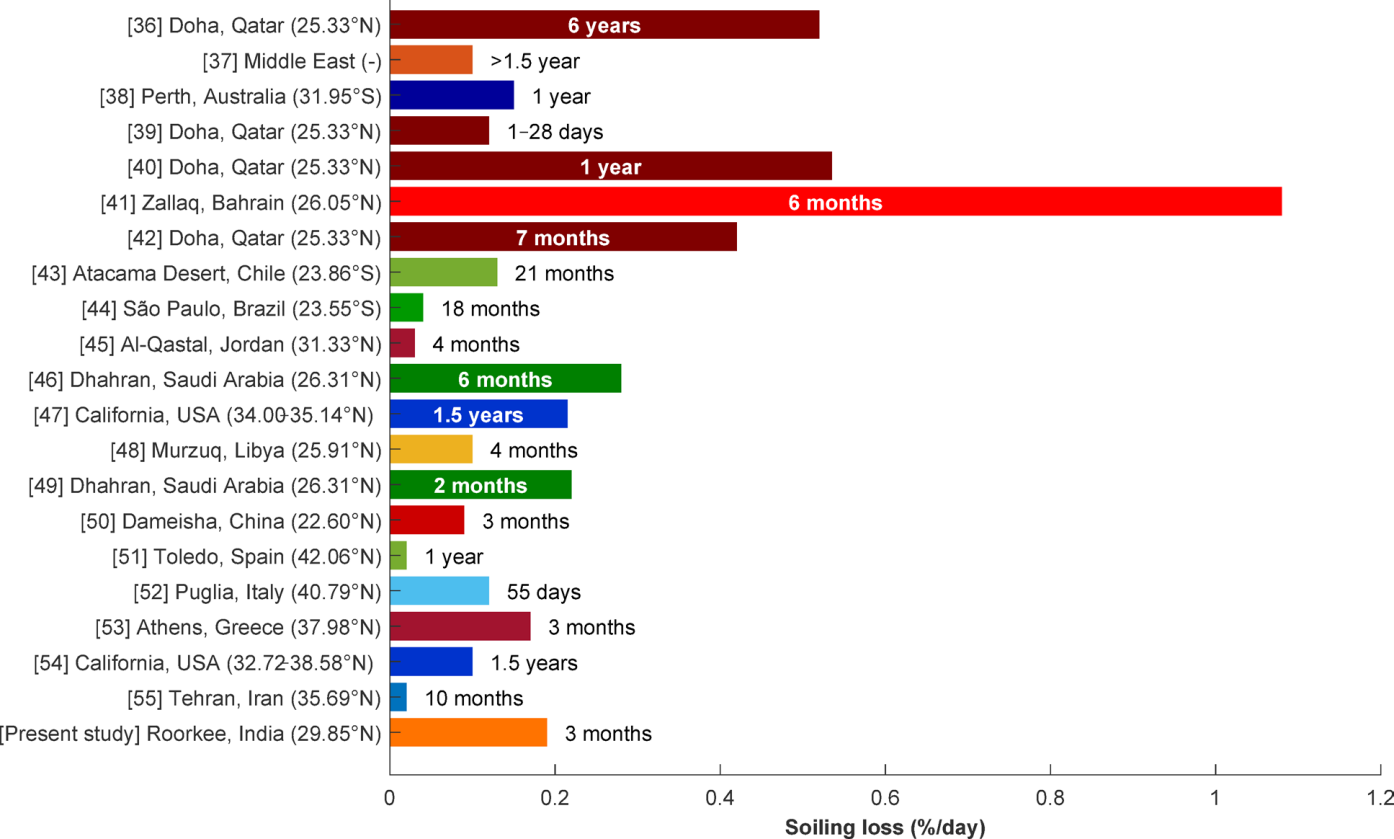
Soiling loss and study duration across locations.

The studies summarised in Fig. 2 show that the reported soiling losses vary widely with location, exposure duration and local climatic conditions —observed rates in the literature span roughly 0.1% to 1.1% perday, with the highest values typically found in arid, dusty environments (e.g. Bahrain, Qatar) and lower values in regions with occasional rainfall or wind cleaning. Differences in measurement period, panel tilt, dust type, and cleaning practice (and the diversity of experimental protocols) make direct comparison difficult. Overall, the literature indicates a clear need for (a) longer-term, standardized measurements across diverse climates, (b) studies that relate soiling loss to measured environmental drivers (global horizontal irradiance (GHI), wind speed (WS), relative humidity (RH), dust deposition rate (DDR)), and (c) predictive models ( ML approaches) validated against controlled experiments^36–55^. These gaps motivate the present work, which combines experimental measurements with ML models to produce robust soiling-loss predictions.

### Novelty of work

- Real-world natural dust build up on PV panels under four cleaning frequencies (daily, weekly, biweekly, monthly) is studied instead of controlled dust deposition research, offering practical insights.
- This work established two empirical models: one for PV short circuit current (I_sc_) prediction and one for soiling loss (SL), encompassing both electrical performance and soiling effects.
- Novel stacking model for SL prediction and comparison with other ML models (ANN, SVM, KNN, DT) provide strong empirical and data-driven comparisons.
- Bland–Altman analysis and the Wilcoxon signed-rank test for model validation provide a unique level of statistical validity, assuring model dependability.

## Methodology

**Fig. 3:**
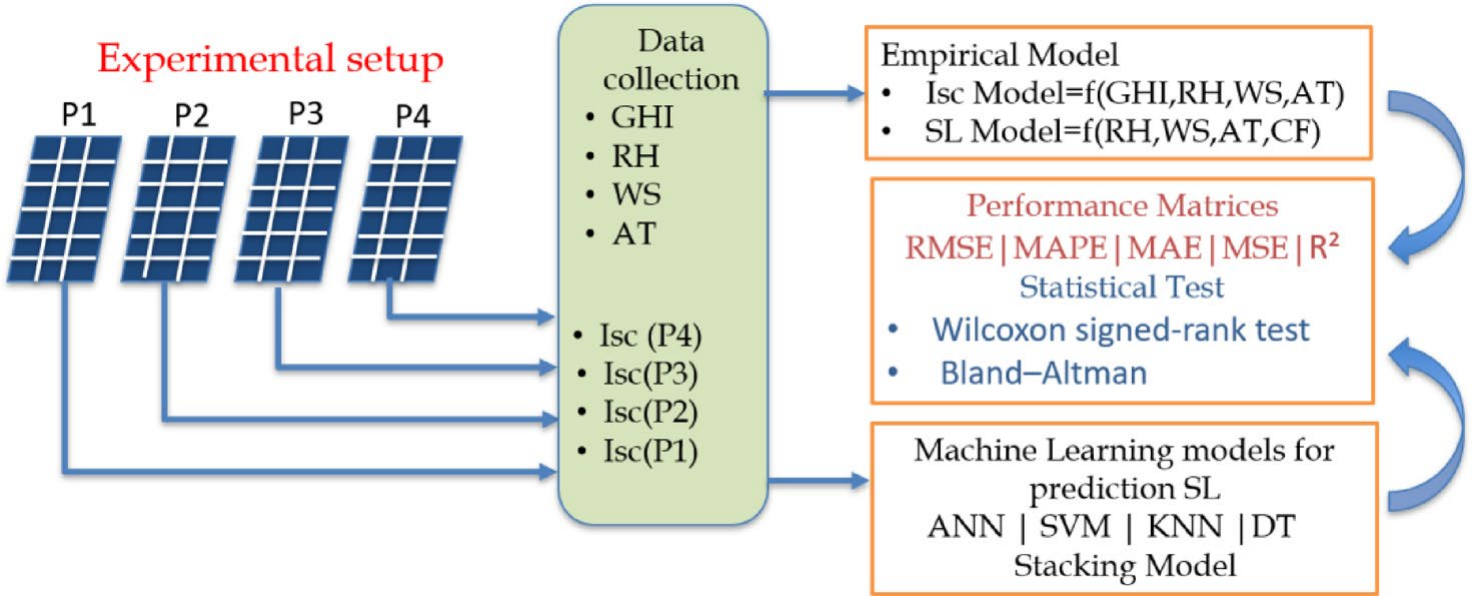
Methodology of the work.

The methodology of the present work is shown in Fig. 3 with an experimental setup consisting of four PV panels subjected to different cleaning frequencies (P1-daily, P2-weekly, P3-biweekly, and P4-monthly). Data including GHI, RH, WS, ambient temperature (AT), and the short-circuit current (Isc) of each panel were recorded using a data logger. Based on the collected data, two empirical models were developed: An Isc model as a function of environmental parameters such as global horizontal irradiance (GHI), ambient temperature (AT), wind speed (WS), relative humidity (RH) are considered in present study, and a soiling loss (SL) model as a function of RH, WS, AT, and cleaning frequency (CF). To improve predictive capability, machine learning models (ANN, SVM, KNN, DT, and stacking ensemble) were implemented for SL prediction. The performance of empirical and ML models was compared using evaluation metrics RMSE, mean absolute percentage error (MAPE), MAE, MSE, and R². The statistical validation using Bland–Altman analysis and the Wilcoxon signed-rank test was carried out to assess the significance and agreement of the models.

### Experimental setup

The experimental work was conducted in Roorkee, India (29.86°N, 77.89°E), located in the Indo-Gangetic plain and characterised by a subtropical climate with distinct winter, summer, and monsoon seasons. The study period, October to December, represents the dry-winter season with frequent dust-laden winds, moderate humidity, and occasional foggy conditions, making it suitable for investigating soiling effects. Rainfall-induced natural cleaning was avoided to maintain experimental consistency and isolate the effects of environmental variables and manual cleaning intervals.

**Fig. 4:**
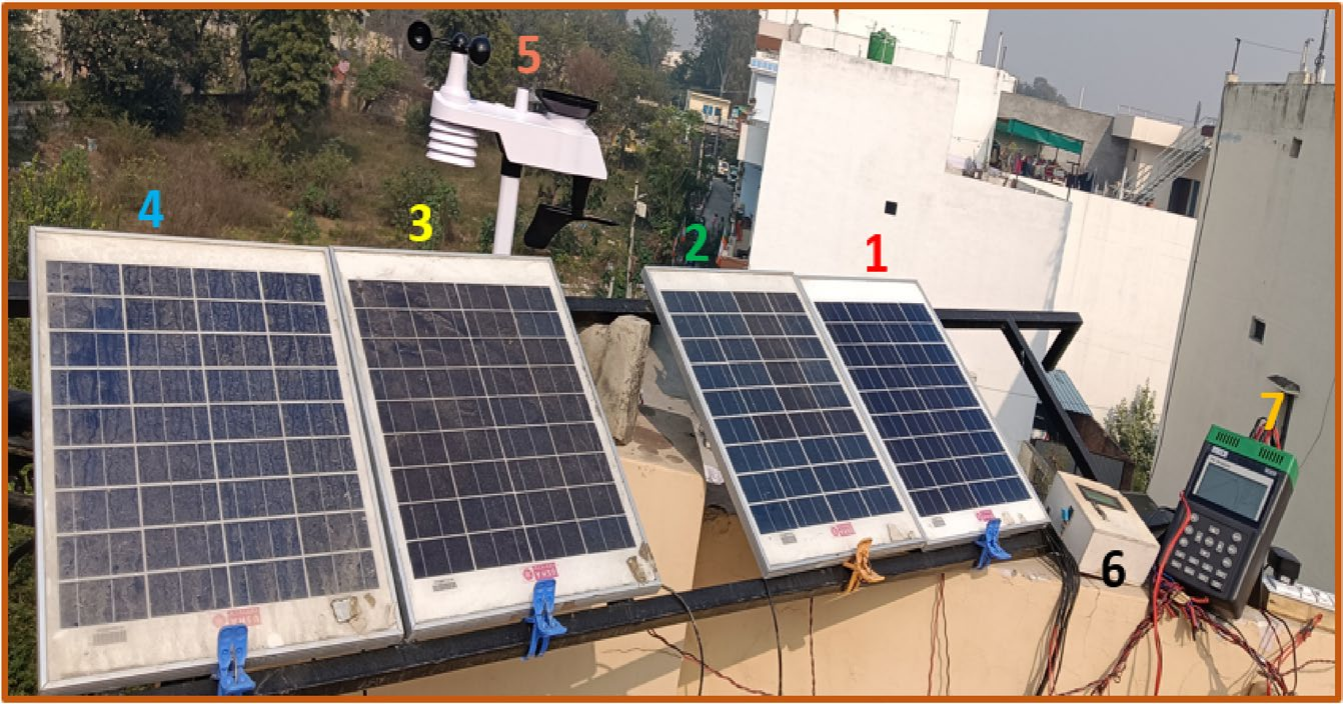
Experimental setup: (1–4) PV modules with different cleaning frequencies, (5) weather station, (6) data logger, and (7) PV analyser.

The experimental setup is shown in Fig. 4, consisted four identical crystalline silicon PV modules, each rated at the same electrical capacity $\:{P}_{max}$ 20 W, installed outdoors with a fixed tilt angle of 30^0^ and south-facing orientation to maximize solar exposure. All the PV modules are mounted on a common frame to ensure that they experienced identical environmental conditions such as GHI, AT, RH, and WS.

The present study isconducted during the dry season because the primary objectiveis to evaluate the impact of manual cleaning at fixed and predefined intervals (daily, weekly, biweekly, monthly) under controlled accumulation conditions. During the monsoon season, frequent rainfall events act as natural cleaning mechanisms. Such stochastic and uncontrolled cleaning would interfere with the predefined manual cleaning schedules and compromise the controlled comparison between different cleaning frequencies.

The data acquisition architecture shown in Fig. 4 as follows:

- The ATMEGA2560-based data logger is shown measuring:
  - Global horizontal irradiance (GHI).
  - Ambient temperature (AT).
  - Relative humidity (RH).
  - PV module current and voltage.
  - Timestamp via real-time clock (RTC).
- The Weather Station is separately indicated as the source of:
  - Wind Speed (WS).

### Instrumentation and measurement procedure

The experiment is conducted under natural outdoor exposure, allowing dust to accumulate under real environmental conditions. Although dust composition may vary geographically, the measured electrical performance and environmental parameters inherently capture the net effect of dust deposition and adhesion. This approach ensures that the dataset reflects realistic soiling behaviour and provides a reproducible foundation for predictive modelling.

To evaluate the impact of cleaning frequency on soiling losses, four panels are exposed to several cleaning regimens. Panel P1 served as the clean reference and underwent daily cleaning, whilst P2, P3, and P4 were cleaned weekly, biweekly, and every four weeks, respectively. Cleaning occurred at 06:00 AM utilizing distilled water and a gentle, lint-free cloth to avert scratches or more surface contaminants. Electrical measurements encompassed the short-circuit current of each module, recorded at consistent intervals as the principal performance metric for dust build-up. Meteorological parameters were continually recorded, including global horizontal irradiance, ambient temperature, relative humidity, and wind speed. The data were obtained using calibrated sensors incorporated with a data logging system, ensuring synchronous environmental and electrical recordings. The experimental configuration included several sensors to enable precise data collection and real-time observation of solar PV system metrics presented in Table 2.

**Table 2:** Component utilized in Experimental work.

Component	Specification	Description
ATMEGA2560 (microcontroller board)	Operating (logic) voltage: 5 V Recommended input (Vin): 7–12 V Digital I/O pins: 54 Analog inputs: 16 channels CPU clock: 16 MHz On-board flash memory: 256 KB	Central DAQ and controller — reads sensors, timestamps and logs data.
Current sensor (ACS712 or equivalent)	Range: 0–20 A	To measures panel/module current for power and loss calculations.
Voltage sensing (resistor divider + protection)	Range: 0–45 V	To monitors PV module voltage safely within MCU ADC input range.
Real-time clock (RTC)	Accuracy ≈ ± 2 ppm Time base: 1 s	Provides reliable timestamps for measurements.
Solar power (irradiance) meter	Range up to ~ 1200 W/m²; accuracy ≈ ± 10 W/m²	To measures incident solar irradiance to normalize PV output.
Ambient sensor (DHT11)	Temperature ~ 0–50 °C; humidity measurement	Records ambient temperature and relative humidity.
PV module	$\:{P}_{MPP}$ =20 W $\:{V}_{oc}$ =22 V $\:{I}_{SC\:}$ =1.3 A $\:{I}_{mpp}=1.2A$	Used for soiling and performance experiments.

**Fig. 5:**
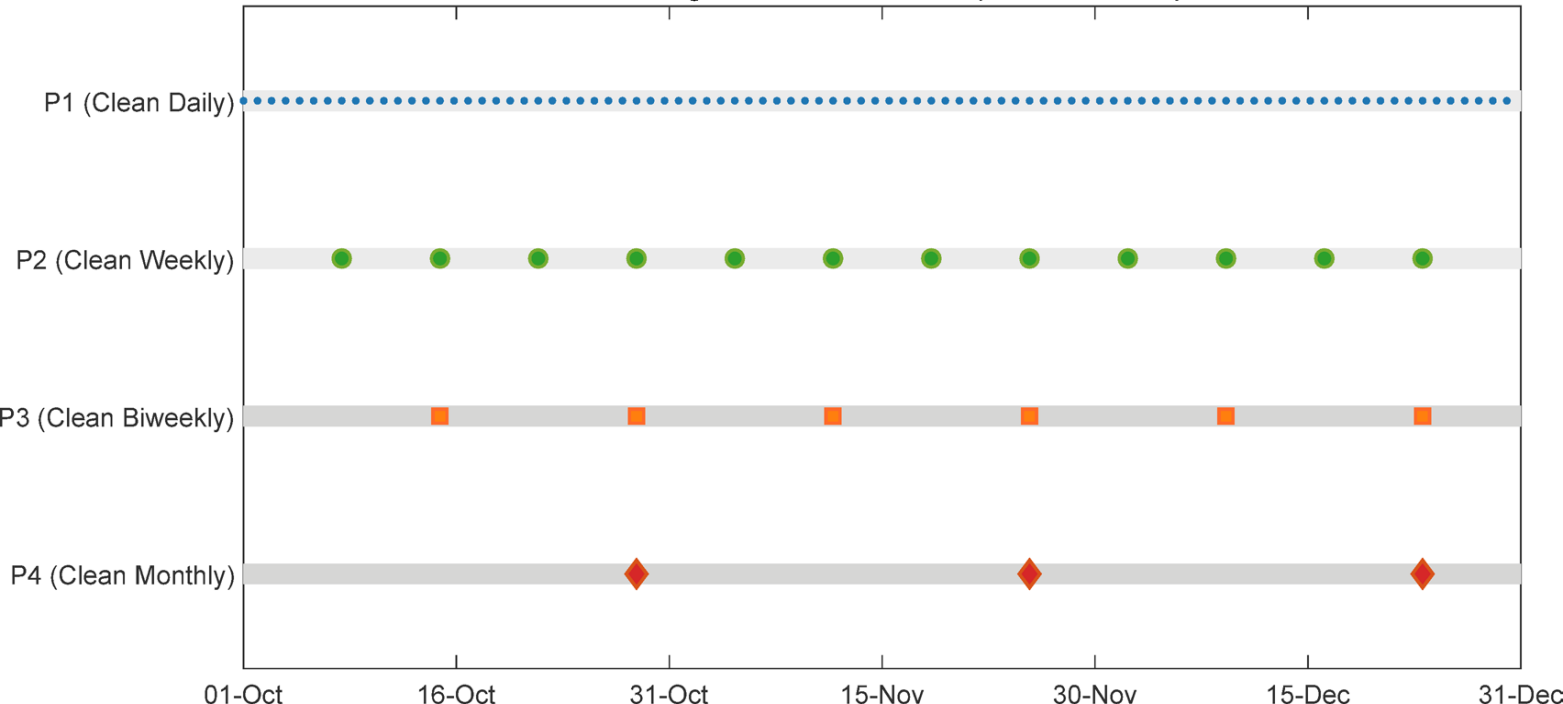
Cleaning schedule timeline (Oct-Dec 2024).

Electrical and environmental parameters (GHI, AT, RH, WS, $\:{I}_{SC}$, and voltage) were recorded at fixed and uniform intervals using the ATMEGA2560-based data acquisition system. Measurements were sampled at regular intervals (hourly), ensuring temporal consistency throughout the experimental period. For modeling analysis, the recorded data were aggregated into daily average values to represent the cumulative effect of dust deposition under each cleaning frequency. The Fig. 5 illustrates the cleaning schedule followed during the experimental period from October to December, clearly highlighting the systematic maintenance intervals adopted for each panel.

**Fig. 6:**
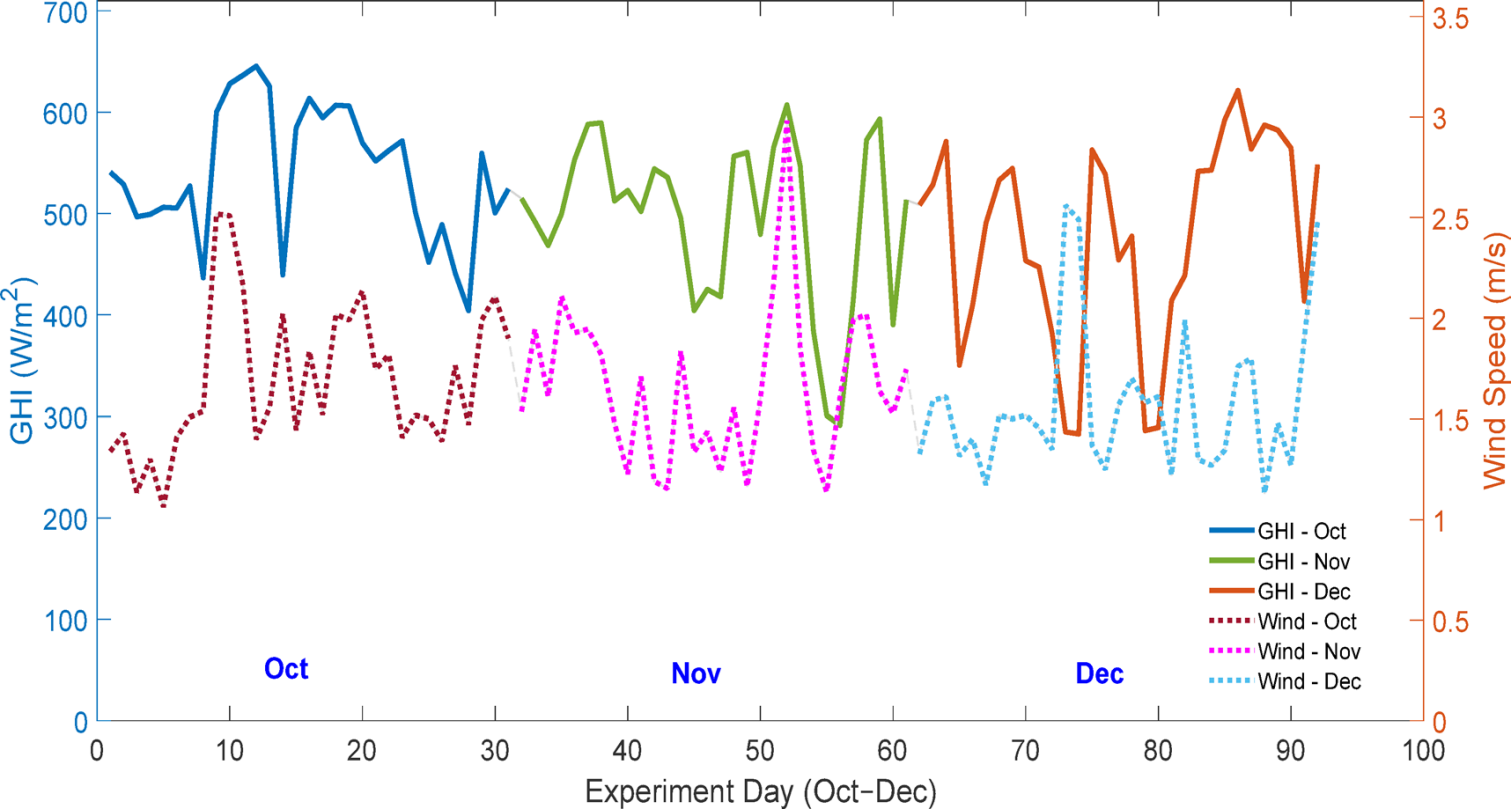
Daily average global horizontal irradiance and wind speed during the experimental period (October–December 2024).

**Fig. 7:**
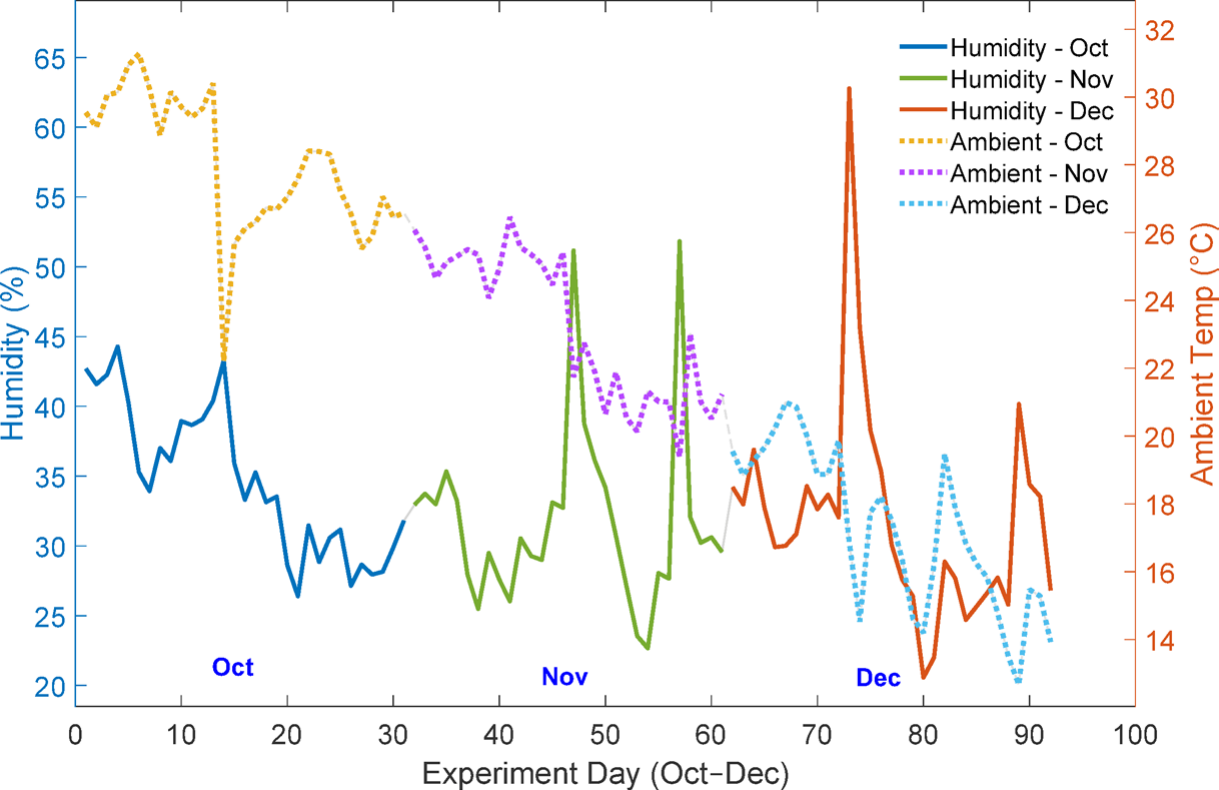
Daily average relative humidity and ambient temperature during the experimental study (October–December 2024).

To account for the influence of external climatic variables, meteorological parameters were continuously recorded during the experimental study. The Fig. 6 illustrates the daily average variation of global horizontal irradiance and wind speed, while Fig. 7 presents the daily average variation of relative humidity and ambient temperature from October to December 2024. Statistical description of the collected data is shown in Table 3. These measurements highlight the dynamic environmental conditions under which the PV panels operated, providing essential context for analysing the soiling effect and validating the empirical and machine learning models.

**Table 3:** Description of the monthly collected data year 2024 | P1-clean daily |P2- clean weekly| P3- clean biweekly | P4- clean monthly.

Month		Ambient temperature (^0^C)	Wind speed (m/s)	Relative humidity (%)	Irradiance (W/m2)	P1 I_SC_ (A)	P2 I_SC_ (A)	P3 I_SC_ (A)	P4 I_SC_ (A)
October	min	22.21	1.06	50.55	184.36	0.23	0.23	0.22	0.21
	max	31.27	2.51	81.63	658.81	0.85	0.84	0.82	0.82
	mean	28.14	1.65	64.42	421.69	0.54	0.54	0.53	0.52
	std	2.09	0.37	7.86	146.63	0.19	0.18	0.18	0.18
November	min	19.38	1.13	49.88	140.54	0.18	0.18	0.17	0.16
	max	26.45	2.98	77.52	674.36	0.87	0.85	0.85	0.8
	mean	23.4	1.66	59.36	432.94	0.56	0.55	0.54	0.53
	std	2.18	0.4	8.88	145.44	0.18	0.18	0.18	0.17
December	min	12.66	1.12	32.63	120	0.15	0.15	0.14	0.14
	max	20.99	2.56	84.3	708.4	0.92	0.9	0.88	0.84
	mean	17.51	1.54	44.55	442.02	0.57	0.56	0.55	0.54
	std	2.34	0.34	10.46	168.52	0.21	0.21	0.21	0.2

Figure 6 shows that the wind speeds stayed in the moderate range (~ 1–3 m/s) during the experiment. Under such conditions, particle transport and deposition mechanisms are likely to dominate over aerodynamic removal, explaining the observed positive correlation between wind speeds and soiling loss.

The abrupt increase in relative humidity as shown in Fig. 7 accompanied by a drop in ambient temperature corresponds to short-duration high-humidity events commonly observed during the winter season in the Indo-Gangetic Plain. These events are typically associated with fog formation, condensation, or transient cloud cover rather than measurable rainfall.

The monthly data analysis from October to December 2024 shows how the performance of PV is affected by both changes in the environment and how often it is cleaned. The daily-cleaned panel (P1) consistently exhibited higher short-circuit current values, while progressive reductions were observed in P2, P3, and most significantly in P4, reflecting the cumulative effect of soiling at longer cleaning intervals. The variability of irradiance, indicated by higher standard deviation values, further underscores the dynamic operating environment of the panels. These findings confirm that both environmental conditions and soiling accumulation substantially impact PV output, thereby establishing the need for predictive models. Accordingly, the subsequent section develops empirical models to express.

$\:{I}_{SC\:}$as a function of GHI, AT, RH, and WS, and to quantify soiling loss as a function of RH, WS, AT, and CF, thus providing a foundational framework for later comparison with machine learning models.

## Empirical modelling of PV performance and soiling loss

The experimental dataset was utilised to derive empirical regression models for short-circuit current and soiling loss in percentage, using global horizontal irradiance, ambient temperature, wind speed, relative humidity, and cleaning frequency as predictors. Multiple linear regression is adopted to establish these relationships.

### Empirical model for short circuit current

The regression Eq. (1) obtained for $\:{I}_{SC\:}$ is:

1 $$\:{I}_{SC}=0.0126+0.0013\times\:GHI+0.0006\times\:\mathrm{A}\mathrm{T}-0.002\times\:\mathrm{W}\mathrm{S}-0.00012\times\:\mathrm{R}\mathrm{H}-0.001059\times\:\mathrm{C}\mathrm{F}$$

Table 4 presents the estimated regression coefficients for the empirical model of short-circuit current^56^. The results clearly highlight GHI as the most dominant predictor (Estimate = 0.001265, *p* < 0.001), consistent with the physical dependence of Isc on solar irradiance. Cleaning frequency also shows a highly significant negative effect (*p* < 0.001), indicating the reduction in current with increasing days between cleaning. Ambient temperature has a small but significant positive effect, while relative humidity shows a minor negative influence. In contrast, wind speed was statistically insignificant (*p* = 0.238), confirming its limited role in determining $\:{I}_{SC\:}$.

**Table 4:** Coefficient table for the $\:{I}_{SC\:}$empirical model^56^.

Predictor	Estimate	SE	tStat	p value
Intercept	0.01255	0.004198	2.9896	0.003004
GHI	0.001265	3.94E-06	321.42	0
AT	0.000579	0.000151	3.8445	0.000149
WS	-0.00187	0.001585	-1.1817	0.23816
RH	-0.00013	5.99E-05	-2.1209	0.034678
CF	-0.00106	5.77E-05	-18.343	2.64E-52

### Empirical model for soiling loss

To further ensure model robustness, adjusted R² and residual diagnostics were evaluated before and after removing GHI. The change in adjusted R² was negligible (< 0.001), confirming that irradiance does not contribute to predictive power in the SL formulation. The removal therefore improves model parsimony without compromising explanatory strength, consistent with regression theory principles. The regression Eq. (2) for soiling loss (SL) expressed as,

2 $$\:SL\left(\%\right)=0.15-0.1\times\:AT+0.45\times\:WS+0.024\times\:RH+0.18\times\:CF$$

Table 5 presents the estimated regression coefficients for the empirical model of of soiling loss show that cleaning frequency is the most significant predictor (Estimate = 0.18487, *p* < 0.001), highlighting the strong impact of longer cleaning intervals on increased soiling losses. Relative humidity also exhibits a significant positive influence (*p* = 0.007), which may be attributed to dust adhesion and cementing effects under humid conditions. Ambient temperature has a significant negative effect (*p* < 0.001), suggesting that higher temperatures may reduce relative deposition or increase self-cleaning effects. Wind speed shows a near-significant positive influence (*p* = 0.048), reflecting its dual role in either removing or redistributing dust. In contrast, GHI is statistically insignificant (*p* = 0.989), indicating that irradiance itself does not directly drive soiling loss but instead affects PV output through $\:{I}_{SC}$.

To further ensure model robustness, adjusted R² and residual diagnostics were evaluated before and after removing GHI. The change in adjusted R² was negligible (< 0.001), confirming that irradiance does not contribute to predictive power in the SL formulation. The removal therefore improves model parsimony without compromising explanatory strength, consistent with regression theory principles.

**Table 5:** Coefficient for the SL (%) empirical model.

Predictor	Estimate	*p* value
Intercept	0.158	–
AT	-0.104	< 0.001
WS	0.462	0.04–0.05
RH	0.0251	< 0.01
CF	0.186	< 0.001

## Soiling loss (%) predictive machine learning modelling

The performance of the four PV modules was evaluated in terms of short-circuit current (Isc), soiling ratio (SR) and soiling loss (SL%). The SR and SL can be computed using I_SC_ as mention below Eqs. (3) and (4) as,

3 $$\:SR=\frac{{Isc}_{(soiled\_}}{{Isc}_{\left(clean\right)}}$$

Where I_sc soiled_ is the short-circuit current of the soiled panel and I_sc clean_ is that of the clean reference panel (P1). The soiling loss percentage (SL%) was calculated as,

4 $$\:SL\left(\%\right)=\frac{{Isc}_{\left(clean\right)}-{Isc}_{\left(soiled\right)}}{{Isc}_{\left(clean\right)}}\times\:100$$

To further improve predictive accuracy beyond the empirical formulations, machine learning algorithms were employed using the experimental dataset described in Sect. 3. The empirical SL model achieved a high determination coefficient (R^2^ = 0.978) with low RMSE and MAE; however, the mean absolute percentage error (MAPE = 28%) remained relatively high, reflecting systematic nonlinearities and residual bias. To address these limitations, an ML-based predictive framework was developed.

### Input features and pre-processing

**Fig. 8:**
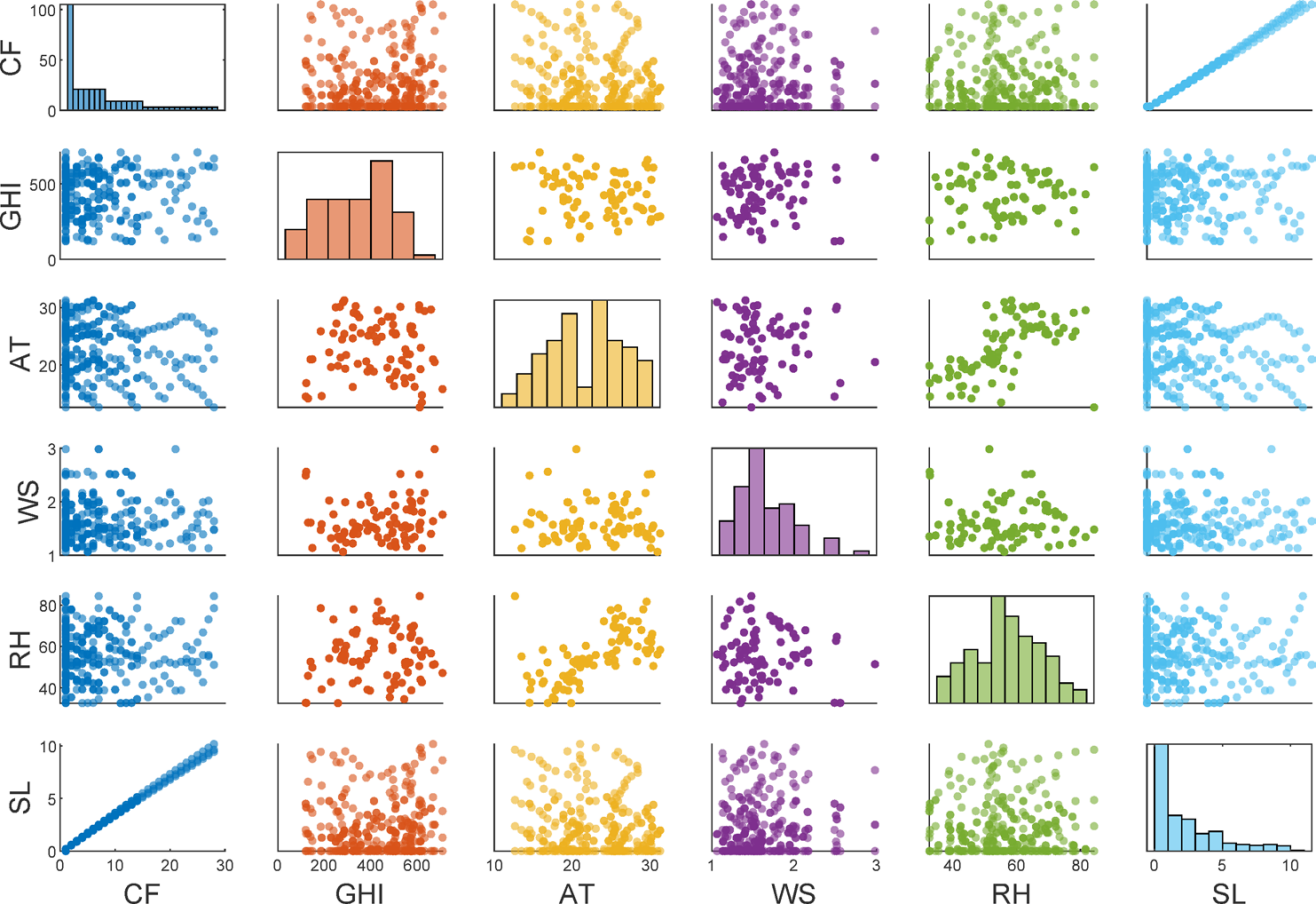
Pairwise matrix of experimental features and relationship with soiling losses.

The experimental dataset consist of both environmental and operational parameters: global horizontal irradiance (GHI), ambient temperature (AT), wind speed (WS), relative humidity (RH), reference short-circuit current of the clean panel ($\:{I}_{SC\:\left(Clean\right)}$) and cleaning frequency (CF). Categorical variables such as cleaning interval were encoded as hot encoding method, while all continuous features were standardized to zero mean and unit variance. To assess the relative contribution of selected input variables, a sensitivity analysis using the CAM approach was carried out under the Sect. 2.4. The Fig. 8 presents the scatter matrix of the selected features (CF, GHI, AT, WS, RH, and SL), excluding the month variable. In contrast to the off-diagonal scatter plots, which represent the pairwise correlations between the parameters, the diagonal histograms illustrate the distribution of each parameter.

Global horizontal irradiance, ambient temperature, relative humidity, and wind speed have substantial temporal autocorrelation, making subsequent measurements not statistically independent. Randomly mixing time-dependent samples destroys temporal structure and leaks temporal data, enabling the model to indirectly learn patterns from subsequent observations. This may result in inappropriately optimistic assessment outcomes and exaggerated performance measures (e.g., R²). To evade this, the dataset was divided into 80% training and 20% testing observations using a time-ordered split. This method retains temporal causality and provides realistic model generalization in practice.

**Fig. 9:**
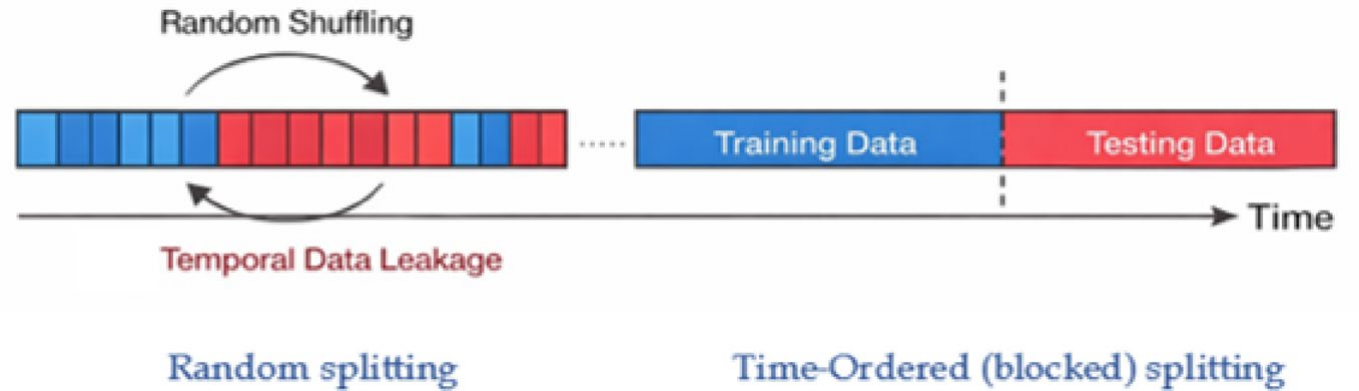
Time-ordered data splitting for temporally correlated environmental data.

Figure 9 compares random and time-ordered (blocked) data splitting for temporally auto-correlated environmental variables (GHI, RH, AT, WS). Random splitting leaks temporal data and inflates performance measures by include samples from comparable time periods in practice and testing. Time-ordered splitting conserves chronology and enables realistic generalization.

#### Cosine amplitude method

In this work, the cosine amplitude method (CAM) was used to assess the correlation between the input and output data. The mathematical formulation of CAM given in Eq. (5).

5 $$\:CAM\:\left({X}_{i},Y\right)=\frac{\sum\:_{j=1}^{n}{X}_{ij}{Y}_{j}}{\sqrt{{\sum\:}_{j=1}^{n}{X}_{ij}^{2}\:}\cdot\:\:\sqrt{\sum\:_{j=1}^{n}{Y}_{j}^{2}}}$$

**Fig. 10:**
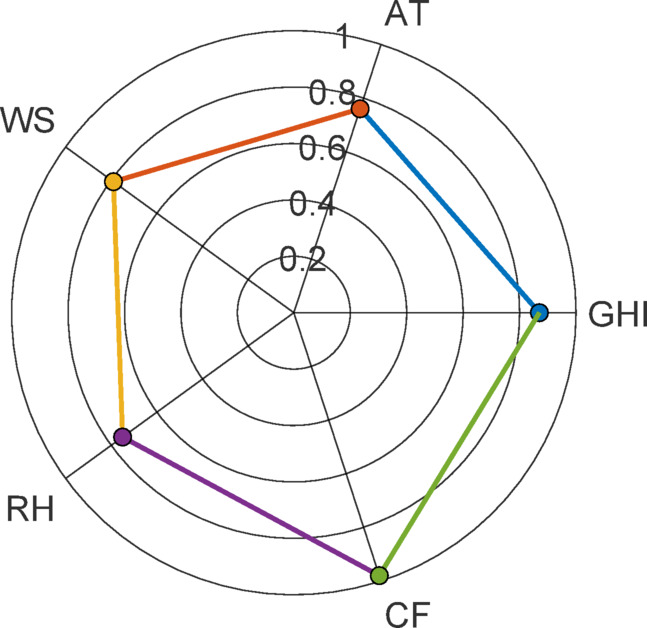
Cosine amplitude scores (CAM) for feature importance.

There is a connection between the cosine function and the dot product, as shown by Eq. (3). Whereas the inner product of two vectors where Xi input vector while Y is the output vector equal to zero when they are at right angles to one another, the product of two vectors that are collinear is equal to one. A greater directional similarity (and hence sensitivity) to the output is seen by features that have higher CAM scores (closer to 1) than those with lower scores as shown in Fig. 10.

CAM is the cosine similarity between the features (GHI, AT, WS, RH and CF) and target vectors soiling loss (%) is scale-invariant. The Fig. 9 indicate that the CF is the strongest driver of soiling loss in comparison with meteorological variables which shows moderate CAM score.

To evaluate potential multicollinearity among environmental predictors, the Variance Inflation Factor (VIF) was computed for GHI, RH, and AT. The obtained VIF values were 1.023 (GHI), 1.5987 (RH), and 1.5809 (AT), all of which are substantially below the threshold value of 5. These results confirm the absence of significant multicollinearity and demonstrate that the regression coefficients are stable and not adversely affected by linear dependency among predictors.

#### Permutation-based feature importance analysis

**Fig. 11:**
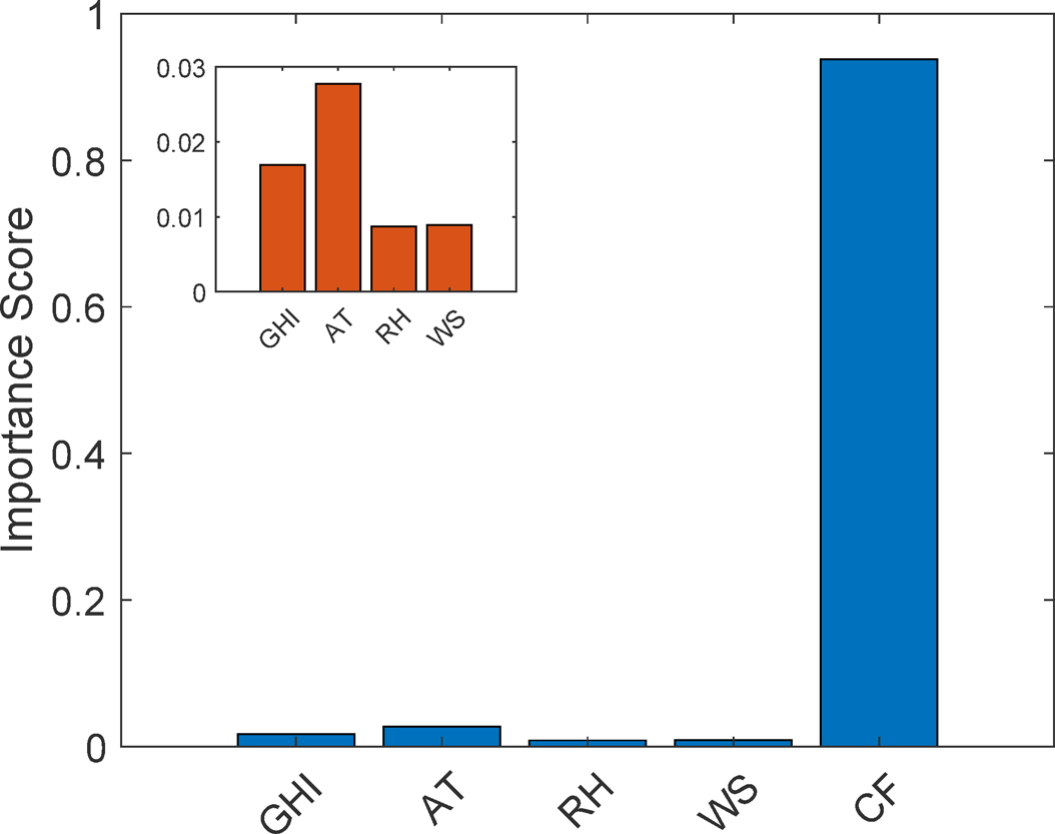
Permutation-based feature importance analysis.

To further validate feature sensitivity, permutation importance analysis^57^ was performed, as shown in Fig. 11 Cleaning frequency was identified as the dominant predictor of soiling loss, consistent with physical dust accumulation mechanisms. Environmental variables such as ambient temperature, relative humidity, irradiance, and wind speed exhibited smaller but meaningful contributions. The inset plot provides a detailed view of environmental feature importance. These findings confirm the robustness and physical consistency of the predictive model.

### Machine learning methods

After pre-processing data, soiling loss are estimated using various method of ML such as DT, KNN, SVM, ANN and Stacking. The stacking ensemble combined ANN, SVM, and DT as base learners, with a gradient boosting regressor (GBR) as the meta-learner. GBR effectively refined the base predictions by capturing residual nonlinear patterns, resulting in improved accuracy and reduced bias. MATLAB R2024 a is used to run simulations on a Dell laptop, featuring a Core i9-11900 H processor and 32 GB of RAM.

#### Artificial neural network

**Fig. 12:**
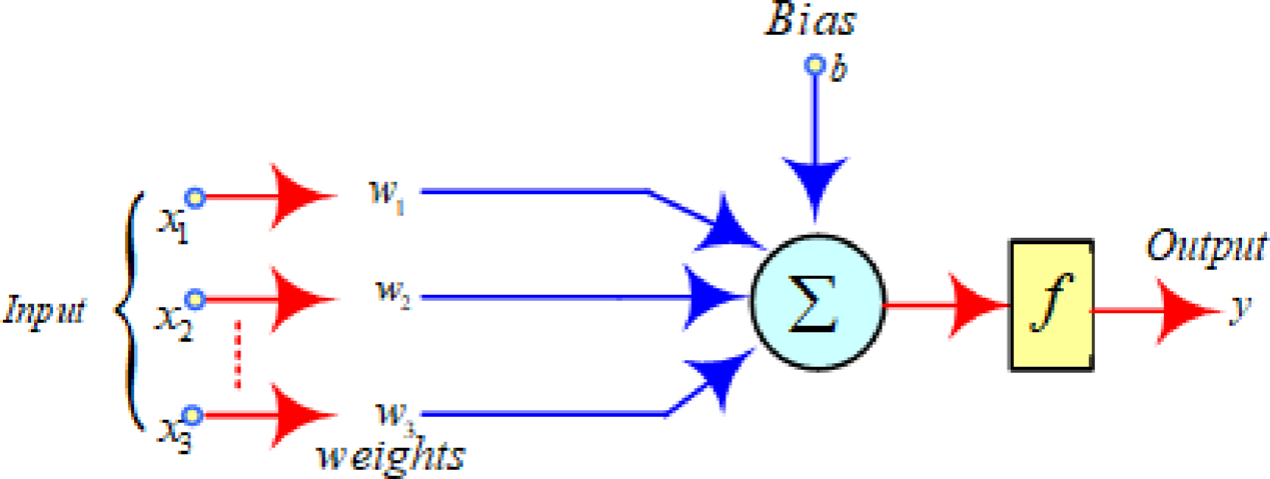
ANN model.

ANN is intended to replicate the neuronal organisation of the human brain by employing layers that are interconnected in order to capture complicated interactions, as seen in (Fig. 12)^58,59^. Backpropagation is used to train the model in this study in order to minimize the errors. In given Eq. (6) w_n_ are representing weights corresponding to each inputs x_n_ and b is the bias, while final predicted output represented by Y.

6 $$\:Y={w}_{1}\times\:{x}_{1}+{w}_{2}\times\:{x}_{2}+\cdots\:\cdots\:\cdots\:{w}_{n}\times\:{x}_{n}+b$$

#### Support vector machine

**Fig. 13:**
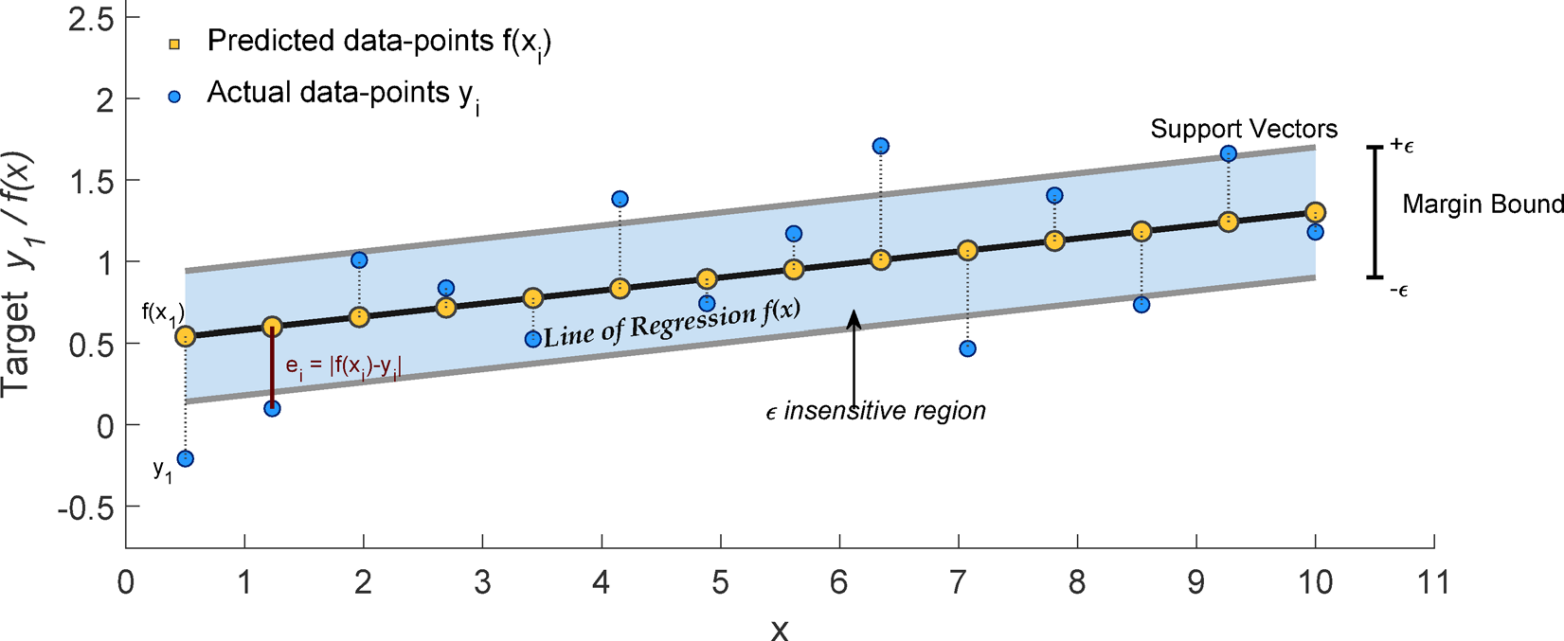
Support vector machine (regressor).

Figure 13 shows how support vector machine (SVM) uses kernel functions to divide data in high-dimensional regions and capture complicated connections for classification and regression^60^. It estimates SL in this work and expressed in Eq. (7) as,

7 $${\mathrm{Y}} = {\mathrm{W}}^{{\mathrm{T}}} \times {\mathrm{Z}} + {\mathrm{B}}$$

where, Z is the input vector, W is the weight and B is the bias term.

#### Regression tree (RT)

**Fig. 14:**
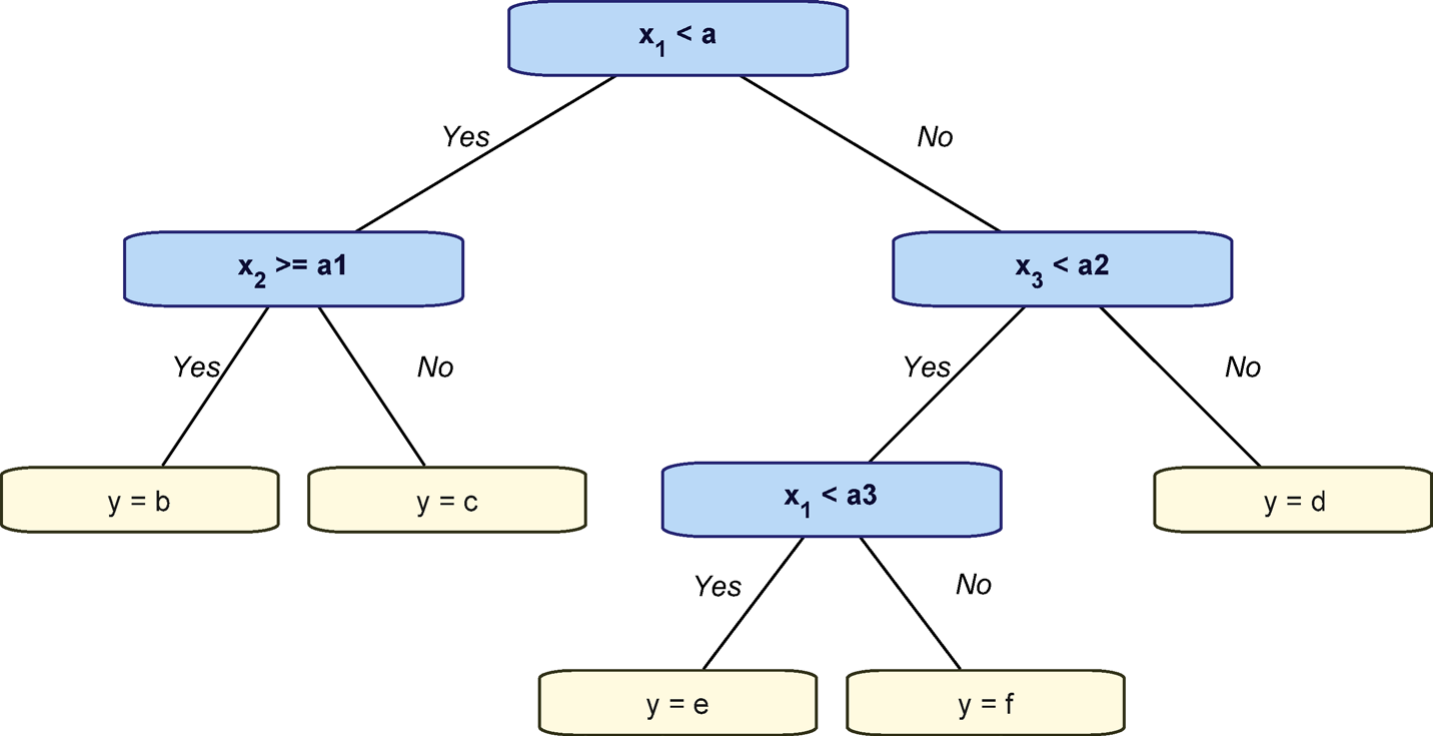
Decision tree (regressor).

RT, as seen in Fig. 14, are decision trees used to forecast continuous variables SL by segmenting data according to defined criteria and computing the mean target value for each subgroup. They are interpretable, resilient to outliers, and adept at managing non-linear connections successfully^61^. The proposed approach employs regression trees by segmenting the feature space and predicting the target variables SL inside each segment as mentioned in Eq. (8).

8 $$\:\widehat{Y}\left(X\right)=\sum\:_{n=1}^{N}{C}_{n}\times\:I\left(X\in\:{Z}_{n}\right)$$

In a regression tree, N is the total number of nodes (leaves), each region Z_n_ represents a partition of the feature space, C_n_ is the mean target value within that region, and I (X∈Zm) is an indicator function that equals 1 if X belongs to Z_n_ otherwise 0.

#### K-nearest neighbours

**Fig. 15:**
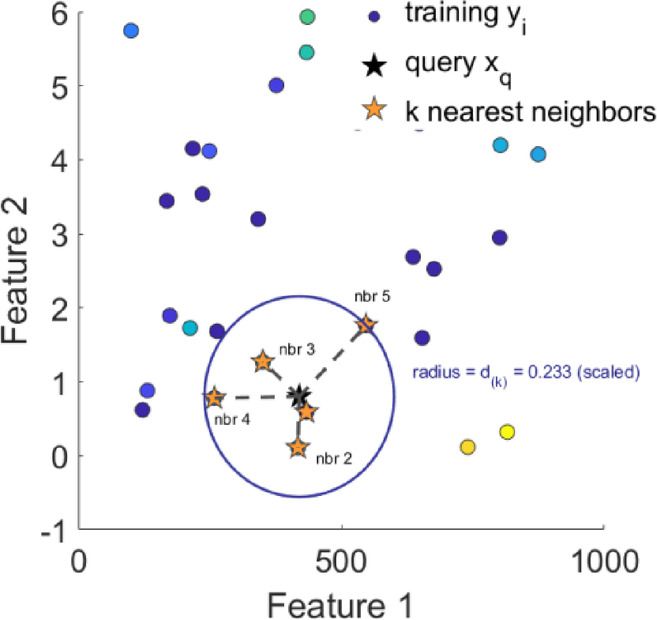
K-nearest neighbours.

The K-nearest neighbours (KNN) algorithm is a simple, non-parametric method that predicts outputs based on the average of the k closest data points in the feature space as shown in Fig. 15. In the context of this work, KNN estimates soiling loss by finding similar conditions of GHI, AT, WS, CF and RH from experimental data. It is intuitive and effective for capturing local patterns without requiring an explicit training phase.

9 $$\:\widehat{Y}\:\left(X\right)=\frac{1}{K}\sum\:_{i\in\:{N}_{K}\left(X\right)}{Y}_{i}$$

In KNN regression equation where K is the number of nearest neighbours, N_K_(X) is the set of those neighbours, and Y_i_​ are their target values, making the prediction the average of the *K* closest points.

#### Stacking model

**Fig. 16:**
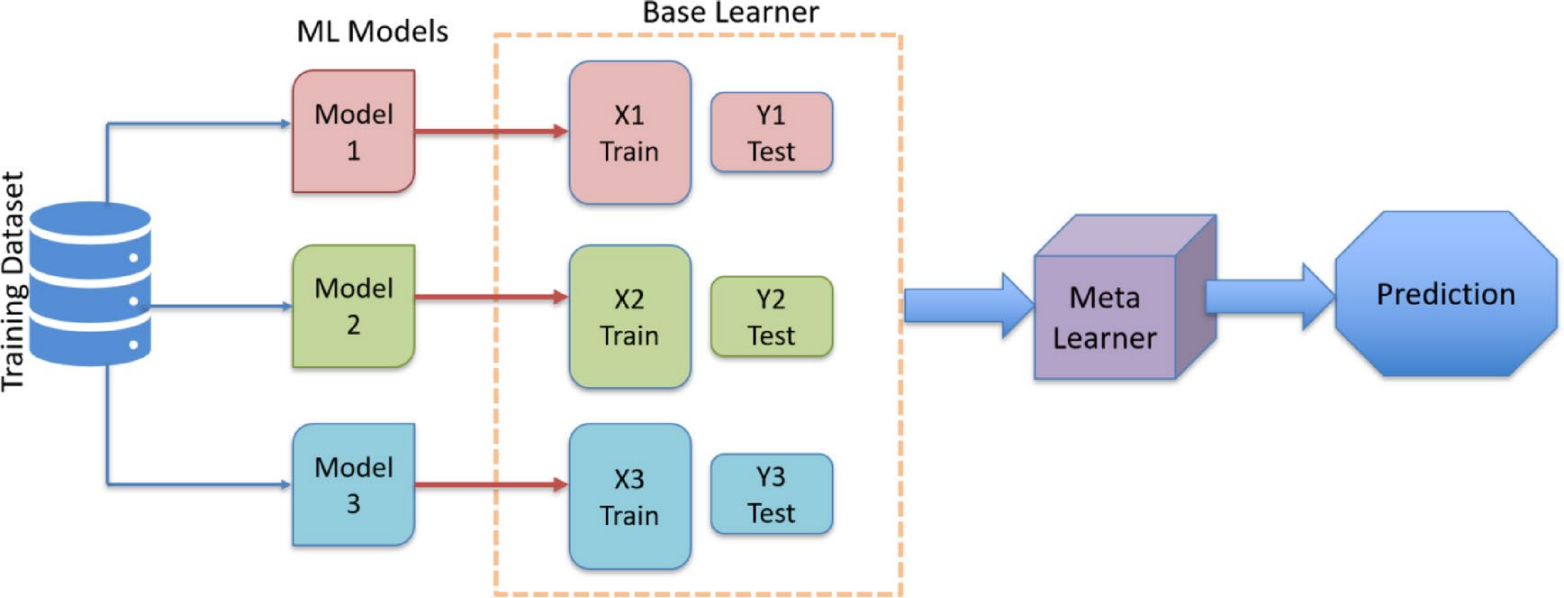
Stacking ensemble model.

The stacking model is an ensemble learning approach that combines multiple base learners to improve predictive performance shown in Fig. 16. In this work, ANN, SVM, and DT were used as base learners to capture diverse data patterns, and their outputs were blended by a Gradient Boosting Regressor (GBR) as the meta-learner. This framework leverages the strengths of each individual model while compensating for their weaknesses. As a result, the stacking model achieved higher accuracy and robustness compared to single-model approaches.

For the L base learner the stacking prediction given in Eq. (10) as,

10 $$\:\widehat{y}\left(x\right)=g\left({m}_{1}\left(x\right),{m}_{2}\left(x\right)\cdots\:{m}_{L}\left(x\right)\right)$$

where m_L_ (x) are the base learner outputs (ANN, SVM, DT in this work) and g(⋅) is the meta-learner (GBR) that combines them to produce the final output.

**Fig. 17:**
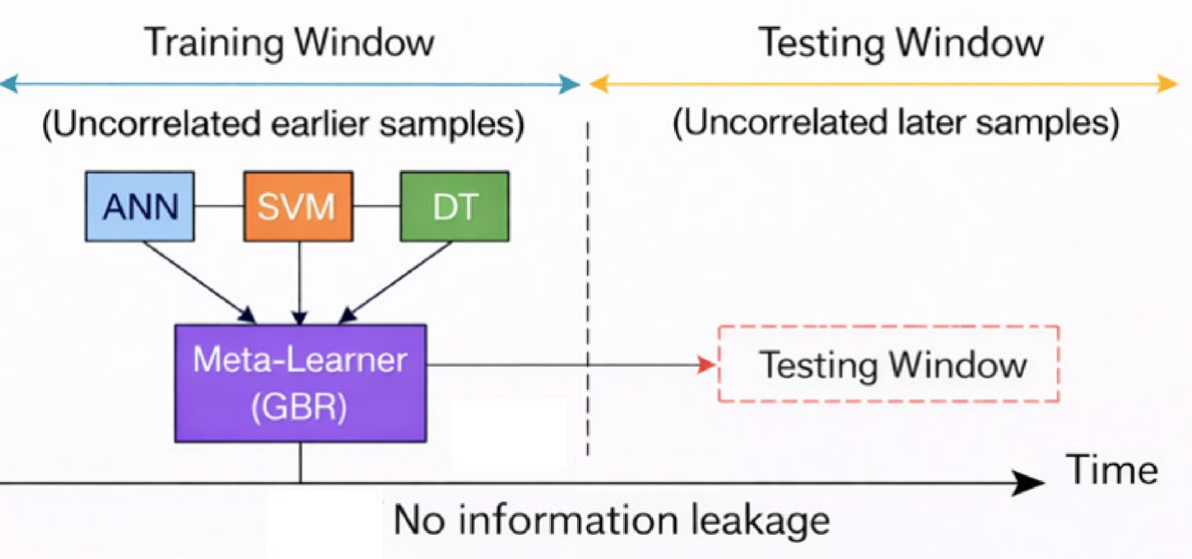
Time-aware training of stacking ensemble without cross-validation leakage.

In this study, conventional random k-fold cross-validation was not employed because the dataset represents a physically time-ordered environmental process. Environmental variables such as irradiance, temperature, humidity, and wind speed exhibit strong temporal autocorrelation and causal continuity. Randomized cross-validation would mix past and future observations, introducing information leakage and leading to overly optimistic performance estimates, particularly for stacking models where the meta-learner learns second-order correlations. To ensure physically realistic and leakage-free validation, a time-ordered training–testing strategy was adopted. As illustrated in the Fig. 17, the dataset is divided chronologically into a training window (earlier observations) and a testing window (later unseen observations). The base learners (ANN, SVM, and DT) were trained exclusively on the training window, and their predictions were used to train the meta-learner (Gradient Boosting Regressor). The trained stacking model was then evaluated only on the testing window, which contained future unseen samples.

### Hyperparameter optimization of machine learning model

The hyperparameters of all machine learning models, including ANN, SVM, DT, KNN, and the GBR used in the stacking ensemble, were selected using a systematic tuning procedure based on grid search combined with validation on the training dataset^62^. The hyperparameter combinations presented in Table 6 which is used to all machine learning model in order to minimized prediction error while avoiding overfitting. For each model, a range of candidate hyperparameters was evaluated, and the optimal configuration was selected based on minimum RMSE and stable generalization performance. The same training dataset and evaluation criteria were applied consistently across all models to ensure fair comparison.

**Table 6:** Optimized hyperparameters.

Model	Hyperparameter	Value
ANN	Hidden layers	1
	Neurons	10
	Training algorithm	Levenberg–Marquardt
	Activation function	ReLU / tanh
	Epochs	1000
SVM	Kernel function	Gaussian (RBF)
	Box constraint	1
	Kernel scale	Auto
DT	Maximum splits	20
	Minimum leaf size	4
KNN	Number of neighbours (k)	5
	Distance metric	Euclidean
GBR (meta learner)	Number of learners	100
	Learning rate	0.1
	Maximum splits	10

### Performance matrix

It is important to evaluate the precision of the prediction model. A variety of measures have been used to evaluate the precision of predicting PV output power production^12^, which include:

(a) Mean Absolute Error (MAE): Computes the average of absolute differences between actual and predicted values, giving equal weight to all errors as expressed in Eq. (11)

11 $$\:\mathrm{MAE\:}=\frac{1}{N}\sum\:_{t=1}^{N}\:\:\left|{W}_{\mathrm{Power\:Forecasted\:}}-{W}_{\mathrm{Power\:Observed\:}}\right|$$

(b) Mean Square Error (MSE): Measures the average of squared differences between actual and predicted values, penalizing larger errors more, its mathematical expression mention in Eq. (12)

12 $$\:\mathrm{MSE\:}=\frac{1}{N}\sum\:_{t=1}^{N}\:\:{\left({W}_{\mathrm{Power\:Forecasted\:}}-{W}_{\mathrm{Power\:Observed\:}}\right)}^{2}$$

(c) Root Mean Square Error (RMSE): Square root of MSE, expressing as Eq. (13) prediction error in the same units as the target variable.

13 $$\:\mathrm{RMSE\:}=\sqrt{\frac{1}{N}\sum\:_{t=1}^{N}\:\:{\left({W}_{\mathrm{Power\:Forecasted\:}}-{W}_{\mathrm{Power\:Observed\:}}\right)}^{2}}$$

(d) Coefficient of Determination (R^2^): Indicates how much variance in the actual data is explained by the model, with values closer to 1 showing better fit.The expression shown below in Eq. (14)

14 $$\:{R}^{2}\mathrm{\:-score}=1-\:\left(\frac{\sum\:_{t=1}^{N}\:\:\left|{W}_{\mathrm{Power\:Observed\:}}-{W}_{\mathrm{Power\:Forecasted\:}}\right|}{\sum\:_{t=1}^{N}\:\:\left|{W}_{\mathrm{Power\:Observed\:\:}}-{W}_{\mathrm{Power\:Observed\:\:}}\right|}\right)$$

(e) Mean Absolute Percentage Error (MAPE): Represents as shown in Eq. (15) the average absolute error as a percentage of actual values, useful for relative accuracy.

15 $$\:MAPE=\frac{1}{n}{\sum\:}_{k=1}^{n}|\frac{{W}_{\mathrm{Power\:Forecasted\:}}-{W}_{\mathrm{Power\:Observed\:}}}{{W}_{\mathrm{Power\:Observed\:}}}|$$

## Results and discussion

This section discusses the performance and findings of the suggested models. The testing findings under actual environmental condition from the Roorkee area, India, are also given according to month and cleaning frequency. The empirical model produced from the experimental data is constructed and compared with machine learning models.

### Experimental PV performance trends and variability analysis

The performance of the four PV modules was evaluated in terms of short-circuit current (Isc), soiling ratio (SR), soiling loss (SL%), and current–voltage (I–V) and power–voltage (P–V) characteristics. The daily-cleaned panel (P1) was taken as the clean reference, while P2, P3, and P4 represent panels cleaned at weekly, biweekly, and monthly intervals, respectively.

Short-circuit current ($\:{I}_{SC\:}$) was adopted as the primary soiling indicator because dust accumulation predominantly reduces optical transmission, directly affecting photocurrent generation. Since Isc is approximately proportional to irradiance, it provides a linear and direct measure of optical attenuation. In contrast, power output (P_max_) incorporates nonlinear temperature and fill factor effects, which may obscure pure dust-related losses.

In addition to $\:{I}_{SC\:}$based metrics, I–V and P–V curves were generated using a calibrated PV analyzer for each panel at different cleaning intervals. These curves provide detailed insight into the effect of dust accumulation not only on the short-circuit current but also on the maximum power point (.

**Fig. 18:**
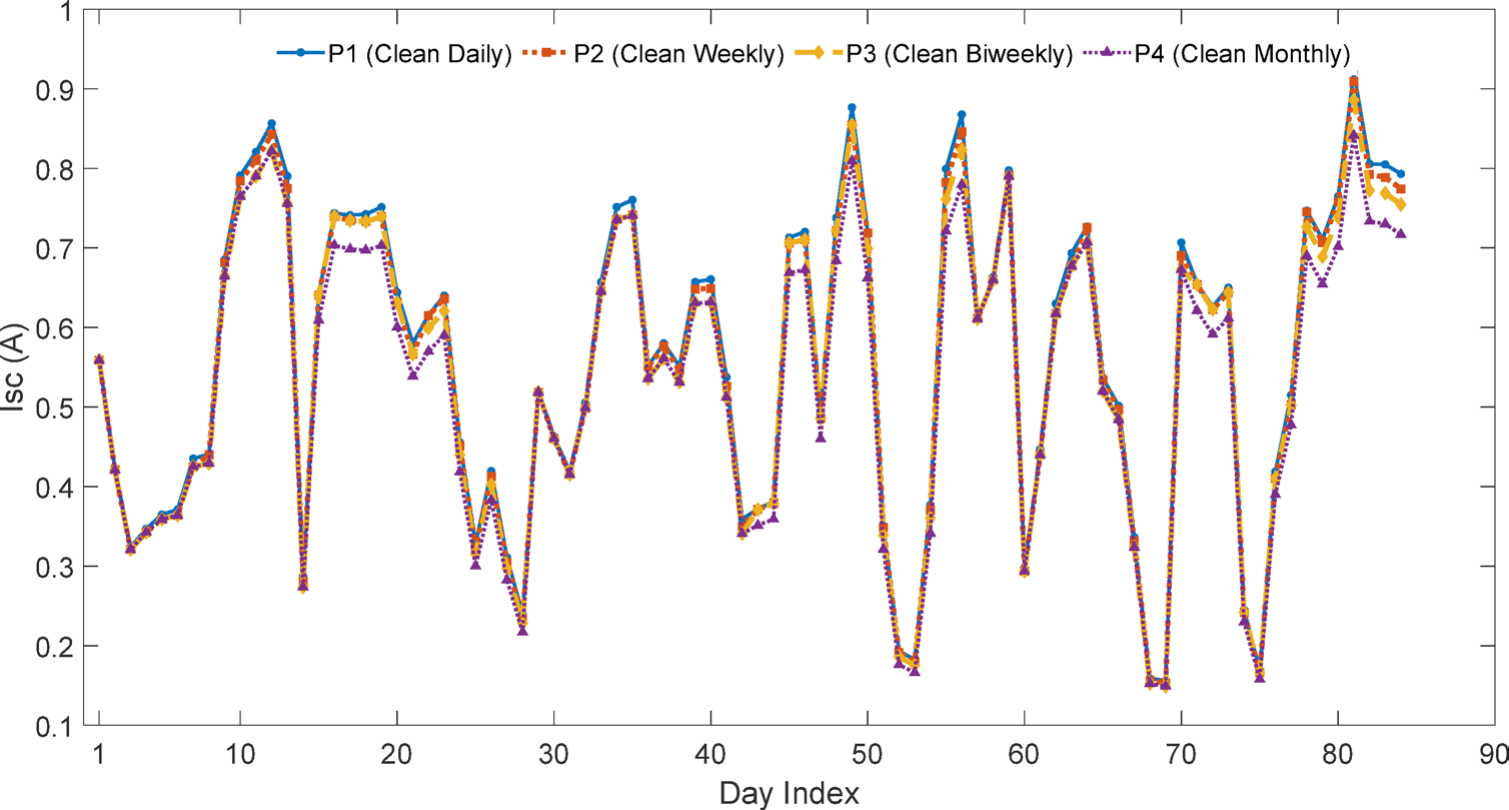
Daily average variation of short-circuit current for PV panels with different cleaning frequencies.

**Fig. 19:**
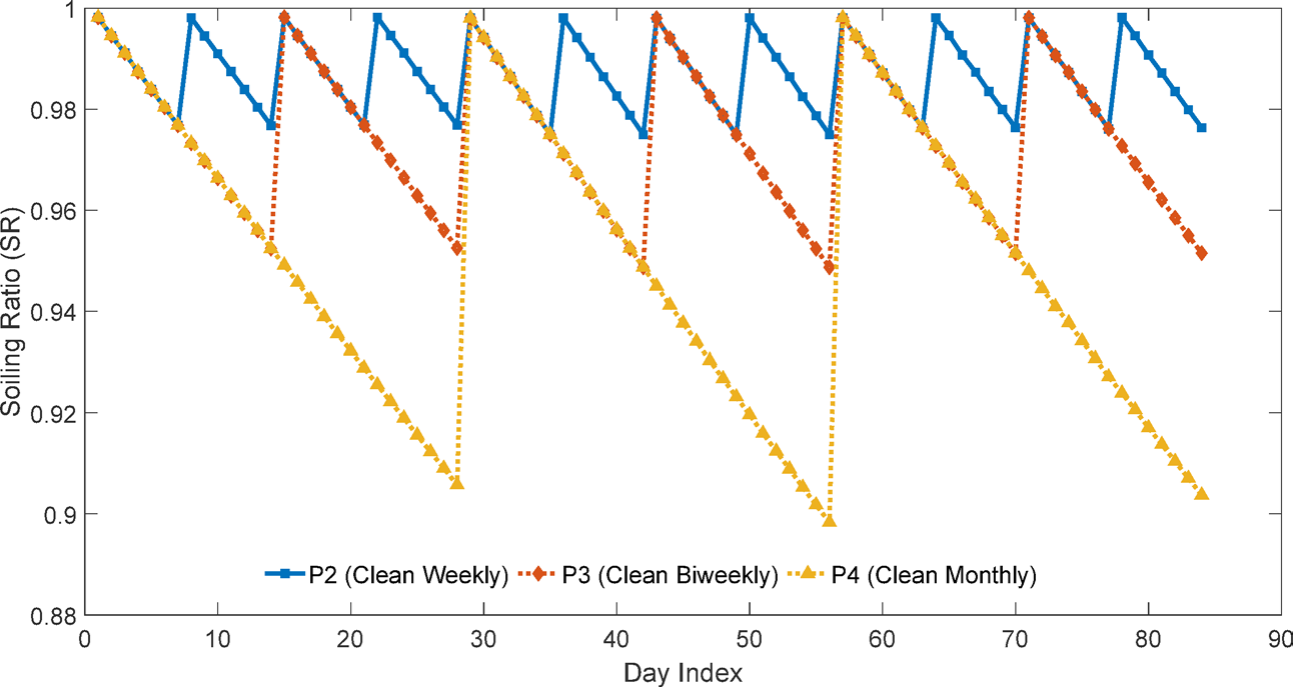
Daily variation of soiling ratio for PV panels cleaned at different intervals.

$\:{P}_{MPP}$), open-circuit voltage ($\:{V}_{oc}$), and fill factor (FF). Figures 18 and 19 shows daily average.

**Fig. 20:**
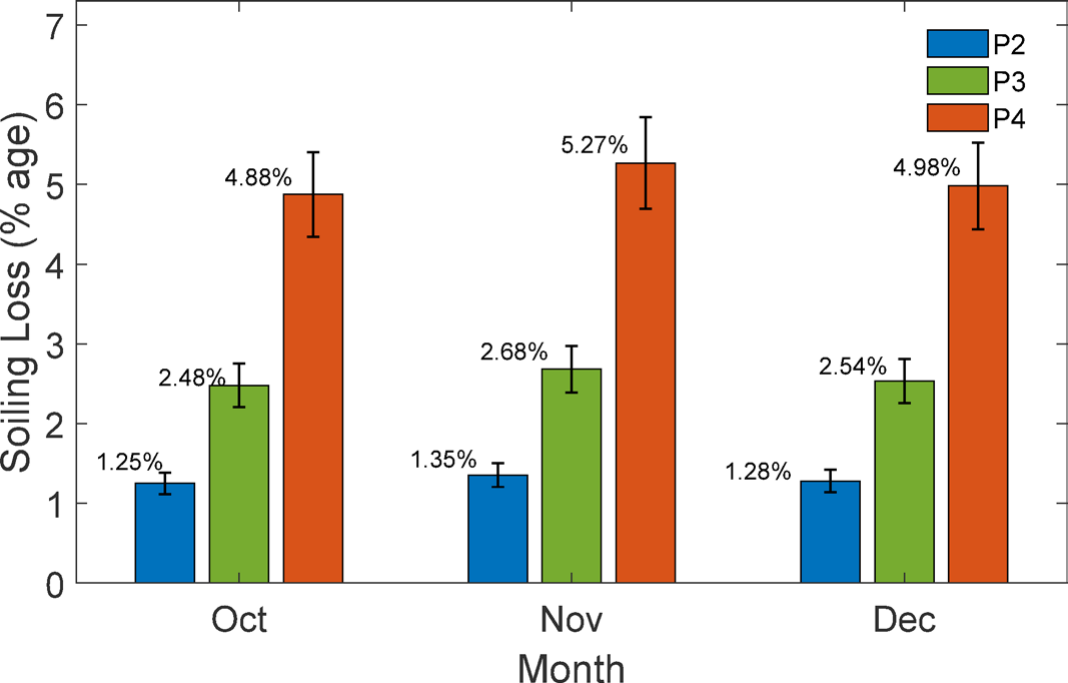
Monthly average soiling loss (%) for PV panels with different cleaning intervals: P2 (clean weekly), P3 (clean biweekly), and P4 (clean monthly).

$\:{I}_{SC\:}$ and daily soiling ratio (SR) trend over the time period of the experiment while Fig. 20 illustrate about the monthly soiling loss in percentage.

The Figs. 18, 19 and 20 collectively illustrate the impact of cleaning frequency on PV performance. The daily-cleaned panel (P1) maintained the highest $\:{I}_{SC\:}$, while P2–P4 showed progressive reductions with longer cleaning intervals. The soiling ratio (SR) exhibited a stepwise decline within each cleaning cycle, steepest for the monthly-cleaned panel (P4). Monthly average soiling losses confirmed this trend, increasing from 1 to 1.5% (P2) to 2.5% (P3) and5% (P4). These results clearly demonstrate that extended cleaning intervals accelerate dust-induced performance degradation.

**Fig. 21:**
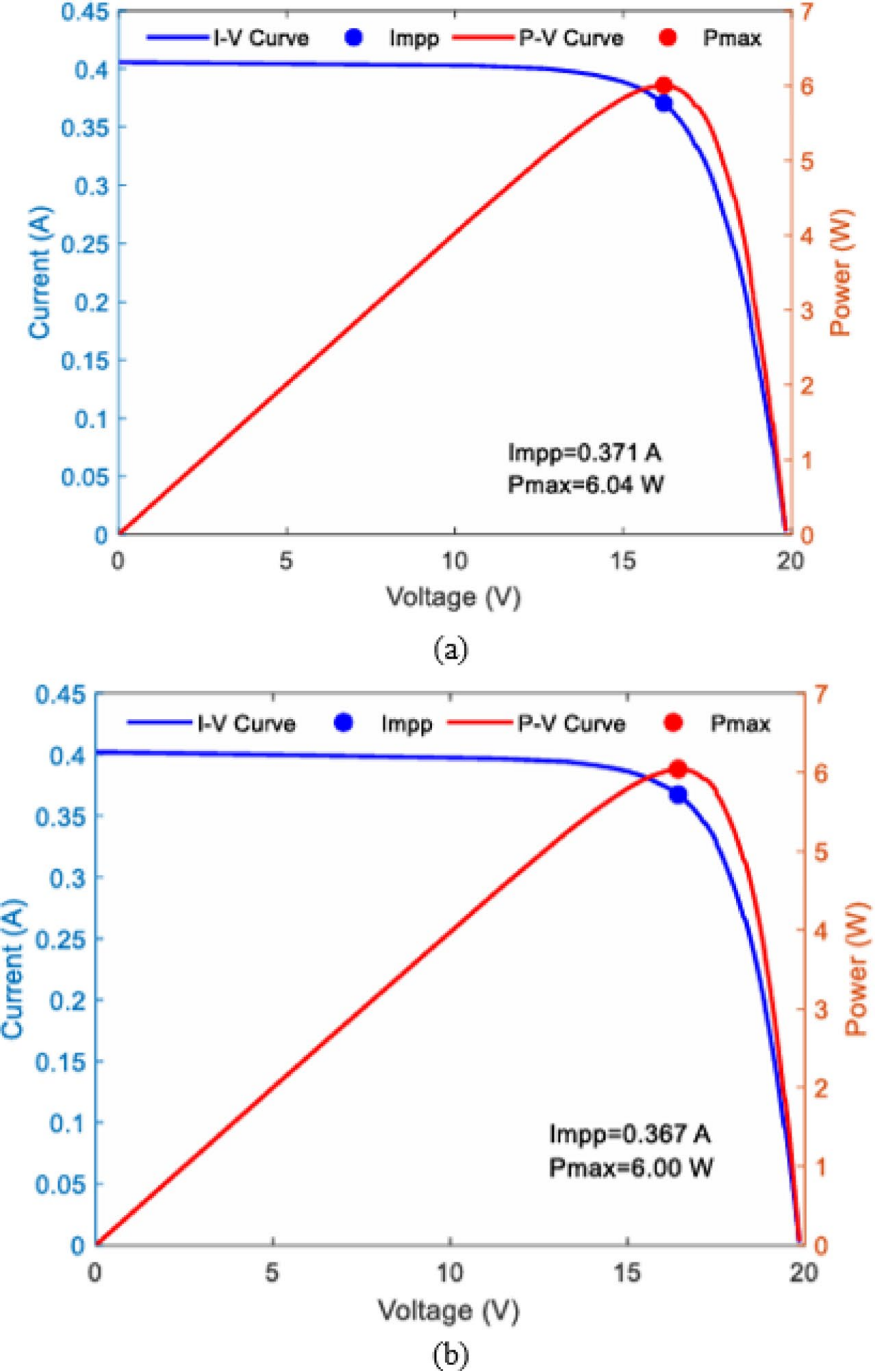

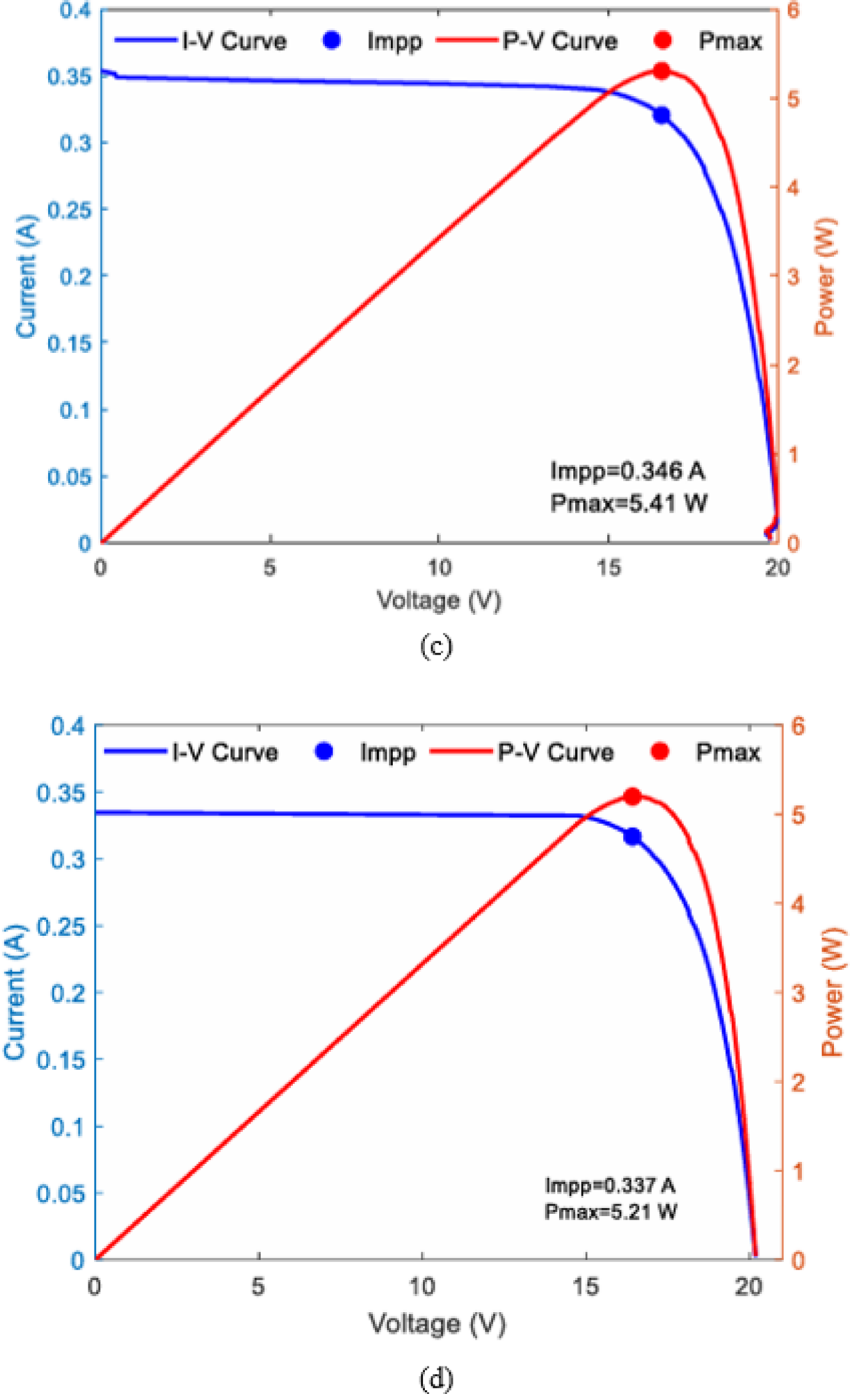
PV analyser measurement of I-V and P-V characteristics at GHI 415 w/m^2^ (**a**) P1:-clean daily (**b**) P2:- clean weekly (**c**) P3:- clean biweekly (**d**) P4:- clean monthly.

Figure 21 shows the I–V and P–V characteristics of the four PV panels under different cleaning frequencies. The clean reference panel (P1, cleaned daily) achieved the highest maximum power point ($\:{P}_{MPP}$ = 6.00 W) and current at MPP ($\:{I}_{mpp}$ = 0.370 A). Panels with reduced cleaning frequency demonstrated progressive reductions in both $\:{I}_{mpp}$ and $\:{P}_{MPP}$: P2 (weekly) produced 5.80 W, P3 (biweekly) dropped to 5.59 W, and P4 (monthly) showed the lowest performance at 5.31 W. The open-circuit voltage ( $\:{V}_{oc}$) remained relatively stable across all panels, indicating that dust accumulation primarily impacts the short-circuit current and the maximum power output.

### Performance of $\:{I}_{SC\:}$ empirical model

**Fig. 22:**
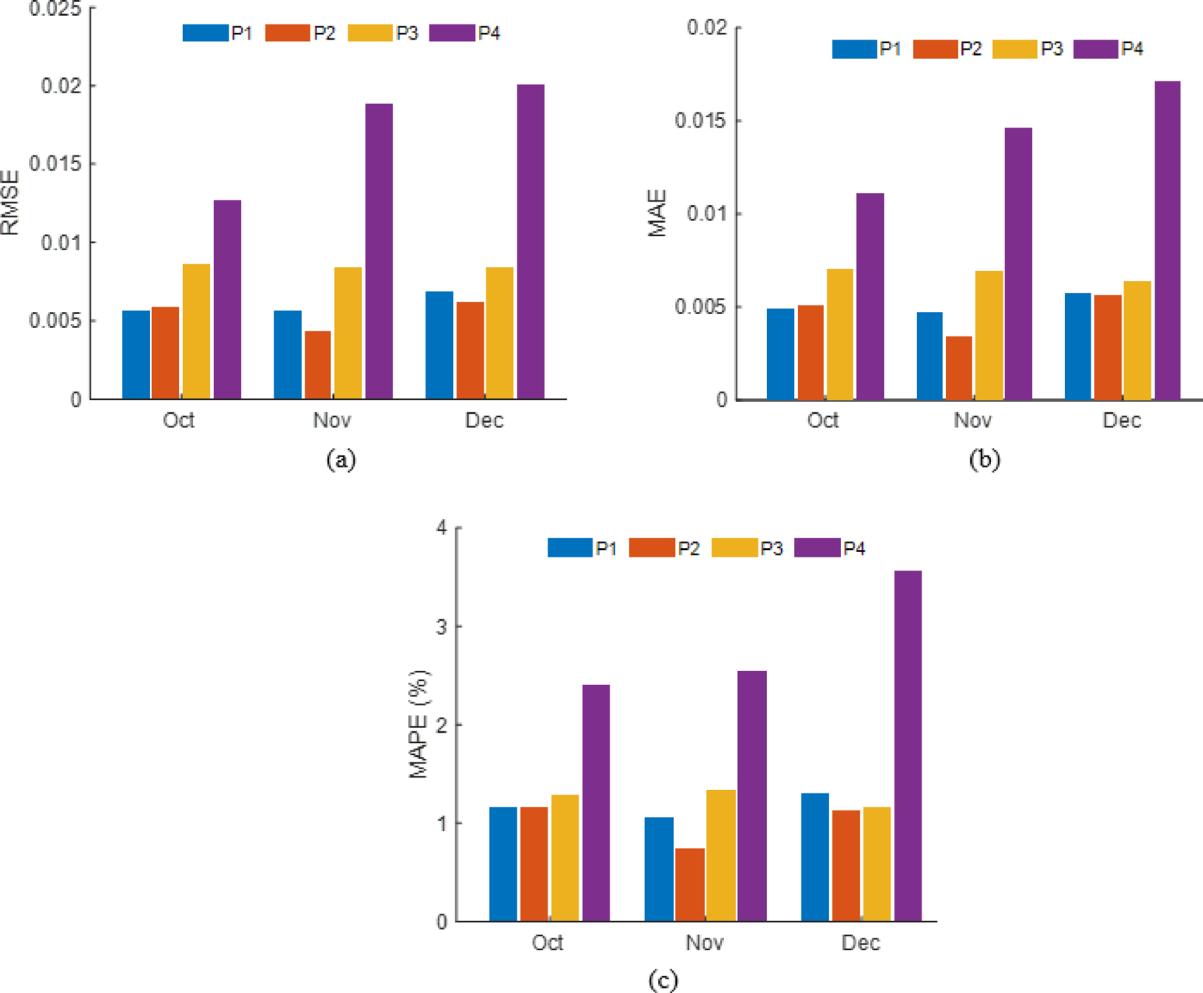
Monthly cleaning frequency wise performance evaluation of empirical model (**a**) RMSE (**b**) MAE (**c**) MAPE.

Validation performance of empirical $\:{I}_{SC\:}$ models for four PV panels under different cleaning frequencies during October–December are shown in Fig. 22 as, (a) RMSE, (b) MAE, and (c) MAPE. Panels P1–P3 (daily, weekly, and biweekly cleaning) maintained low errors (RMSE ≤ 0.009 A, MAE ≤ 0.007 A, MAPE = 1–1.5%), whereas P4 (monthly cleaning) exhibited significantly higher deviations (RMSE up to 0.020 A, MAE = 0.017 A, MAPE = 3.7% in December), highlighting the negative impact of extended cleaning intervals on model accuracy.

**Fig. 23:**
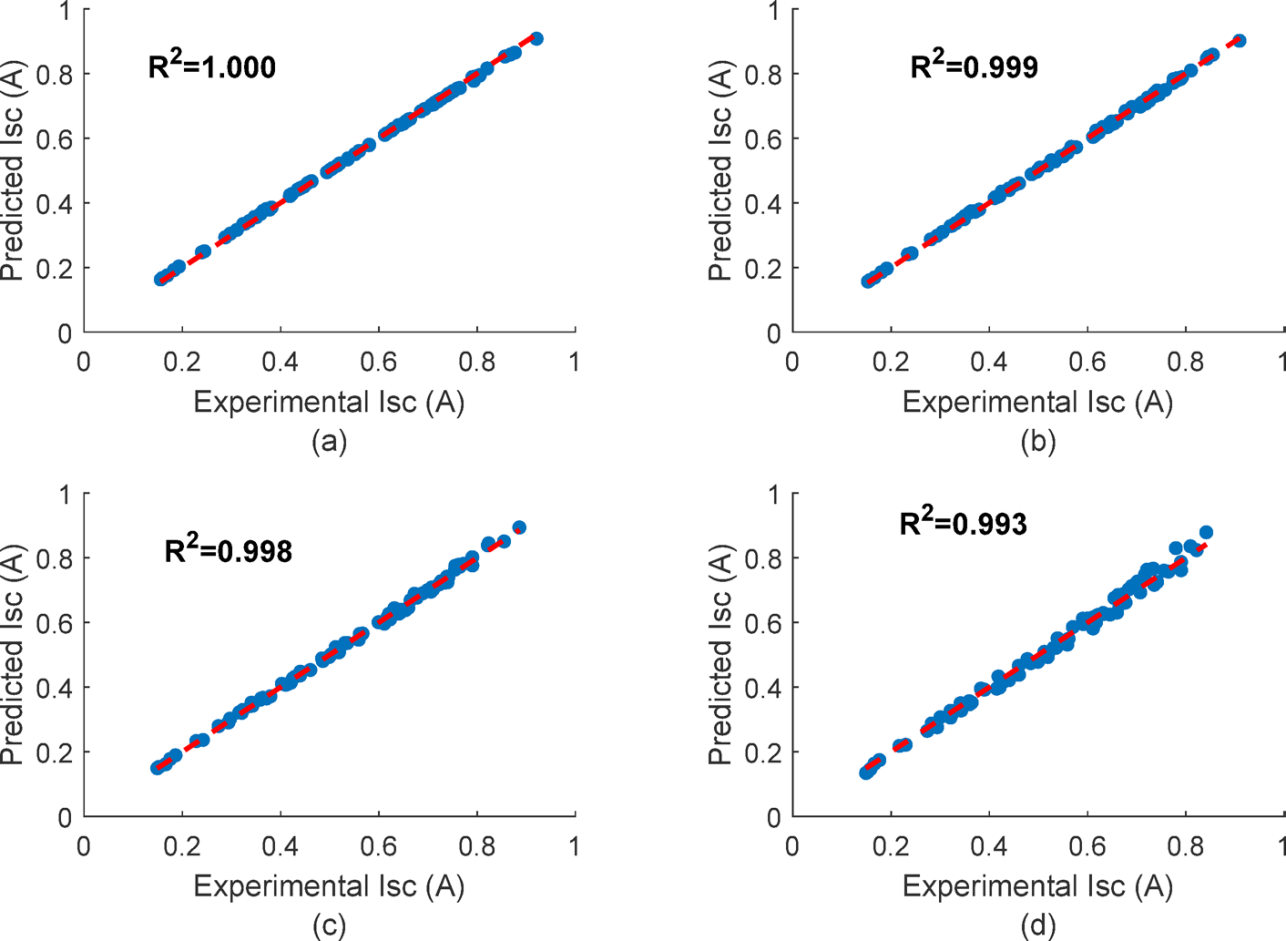
Experimental vs. Empirical model R-Squared plot (Oct-Dec 2024) of (**a**) P1:-clean daily (**b**) P2:- clean weekly (**c**) P3:- clean biweekly (**d**) P4:- clean monthly.

Figure 23 shows that the four PV panels have a R² value of 0.99 or above, proving that the empirical modelling framework is reliable for describing the changes in I_(SC) under various cleaning conditions. The gradual decline in R² with reduced cleaning frequency highlights the sensitivity of empirical models to dust accumulation patterns.

### Performance of SL empirical model

**Fig. 24:**
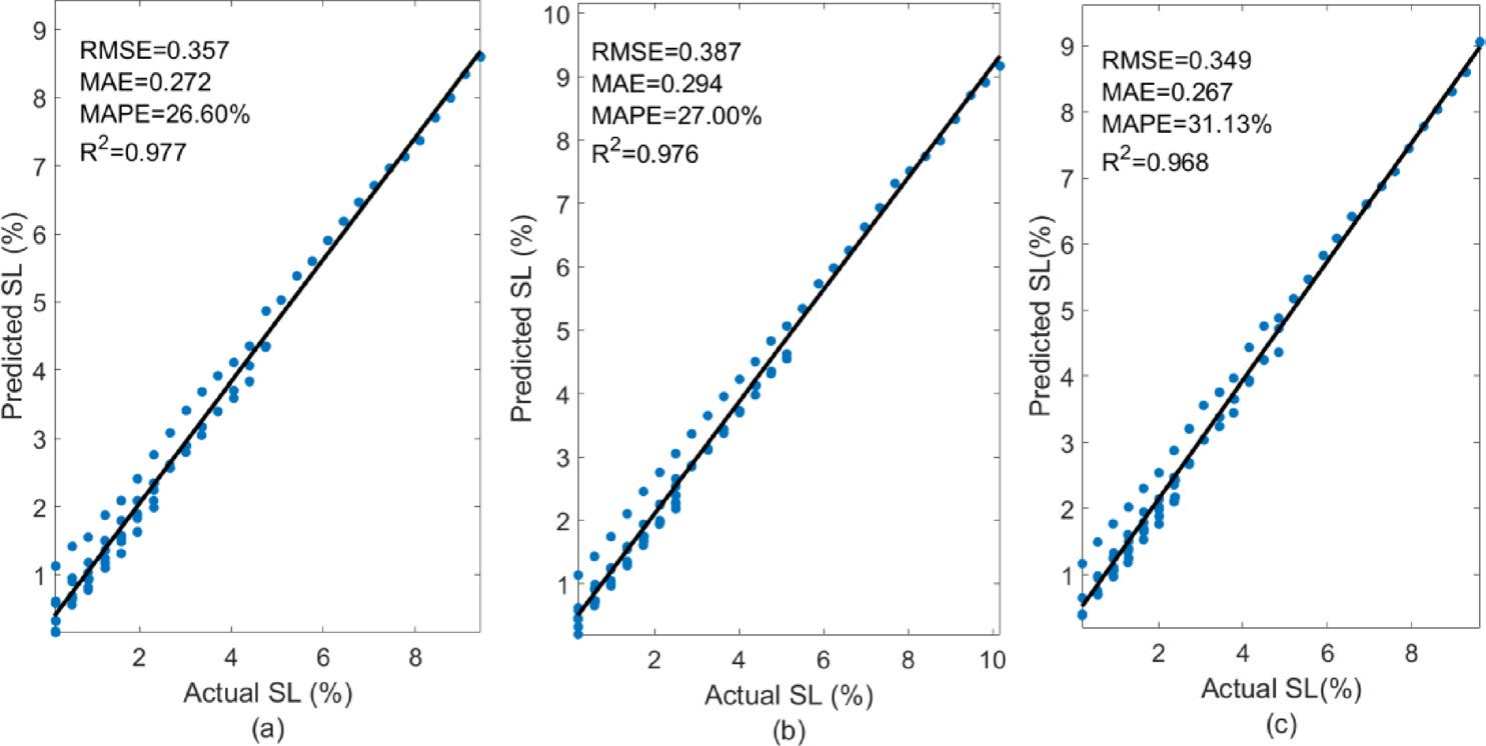
Experimental vs. empirical SL model R-squared plot for month (**a**) October (**b**) November (**c**) December.

The three PV panels are compared from October to–December to analyse the measured vs. projected soiling loss (SL, %) as shown in Fig. 24. The estimated regression line explains most of the variation (monthly R² =0.97–0.98) with minor absolute errors (RMSE = 0.35, MAE = 0.27). The moderate relative error (MAPE = 26–31%) suggests systemic bias or nonlinear effects that the basic empirical fit cannot capture. The following part uses machine-learning to lessen this relative inaccuracy.

**Fig. 25:**
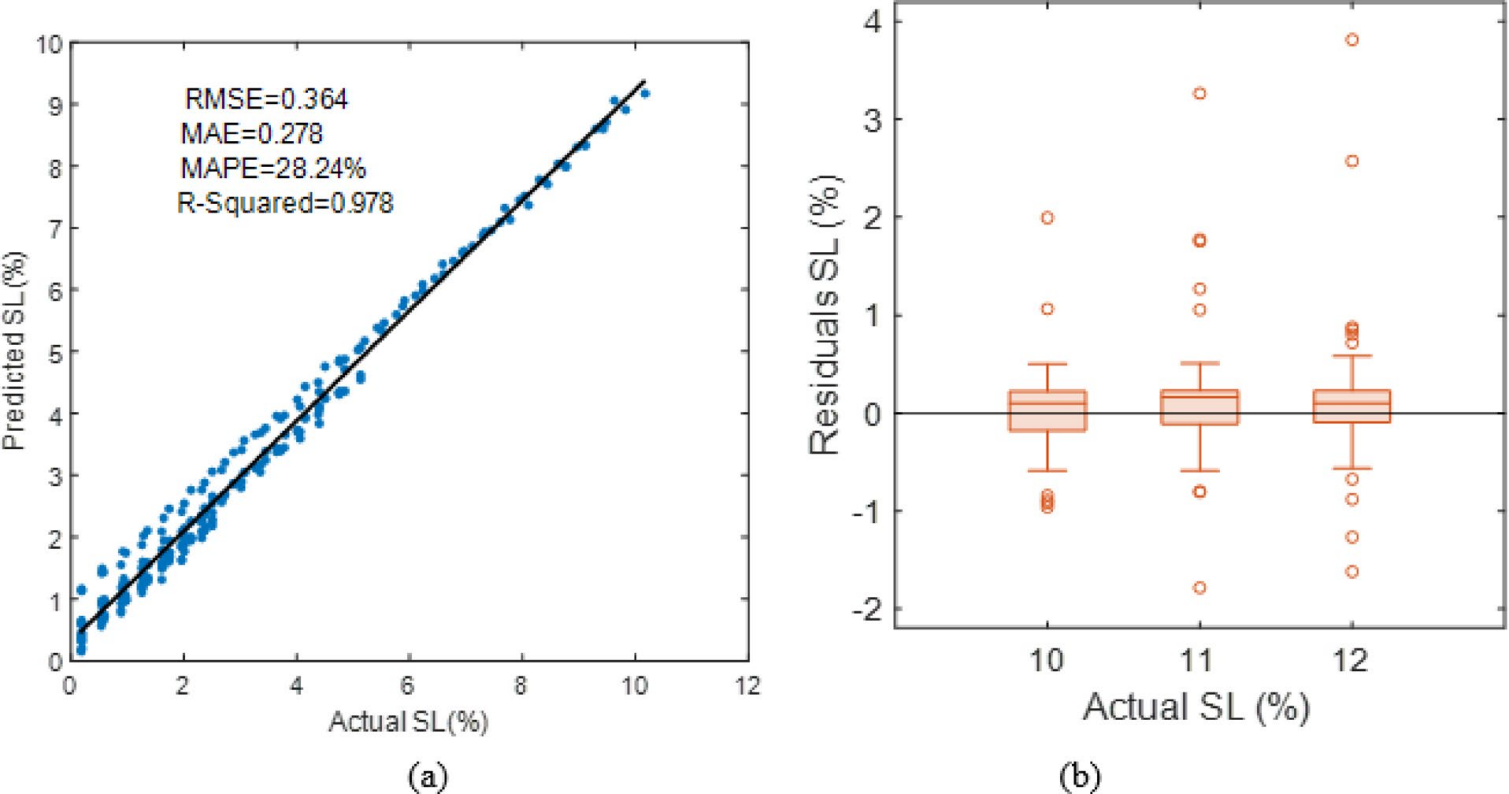
Overall empirical SL model plot of (**a**) R-squared (**b**) residual.

The empirical SL model’s performance is shown in Fig. 25. PV Panel (a) displays the regression plot between actual and expected soiling loss values. The data points closely correspond with the fitted regression line, indicating a high coefficient of determination (R2 = 0.978). The model’s low RMSE (0.364) and MAE (0.278) validate its trend capture. However, the mean absolute percentage error (MAPE) remains greater (28%), showing relative variances, especially for lower SL values. Panel (b) shows the residual distribution, where errors are centred around zero but include outliers. This residual spread shows systematic deviations not completely represented by the empirical formulation, motivating the upcoming section to use sophisticated machine-learning algorithms to minimize relative error while maintaining high R^2^.

Although the empirical SL model achieved a high coefficient of determination (R² ≈ 0.978), the MAPE value (~ 28%) appears relatively high. This is primarily due to the sensitivity of MAPE to small denominator values. Since several SL observations fall within low ranges (below 2%), even small absolute deviations lead to inflated percentage errors. Furthermore, the linear regression framework may not fully capture nonlinear dust accumulation patterns, contributing to structural bias at low SL levels. This constraint led to the introduction of machine learning algorithms to make percentage-based predictions more accurate. .

### Performance analysis of ML model

Experimental data was used to develop the machine learning model. The model inputs are AT, GHI, RH, WS, and CF, while the target variable is solar PV module SL. The prediction data size was 5 × 336 and divided 80:20 for training and testing, as shown in Table 7. SL is predicted using stacking, ANN, SVM, DT, and KNN models.

**Table 7:** Experimental feature description.

Feature	Type	Mean	Min – Max	Description
Cleaning	categorical	-	-	Hot-encoding utilized Daily [1000] Weekly [0100] Bimonthly [0010] Monthly [0001]
Temperature (°C)	Float	27.08	12.66–41.32	Ambient air temperature
Humidity (%)	Float	38.24	15.79–81.33	Relative humidity
GHI (W/m²)	Float	497.34	282.93–660.06	Global Horizontal Irradiance
Wind Speed (m/s)	Float	2.33	1.02–5.64	Wind speed affecting panel cooling
SL (%) (Target variable)	Float	2.22	0.0–10.17	Soiling loss due to dust accumulation

Statistical metrics are needed to assess machine learning models’ prediction performance for reliability and robustness. This research evaluated solar panel soiling loss models using MAE, RMSE, MAPE, and R2. These measures show the models’ capacity to reduce prediction errors, capture data variability, and generalize across environmental conditions.

#### Stack model performance evaluation

**Fig. 26:**
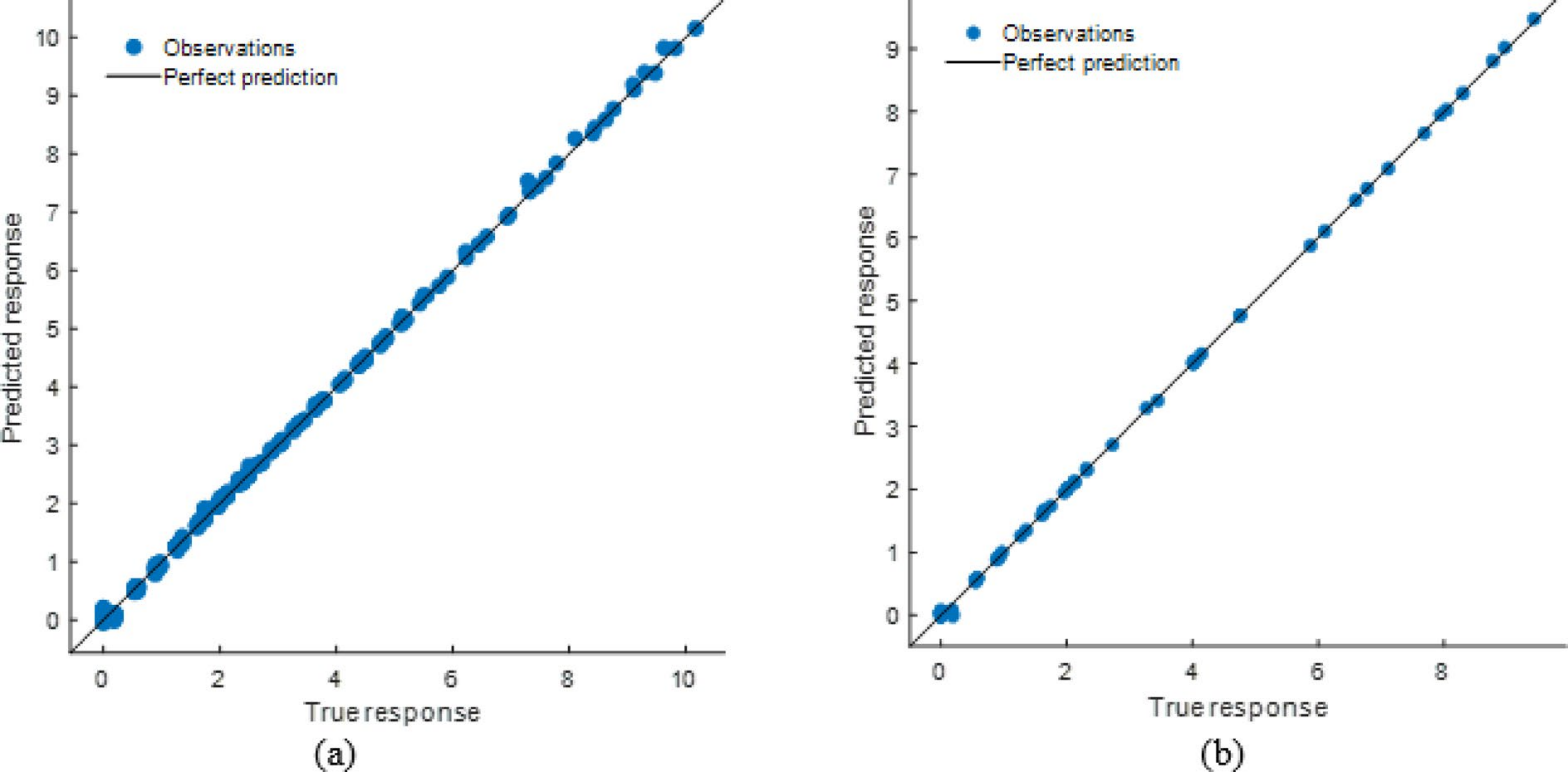
R^2^ plot of (**a**) Training (**b**) Testing data set.

**Fig. 27:**
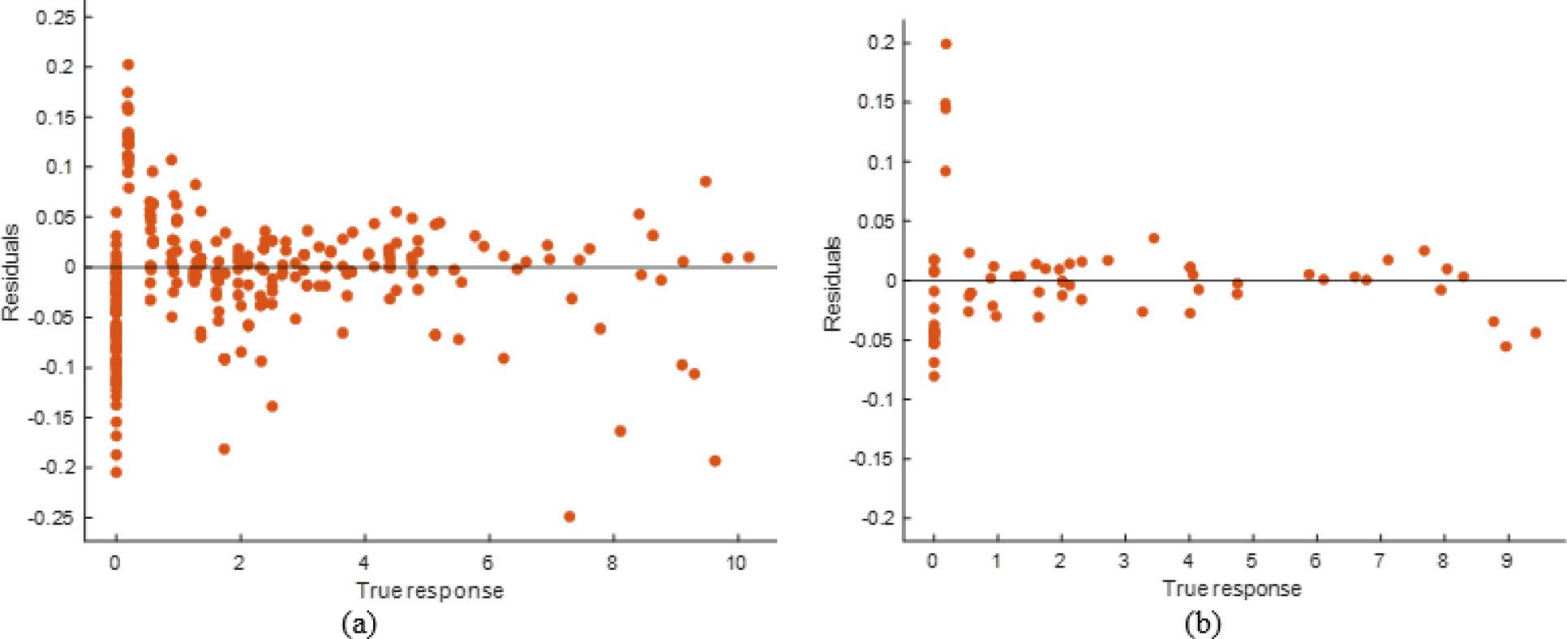
Residual plot of (**a**) Training (**b**) Testing data set.

The stacking ensemble model’s tight alignment of projected and observed responses in training and testing datasets showed high predictive performance in Fig. 26. The Fig. 27 shows residual plots with random residuals around zero, confirming the model’s dependability and lack of systematic bias. Performance metrics as shown in Table 8, which demonstrated the model’s resilience, with R² values of 0.9995 (training) and 0.9997 (testing) and low error values (RMSE: 0.0566 and 0.0456; MAE: 0.0404 and 0.0333). The stacking model generalizes effectively across datasets and outperforms individual models, making it a very accurate soiling loss prediction framework.

**Table 8:** Performance matrices of stacking model.

	Training	Testing
RMSE	0.056585	0.045594
MSE	0.003202	0.002079
R^2^	0.999474	0.999714
MAE	0.040445	0.033349

To examine whether cleaning frequency (CF) dominates the learning process, a feature ablation study was conducted by evaluating models trained using (i) the full feature set, (ii) CF alone, and (iii) environmental variables alone.

The full model consistently achieved the lowest prediction error. Although CF-only models exhibit strong correlation with soiling loss due to their causal relationship, they produce significantly higher absolute errors compared to the full model. Conversely, models trained exclusively on environmental variables perform poorly. Table 9 shows that environmental characteristics give important extra information and that cleaning frequency does not hide environmental learning.

**Table 9:** Feature ablation analysis of the stacking model.

Model	RMSE	MAE	MSE
Full model (CF + Environmental)	0.067003	0.041596	0.0044894
CF only model	0.18236	0.12861	0.033254
Environmental only model	3.3304	2.5385	11.091

#### ANN model performance evaluation

**Fig. 28:**
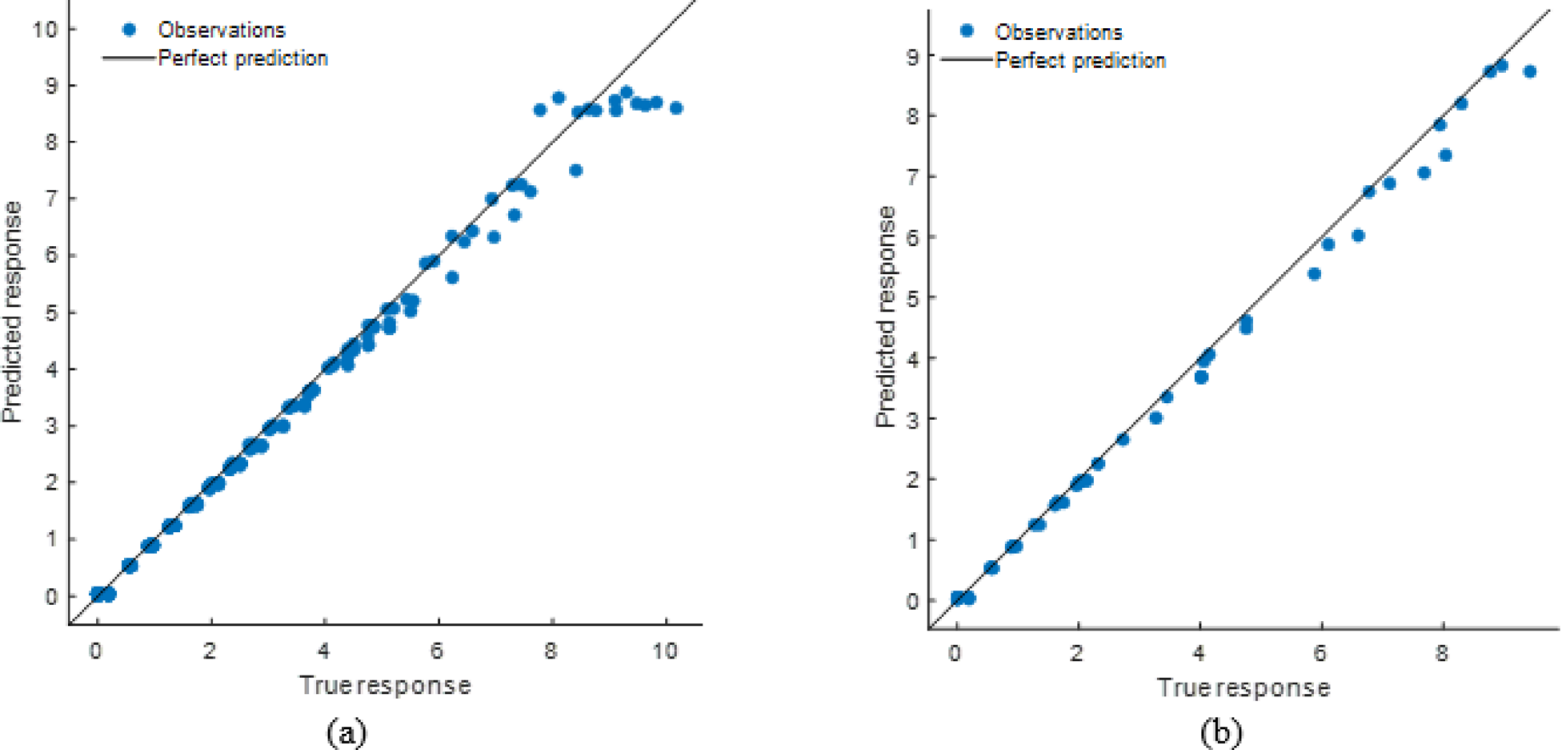
R^2^ plot of (**a**) Training (**b**) Testing data set.

**Fig. 29:**
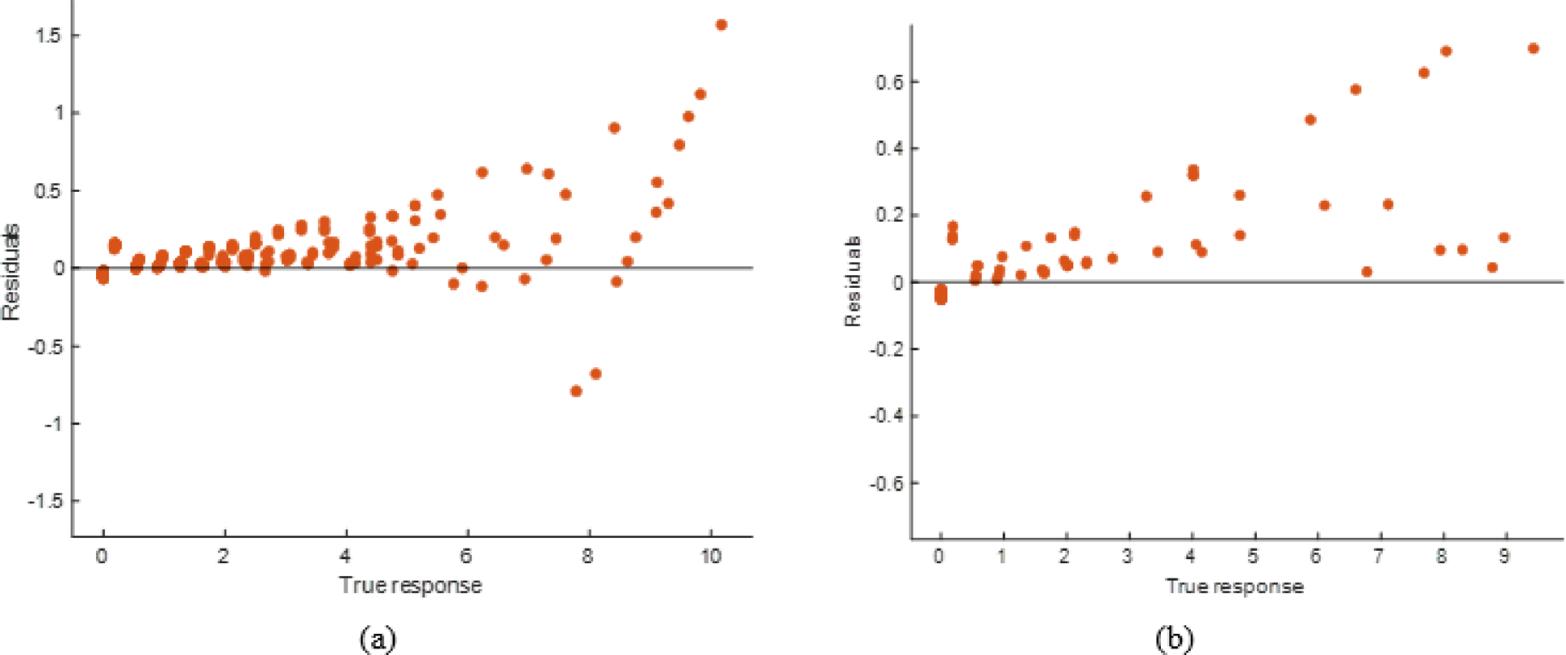
Residual plot of (**a**) Training (**b**) Testing data set.

The scatter plots reveal that the ANN model accurately predicted soiling loss as shown in Fig. 28. The stacking model had somewhat less departures from the ideal prediction line than the ANN, especially at higher response levels. The Fig. 29 residual plots reflect this tendency, with residuals spreading more broadly and displaying patterns at extreme values, indicating small bias in specific ranges. Performance measurements is shown in Table 10, which supports this result, with R² values of 0.9923 (training) and 0.9806 (testing) and greater error levels (RMSE: 0.2138 and 0.2822; MAE: 0.1277 and 0.1358). The ANN model is highly predictive, but its error distribution and somewhat lower accuracy than the stacking model suggest it cannot completely capture nonlinear data variability.

**Table 10:** Performance matrices of ANN model.

	Training	Testing
RMSE	0.217388	0.282226
MSE	0.047258	0.079651
R^2^	0.992233	0.989024
MAE	0.122768	0.135793

#### Decision tree model performance evaluation

**Fig. 30:**
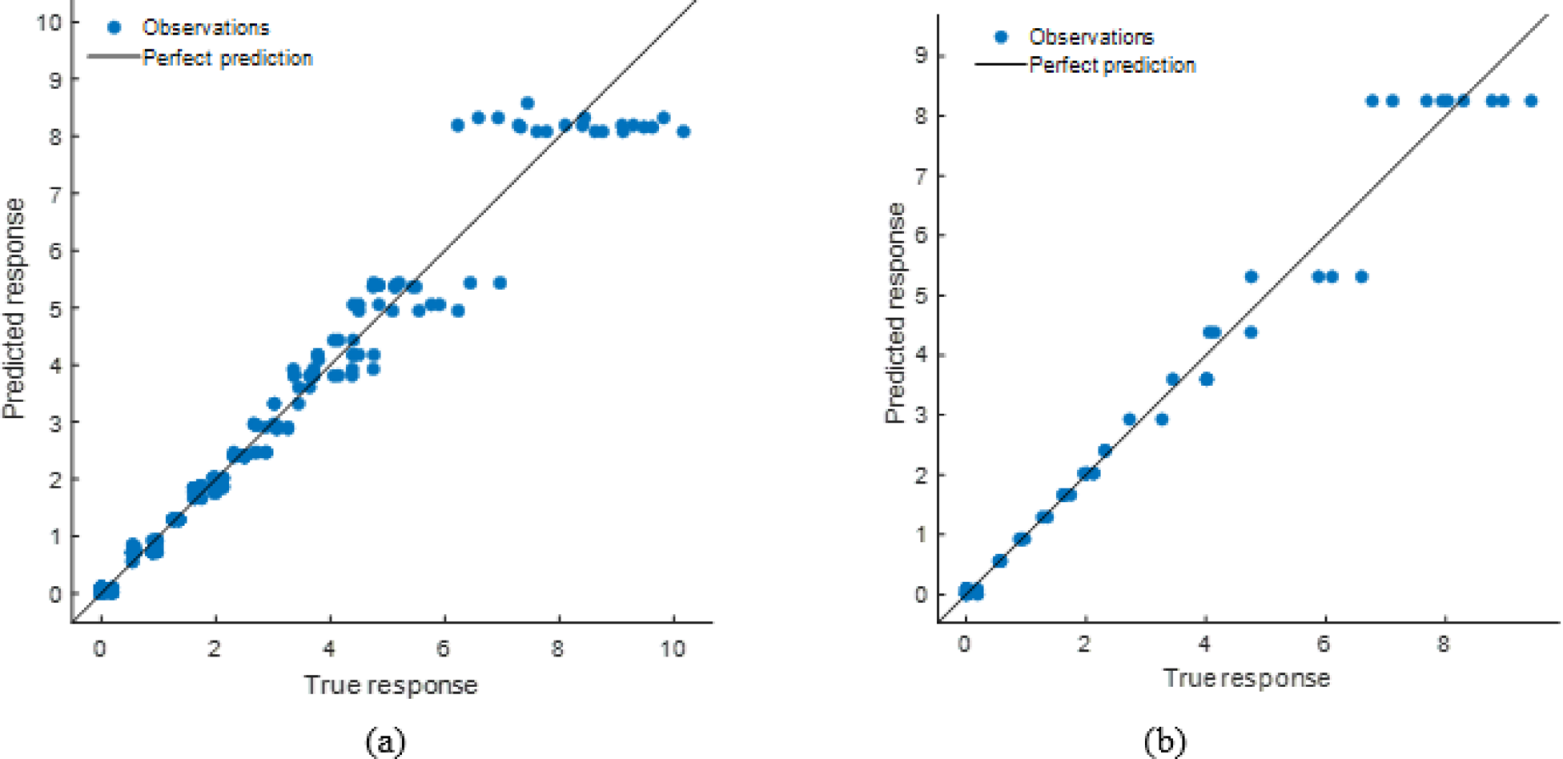
R^2^ plot of (**a**) Training (**b**) Testing data set.

**Fig. 31:**
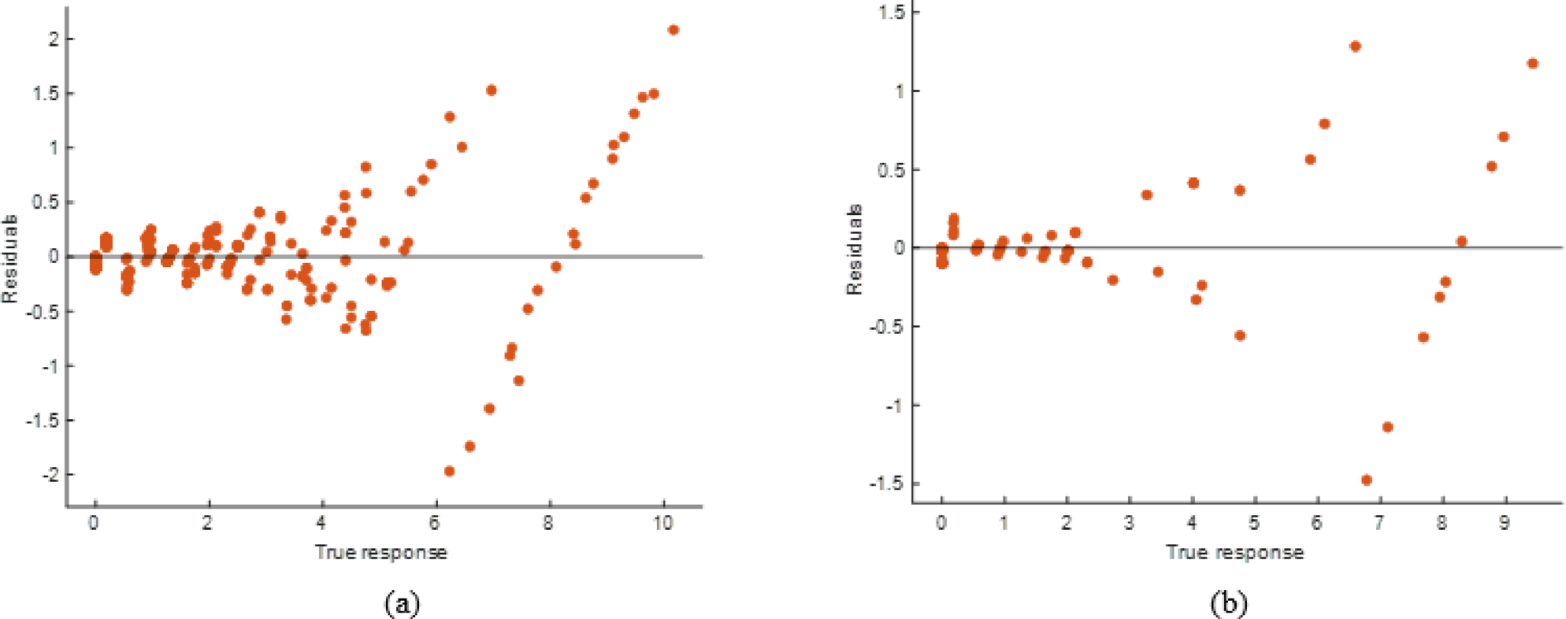
Residual plot of (**a**) Training (**b**) Testing data set.

Compared to the ANN and stacking models, the DT model predicted well but had lesser accuracy. The scatter plot (Fig. 30) demonstrates that although projected responses track the actual values, deviations from the ideal prediction line are greater at higher response levels. The Fig. 31 shows residual plots with larger dispersion and predictable patterns, showing overfitting in specific areas. This is supported by performance measurements (Table 11), including R² values of 0.9767 (training) and 0.9777 (testing), and higher error levels (RMSE: 0.3766 and 0.4020; MAE: 0.1989 and 0.2090). The DT model captures the input-soiling loss connection, but its restricted generalization and higher residual spread make it less suitable than sophisticated ensemble approaches.

**Table 11:** DT model performance matrices.

	Training	Testing
RMSE	0.376604	0.402039
MSE	0.141831	0.161635
R^2^	0.976689	0.977727
MAE	0.198889	0.209042

#### SVM model performance evaluation

**Fig. 32:**
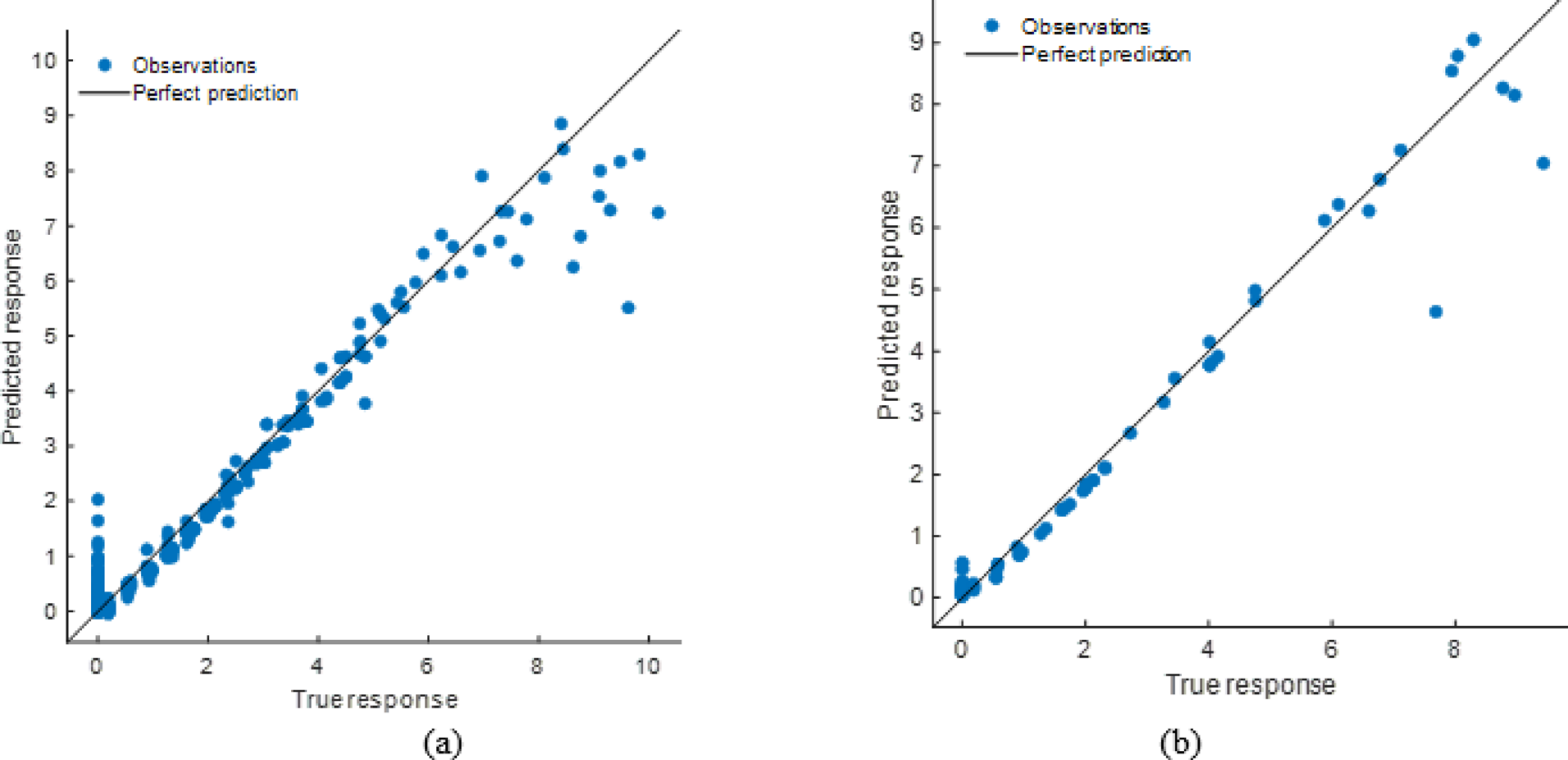
R^2^ plot of (**a**) Training (**b**) Testing data set.

**Fig. 33:**
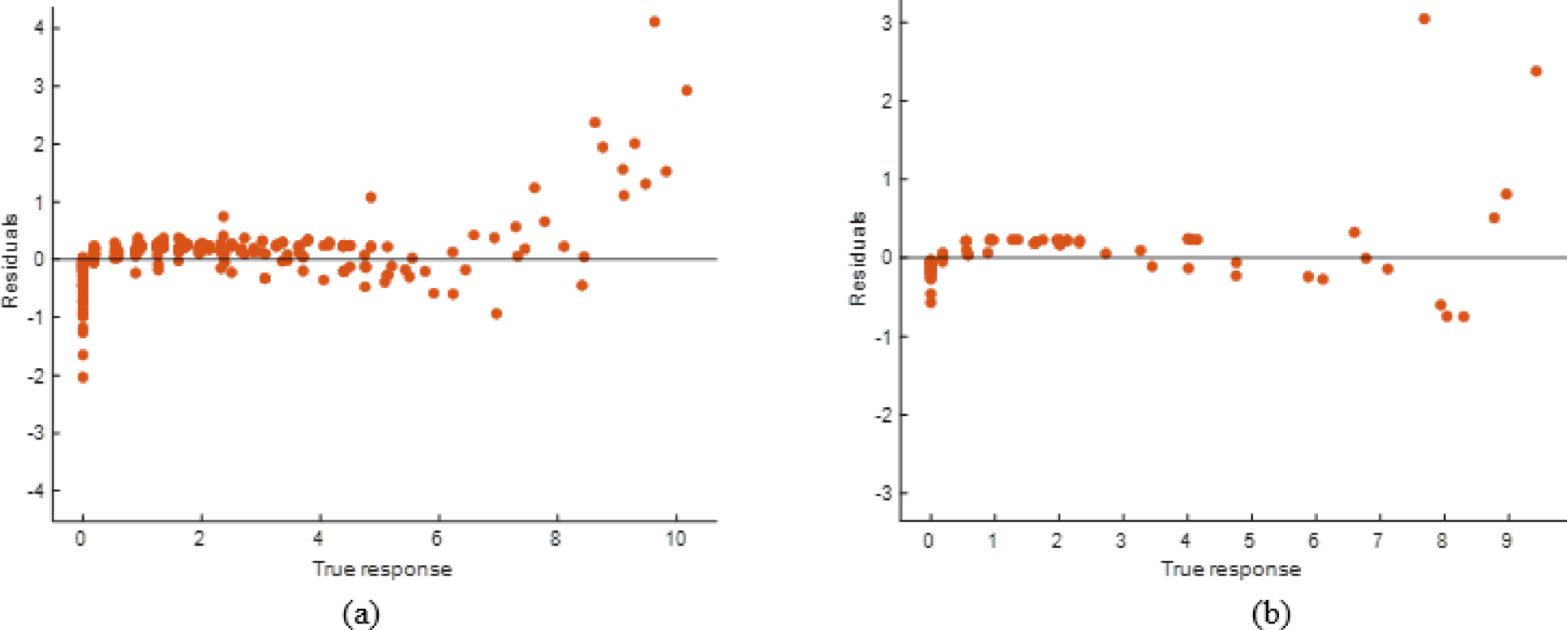
Residual plot of (**a**) Training (**b**) Testing data set.

The scatter plots are shown in Fig. 32 to show that the Support Vector Machine (SVM) model predicted values that matched observed responses. Significant departures from the ideal prediction line, especially at higher response levels, imply limits in catching extreme instances. The Fig. 33 residual plots show hetero-scedasticity in predictions, with errors spreading further at higher response levels. Performance measures (Table 12) indicate lower R² values (0.9527) and greater error values (RMSE: 0.5365 and 0.4418; MAE: 0.3124 and 0.2917) compared to ANN and stacking. SVM has superior generalization and testing performance than DT, but its lower accuracy and higher residual spread restrict it compared to the stacking ensemble.

**Table 12:** SVM Model performance matrices.

	Training	Testing
RMSE	0.536521	0.441826
MSE	0.287855	0.195211
R^2^	0.952689	0.973101
MAE	0.312405	0.291749

#### KNN model performance evaluation

**Fig. 34:**
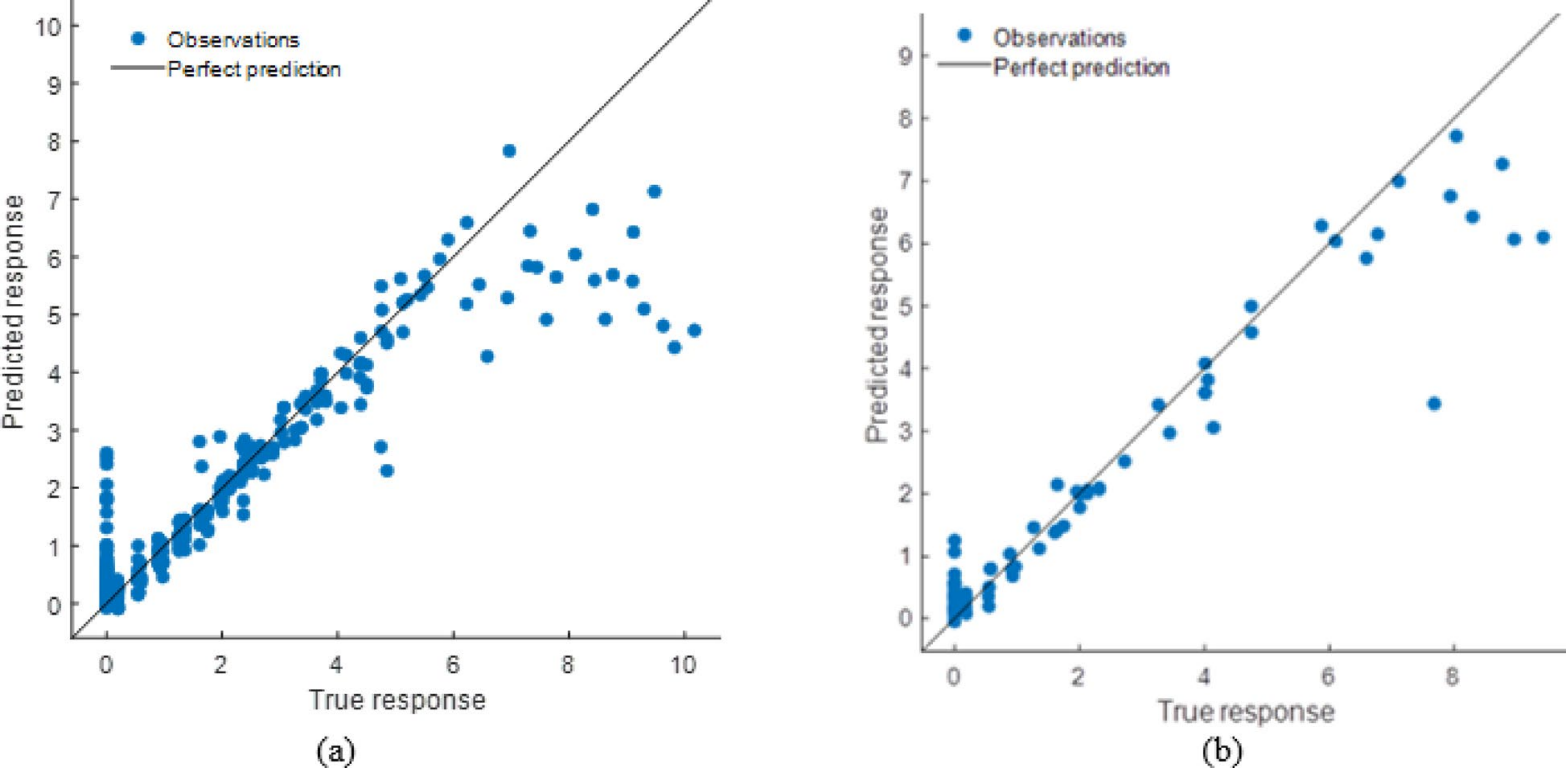
R^2^ plot of (**a**) Training (**b**) Testing data set.

**Fig. 35:**
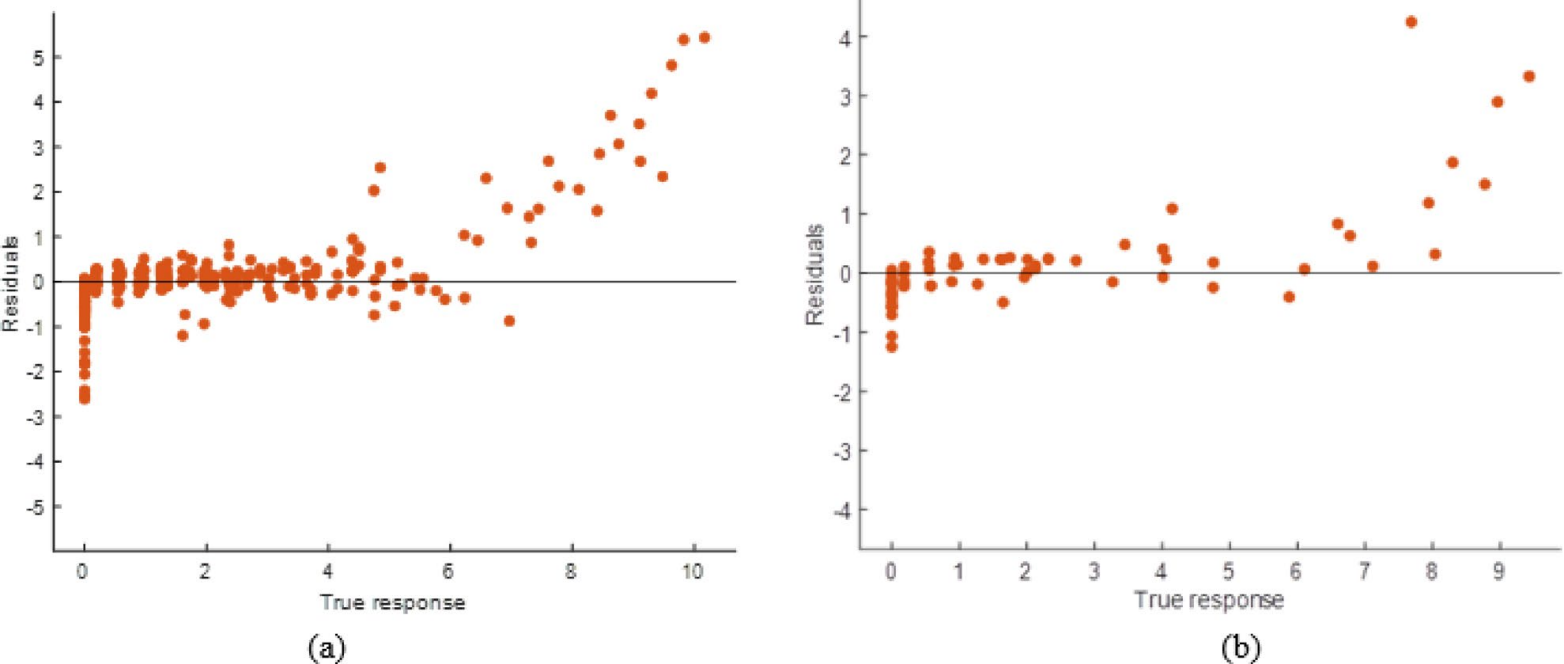
Residual plot of (**a**) Training (**b**) Testing data set.

As seen in the scatter plots (Fig. 34), the k-Nearest Neighbor (kNN) model had mixed predictive performance, with projected values following the genuine responses but deviating at higher response levels. The Fig. 35 residual plots show higher error dispersion and systematic bias in extreme ranges, indicating model resilience is lowered. Performance measures (Table 13) demonstrate high generalization on test set but poor fit during training, with R² values of 0.7662 (training) and 0.9701 (testing). Our error measurements were greater, with RMSE values of 1.1926 (training) and 0.4657 (testing) and MAE values of 0.6948 and 0.3904. Despite good testing accuracy, the kNN model’s large training error and residual spread overfit local patterns and impair dependability compared to stacking.

**Table 13:** KNN model performance matrices.

	Training	Testing
RMSE	1.192642	0.465708
MSE	1.422394	0.216884
R^2^	0.766219	0.970114
MAE	0.694797	0.390428

The slightly higher testing R² compared to training R² for the KNN model is attributed to the local interpolation nature of KNN and the distribution of samples in feature space, rather than data leakage. Since testing samples fall within well-represented regions of the training feature space, stable prediction performance is achieved.

#### Learning curve analysis for overfitting assessment

To assess potential overfitting and validate the generalization capability of the proposed stacking ensemble model, learning curve analysis is performed in accordance with statistical learning theory. The training and validation errors were evaluated as a function of increasing training data size. The learning curves demonstrate that although the training error decreases with increasing sample size, the validation error converges to a stable and closely aligned value without divergence. The narrow gap between training and validation errors confirms that the stacking model does not suffer from overfitting and generalizes well to unseen data.

The ensemble structure successfully balances the bias-variance trade-offs, so the validation error does not increase as the model capacity increases. These results provide theoretical and empirical evidence that the high R² values achieved by the stacking model are due to robust learning rather than memorization of the experimental dataset.

**Fig. 36:**
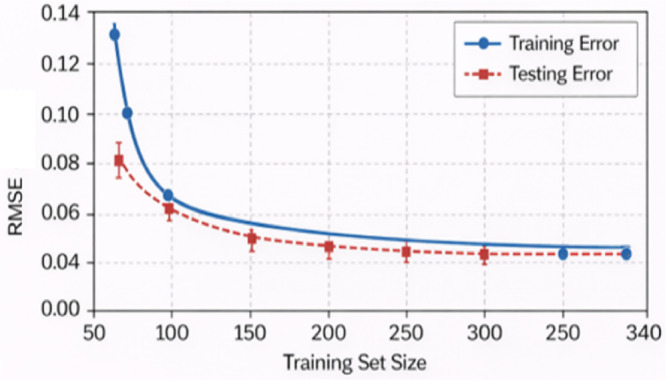
Learning curve for training and testing.

Also, the fact that the validation error doesn’t go up when the model capacity goes up shows that the ensemble structure does a good job of balancing bias and variation. Learning curves showing in Fig. 36 convergence of training and testing RMSE for the stacking ensemble, indicating strong generalization and absence of overfitting.

#### Noise sensitivity analysis

**Fig. 37:**
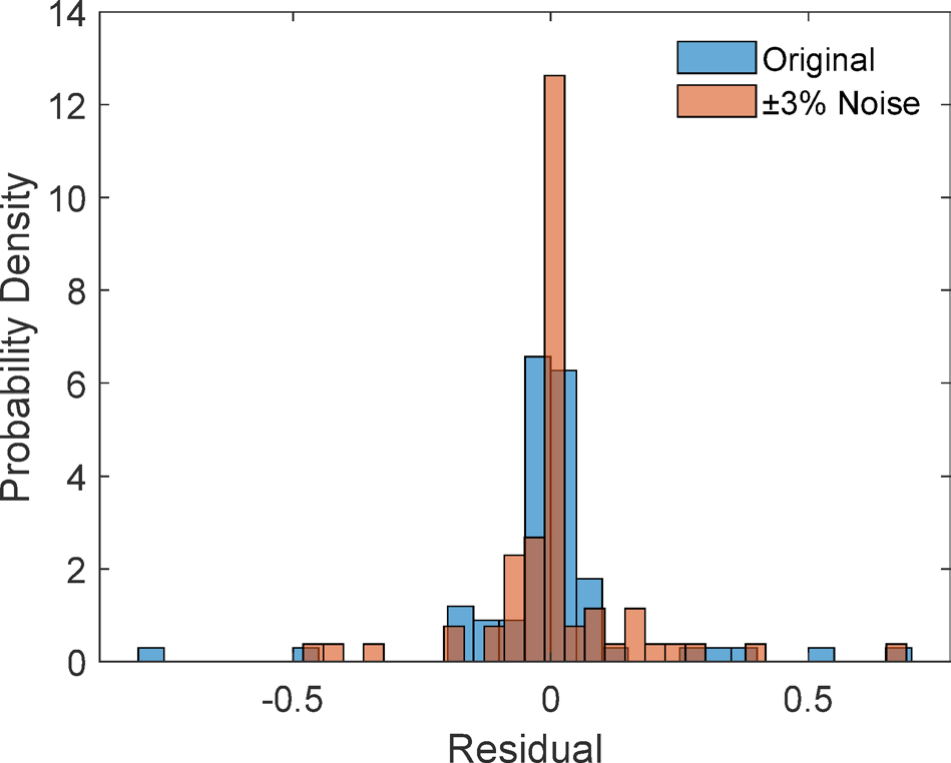
Residual distribution: original vs. noisy inputs (± 3%).

To assess the robustness of the stacking model against potential measurement noise, controlled Gaussian perturbations (± 3%) were introduced to environmental input variables. The model was retrained using the same train–test partition to ensure consistency. Figure 37 illustrates the residual distribution comparison between the original inputs and perturbed inputs.

### Comparative performance analysis

#### Cleaning interval wise analysis

**Fig. 38:**
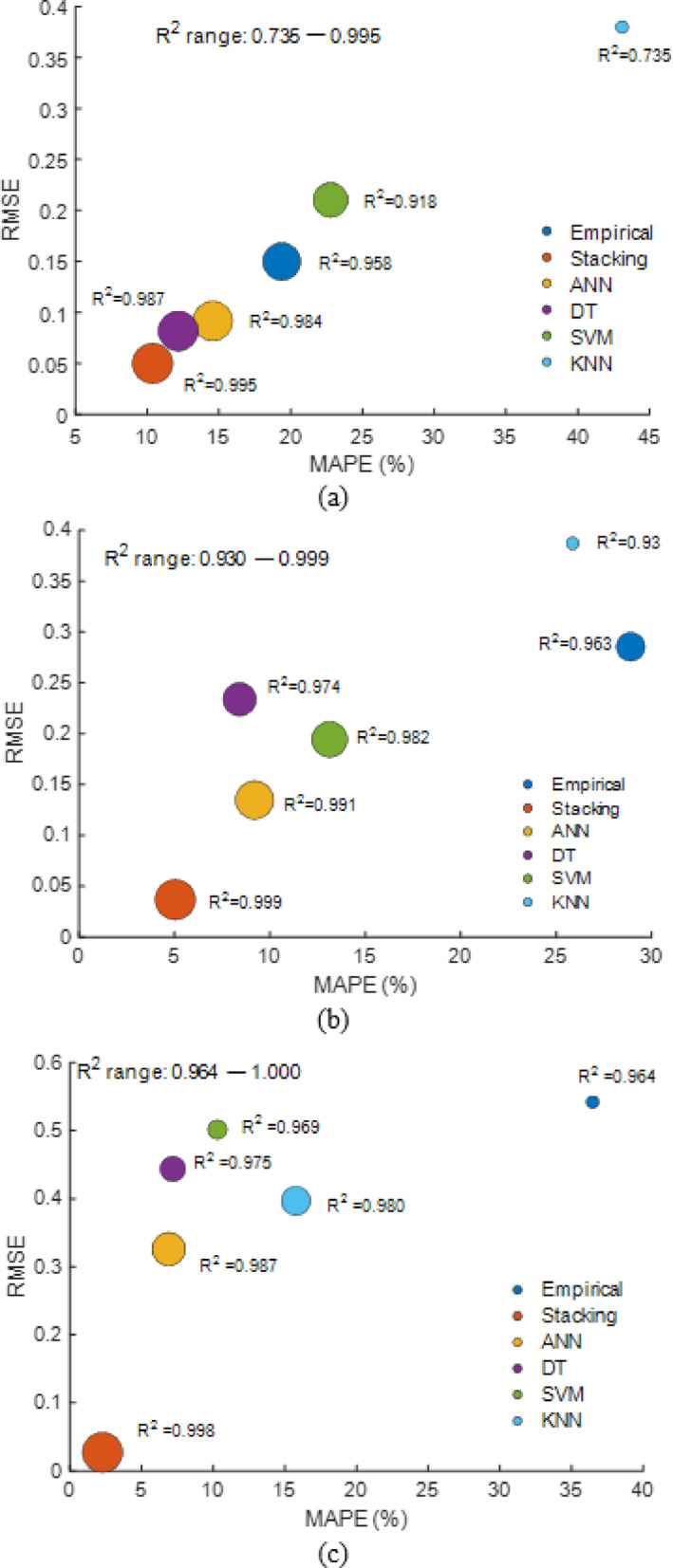
Model performance comparison (MAPE vs. RMSE, bubble ∝ R²) across cleaning frequency intervals (**a**) clean weekly (**b**) clean biweekly (**c**) clean monthly.

Model accuracy diminishes with longer cleaning intervals, with weekly cleaning reaching R² = 0.995 and low RMSE (between 0.05 and 0.1), whereas monthly cleaning drops R² to 0.964 and raises RMSE over 0.4, as shown in Fig. 38. In all intervals, the stacking model had the lowest error (e.g., MAPE < 10%, RMSE ≈ 0.05) and greatest R² (> 0.99), demonstrating its durability over individual models.

**Fig. 39:**
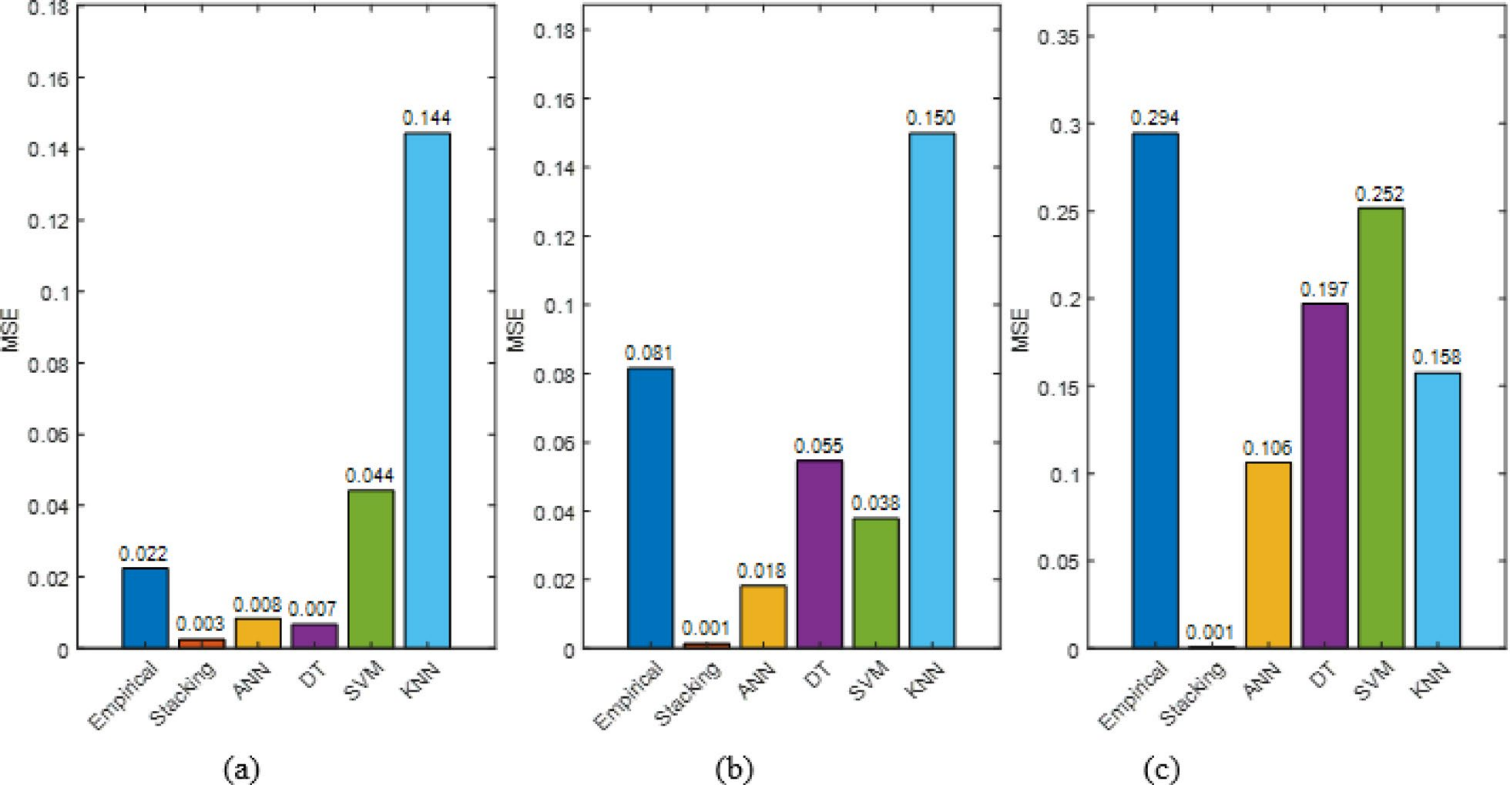
Model MSE comparison across cleaning-frequency intervals (**a**) clean weekly (**b**) clean biweekly (**c**) clean monthly.

Figure 39 demonstrates that stacking had the lowest MSE at all cleaning intervals: 0.003 (weekly), 0.001 (biweekly), and 0.001 (monthly). Empirical and KNN models had the largest errors, 0.022–0.294 and 0.144–0.158, respectively, especially during longer cleaning intervals. These findings confirm that stacking provides the most accurate and consistent forecasts regardless of cleaning frequency.

**Fig. 40:**
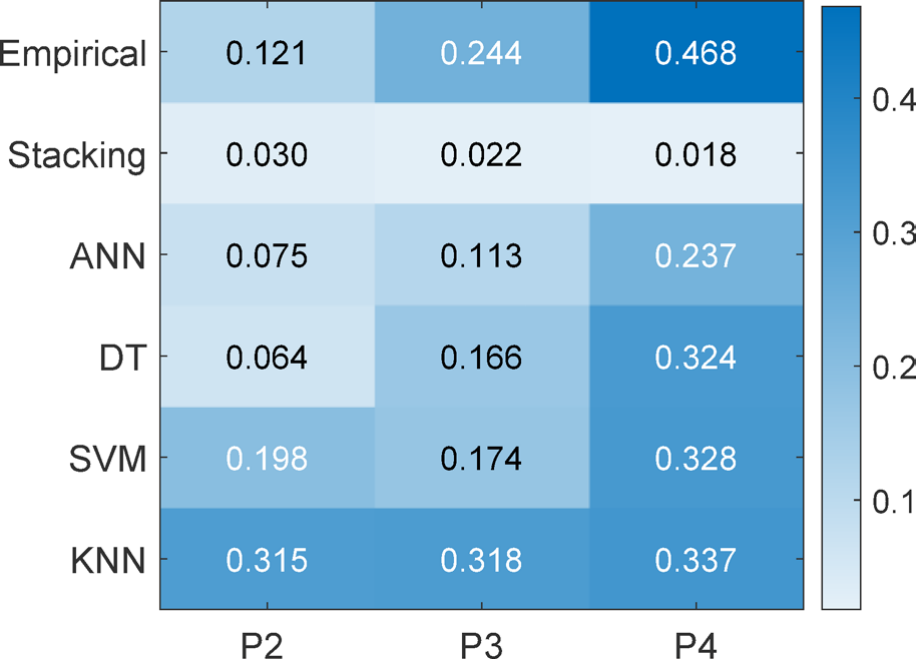
Model MAE Heatmap across cleaning intervals P2:- clean weekly P3:-clean biweekly P4:- clean monthly.

Figure 40 shows MAE fluctuation by cleaning interval. The stacking model had the lowest MAE values (0.030 (weekly), 0.022 (biweekly), and 0.018 (monthly), whereas empirical and KNN models had the largest errors (0.468 and 0.337, respectively). This proves stacking’s prediction error-reducing ability under protracted soiling.

These findings show that stacking is the best accurate method for soiling loss estimate across cleaning frequencies and is resilient to increasing soiling buildup.

#### Monthly performance analysis

Table 14 indicates that stacking consistently outperformed other models with RMSE ranging from 0.03 to 0.045, MAE ≤ 0.03, and R² = 0.999 throughout all months. Empirical and KNN models had the largest errors (e.g., MAPE up to 35.38% and MAE > 0.4), especially in December, proving the stacking ensemble’s better resilience and dependability.

**Table 14:** Month-wise performance metrics of predictive models.

Month	Model	RMSE	MSE	MAE	MAPE	*R* $\:{\boldsymbol{R}}^{2}$
October	ANN	0.131	0.017	0.092	8.576	0.996
	DT	0.279	0.078	0.181	9.237	0.986
	Empirical	0.356	0.127	0.272	26.601	0.977
	KNN	0.307	0.094	0.267	30.92	0.983
	SVM	0.238	0.056	0.192	13.277	0.989
	Stacking	0.04	0.001	0.024	6.239	0.999
November	ANN	0.3	0.09	0.224	13.063	0.986
	DT	0.33	0.109	0.204	10.232	0.983
	Empirical	0.386	0.149	0.294	27.002	0.977
	KNN	0.346	0.12	0.289	18.403	0.981
	SVM	0.38	0.144	0.269	16.354	0.977
	Stacking	0.045	0.002	0.027	6.741	0.999
December	ANN	0.158	0.025	0.106	9.014	0.995
	DT	0.265	0.07	0.166	8.283	0.988
	Empirical	0.348	0.121	0.266	31.13	0.979
	KNN	0.486	0.236	0.411	35.38	0.959
	SVM	0.363	0.131	0.237	16.56	0.977
	Stacking	0.03	0	0.017	4.703	0.999

**Fig. 41:**
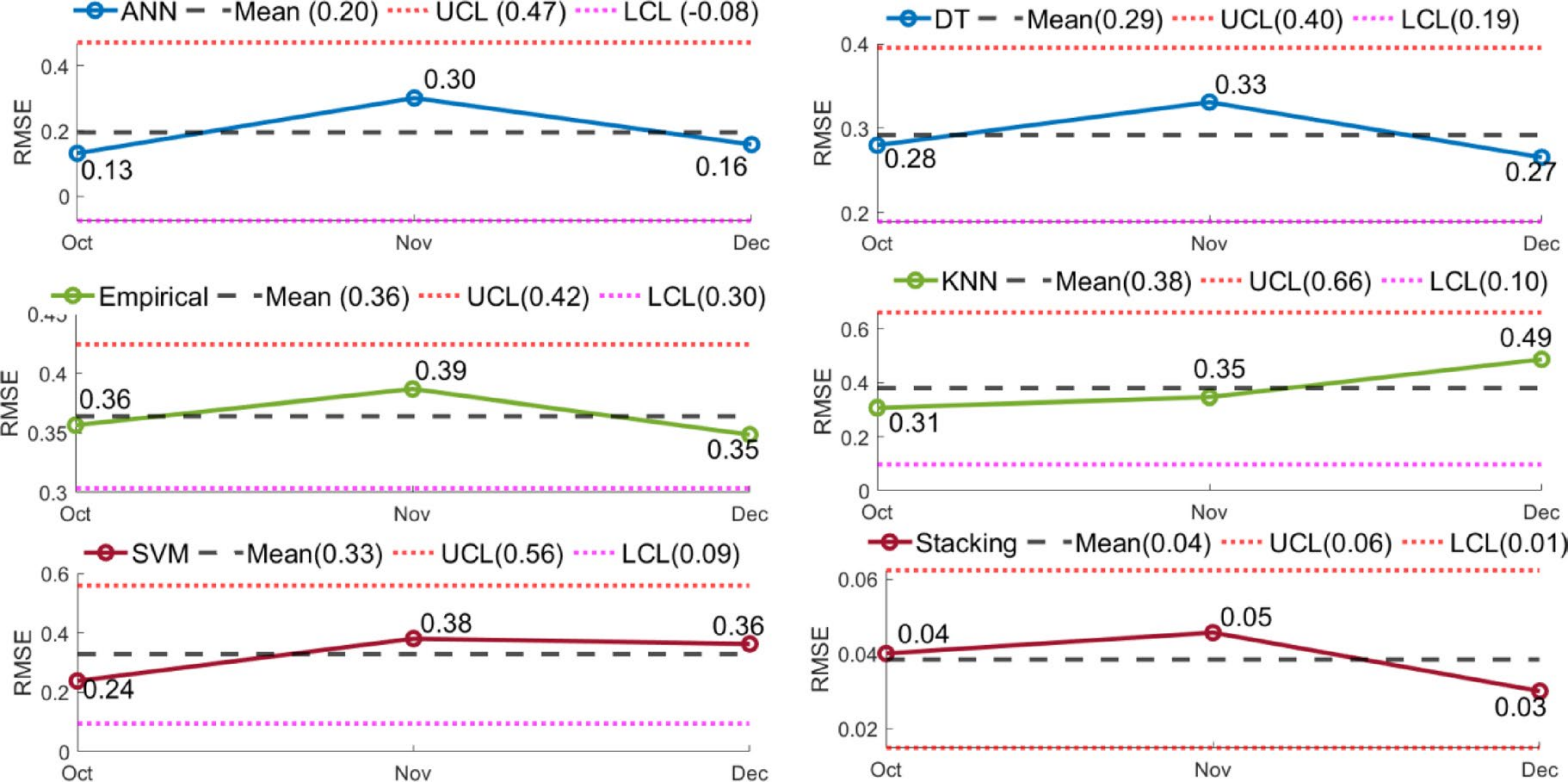
Shewhart control charts of RMSE for different predictive models (Oct–Dec 2024).

**Fig. 42:**
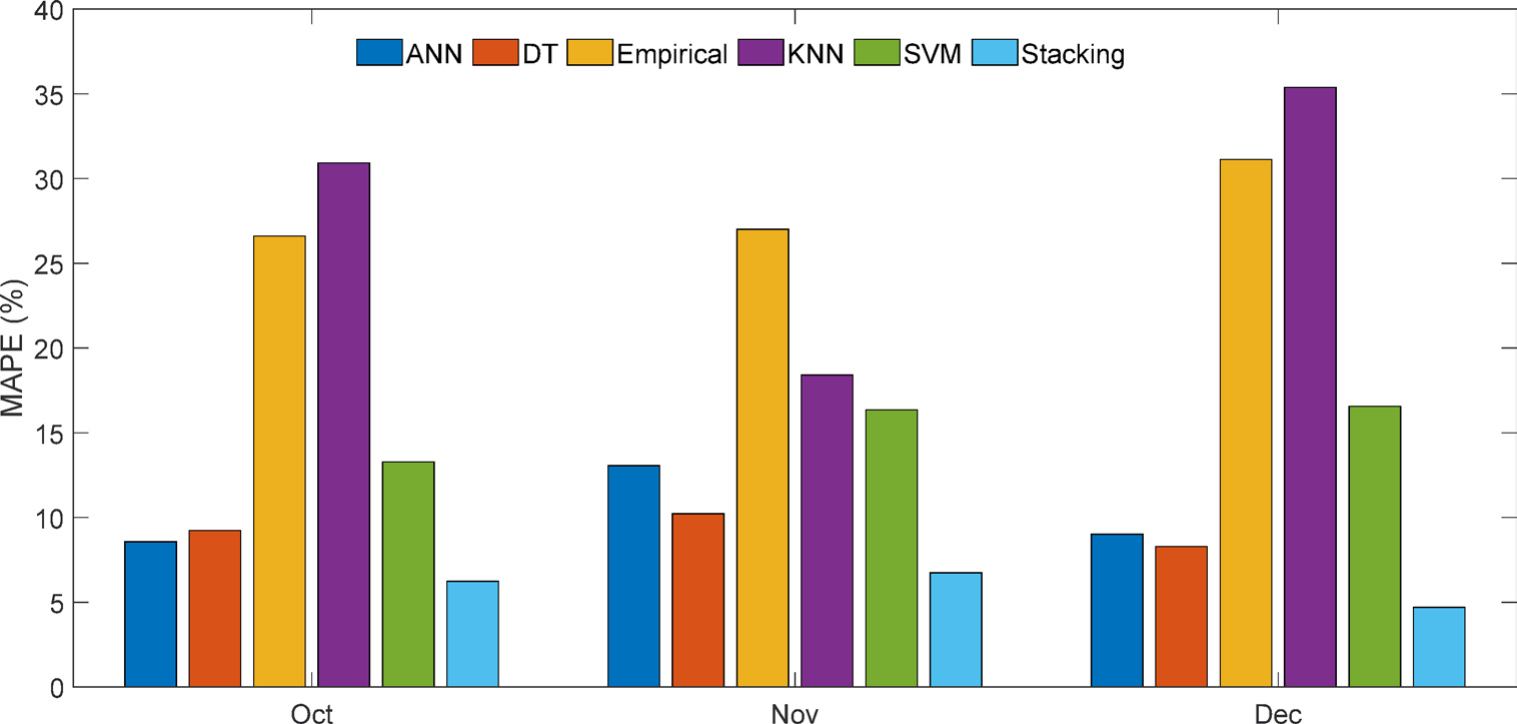
Month-wise comparison of MAPE (%) across predictive models.

**Fig. 43:**
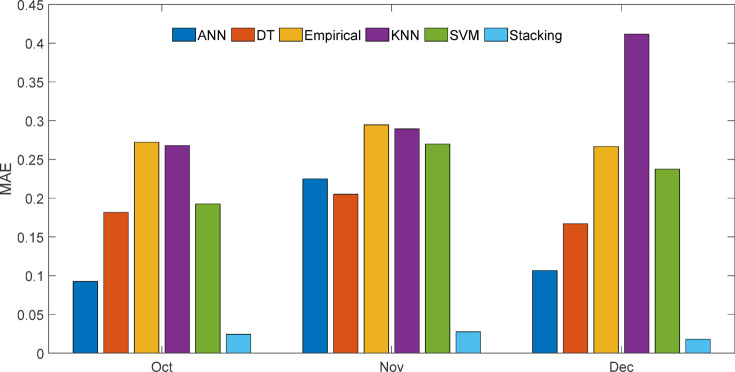
Month-wise comparison of MAE across predictive models.

Shewhart control charts of RMSE for October–December 2024 forecasting models are shown in Fig. 41. Control charts, or Shewhart charts, provide performance data over time to determine control limits. The upper and lower control limits (UCL and LCL) set the permitted range of variation. Values over these limits indicate instability or unexpected swings. Central line (CL) shows process mean. The stacking model showed the most consistent projected accuracy throughout all months, with an average RMSE of 0.04 and tight control limits (UCL = 0.06, LCL = 0.01). ANN showed RMSE variation between 0.13 and 0.30 (mean 0.20), DT between 0.27 and 0.33 (mean 0.29), and KNN peaked at 0.49 (mean 0.38), suggesting greater variability. The stacking ensemble predicts well because of its low errors (Figs. 42 and 43) and process stability.

The Shewhart control chart of RMSE (Fig. 41 shows that prediction errors remain well within the statistical control limits across all months, confirming stable model performance and absence of instability due to non-stationarity. The empirical regression model also maintained consistent performance, with R² values between 0.97 and 0.98 across different months, further supporting the temporal robustness of the predictive framework. The environmental variables recorded during the experimental period exhibited natural variability while remaining within the same physical operating regime, enabling the model to learn stable relationships between environmental drivers and soiling loss. Since the models rely on physically meaningful predictors such as cleaning frequency, humidity, and irradiance, the learned relationships remain consistent over time. These results confirm that the predictive models demonstrate stable performance across different months without evidence of significant parameter drift or non-stationary.

The Shewhart control chart analysis shows that the stacking model predicts soiling loss with the lowest prediction errors and retains stability within restricted limits, making it the most dependable strategy^63^.

To ensure that the superior performance of the stacking model was not merely a consequence of increased model flexibility, several safeguards were employed:

- Independent testing evaluation (80:20 split) demonstrated that training and testing R² values were nearly identical (0.9995 vs. 0.9997), indicating strong generalization without overfitting.
- Residual analysis showed random dispersion without systematic patterns.
- Month-wise and cleaning-frequency-wise evaluations confirmed consistent performance across operational conditions.
- Control chart stability analysis demonstrated low variance and stable error distribution across months.

These results collectively indicate that the improved performance of the stacking model arises from its ability to capture nonlinear environmental interactions rather than merely from increased complexity.

#### Comparison with semi-empirical baseline model

To evaluate the effectiveness of the proposed machine learning framework, its performance was compared with the semi-empirical regression model developed in Sect. 3.2 under identical validation conditions. The empirical model represents a physics-based baseline using environmental predictors such as irradiance, temperature, humidity, wind speed, and cleaning frequency. As shown in Table 12, the stacking ensemble achieved significantly lower prediction error (RMSE = 0.03–0.045, MAE ≤ 0.03) compared to the empirical model (RMSE = 0.348–0.386, MAE = 0.266–0.294). This represents an approximately 85–90% reduction in prediction error. These results demonstrate the superior predictive capability of the proposed machine learning framework in capturing nonlinear soiling dynamics compared to conventional semi-empirical models.

### Statistical analysis of models

After performance assessment, statistical analysis verified machine learning model dependability and robustness. Although error measurements like RMSE, MAE, MAPE, and R² assess accuracy, they do not adequately resolve bias between estimated and actual soiling loss levels. A Bland–Altman (BA) plot was utilized to visually evaluate agreement, highlight recurring deviations, and indicate prevalent prediction error boundaries. A non-parametric Wilcoxon signed-rank test was employed to see whether predicted and actual values differed significantly. These graphical and inferential methods analyse model performance comprehensively.

#### Temporal independence and autocorrelation analysis

**Fig. 44:**
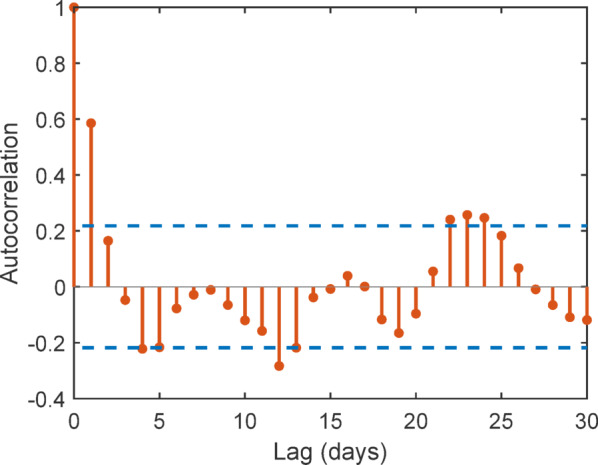
Autocorrelation function of daily-averaged soiling loss residuals.

To evaluate the statistical independence of the experimental observations, the autocorrelation function (ACF) of the soiling loss time series was analyzed, as shown in Fig. 44 Since environmental and PV performance data are collected sequentially, temporal autocorrelation may reduce the effective sample size and affect model validity^64^.

The ACF results show that autocorrelation values decrease rapidly and remain within the 95% confidence bounds for most lags. Only short-term correlations are observed at very small lags, while longer lags exhibit negligible autocorrelation. This indicates weak temporal dependence and confirms that the observations are sufficiently independent for predictive modelling.

Furthermore, the natural variability in environmental parameters, including irradiance, temperature, humidity, and wind speed, along with different cleaning frequencies, ensured diverse operating conditions across samples. This variability further supports the effective independence of observations and validates the robustness of the machine learning models.

#### Bland–Altman analysis

The Bland–Altman (BA) study assessed the agreement between anticipated and actual soiling loss values. The BA plot shows bias and limitations of agreement, indicating systematic and random model prediction deviations, unlike traditional error measures. A lower bias value and narrower ranges of agreement imply that model predictions match data. This approach helps validate if machine learning models can reproduce experimental observations across operational circumstances.

**Fig. 45:**
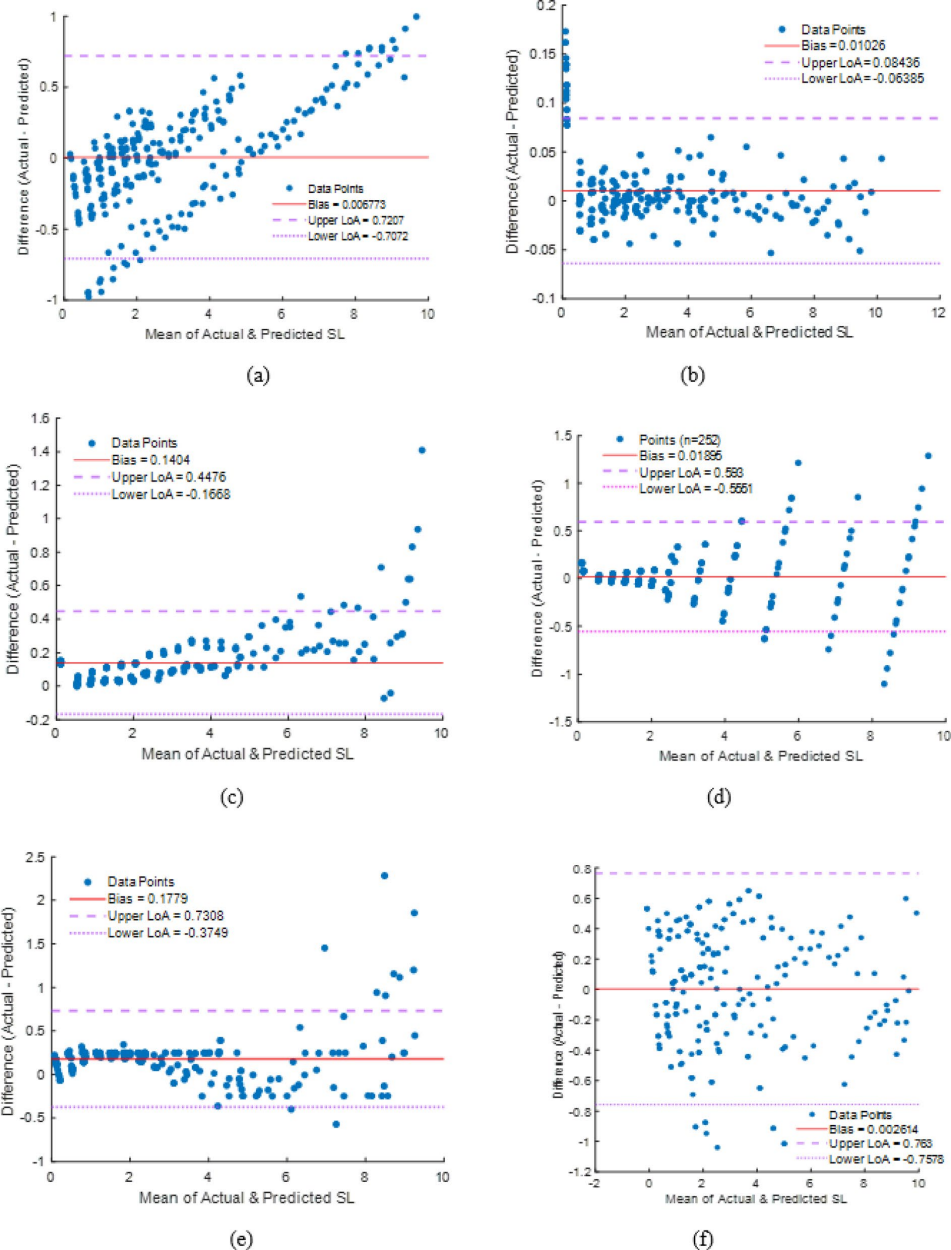
Bland–Altman plot for (**a**) Empirical model (**b**) Stacking ML model (**c**) ANN (**d**) DT (**e**) SVM (**f**) KNN.

**Fig. 46:**
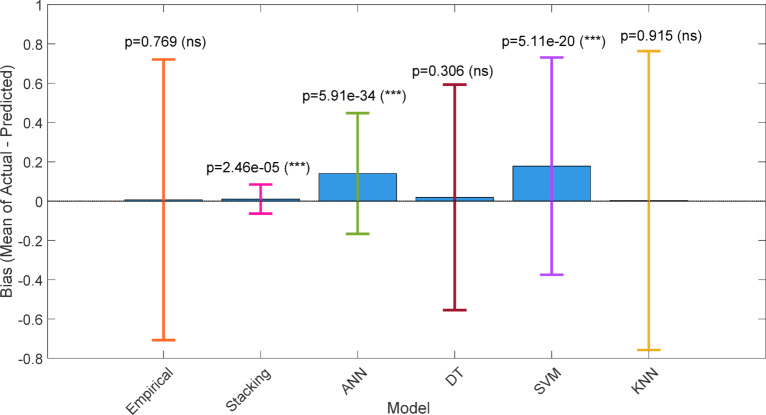
Model biases with limits of agreement (vertical lines).

The arrangement of dots around zero illustrates the degree of concordance between predictions and actual values, with tighter clustering near the red bias line signifying enhanced consistency. The dispersion within the limits of agreement (LoA) indicates the model’s variability, while outliers situated far beyond the LoA denote instances of inaccurate predictions, as depicted in Figs. 45 and 46, respectively.

.

#### Wilcoxon signed-rank test

**Fig. 47:**
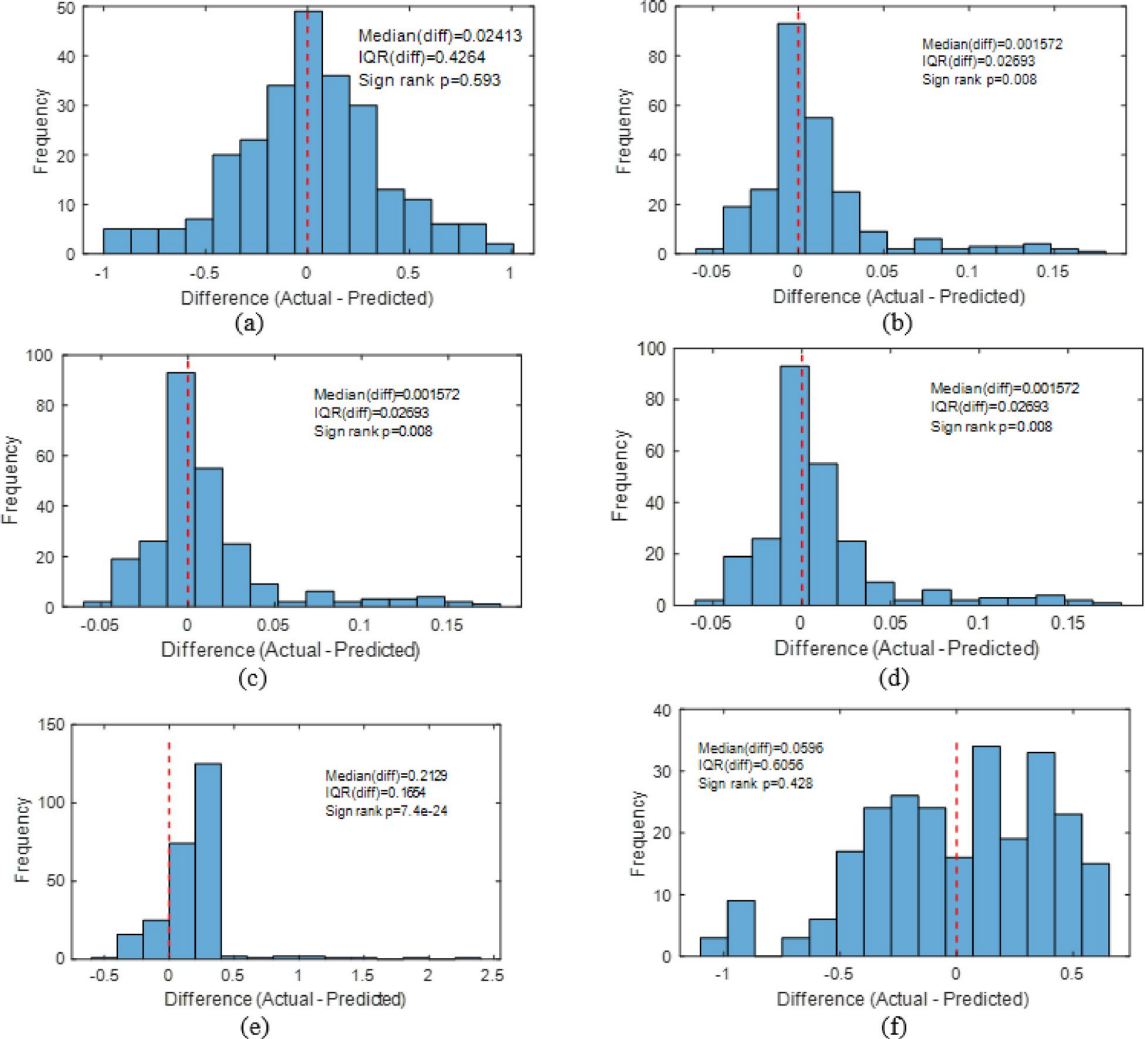
Wilcoxon signed-rank test plot for (**a**) Empirical model (**b**) Stacking ML model (**c**) ANN (**d**) DT (**e**) SVM (**f**) KNN.

The Fig. 47 shows the Wilcoxon signed-rank test comparing real and forecasted soiling loss values to assess the models’ predictive ability. A statistically insignificant result (*p* > 0.05) suggests that model predictions match experimental results, indicating model resilience. However, a significant finding (*p* < 0.05) indicates consistent disparities between projected and actual values. All models’ actual and expected soiling loss values were compared using the Wilcoxon signed-rank test. Despite having the lowest median difference (0.0016), the stacking model has a substantial p-value (*p* = 0.0083), demonstrating its capacity to capture tiny deviations with high consistency. The empirical (*p* = 0.593), decision tree (*p* = 0.276), and KNN (*p* = 0.428) models had non-significant p-values, indicating no statistically significant difference between their predictions and actual values.

Even with strong numerical performance, ANN (*p* = 2.71e-42) and SVM (*p* = 7.4e-24) showed substantial discrepancies, suggesting systematic prediction errors. Stacking is the most reliable technique since it has minimum bias and statistically significant consistency, whereas empirical and tree-based approaches are equivalent but less robust. Although the Wilcoxon signed-rank test yielded a statistically significant p-value (*p* < 0.01) for the stacking model, indicating that the median difference between predicted and actual values is not exactly zero, the magnitude of this deviation was extremely small (≈ 0.001–0.002). Given the relatively large sample size, even minor deviations can become statistically significant. However, absolute error metrics (RMSE and MAE) remained very low, suggesting that the detected bias is negligible in practical terms. Therefore, the stacking model demonstrates high predictive accuracy with minimal practical bias rather than perfect agreement.

Bland–Altman analysis and Wilcoxon signed-rank test p-values vary because they employ different statistical methods. The Bland–Altman approach estimates the p-value using a paired t-test to see whether the mean difference (bias) between actual and predicted values is substantially different from zero. The Wilcoxon signed-rank test, on the other hand, tests if the median of the paired differences deviates considerably from zero without assuming normality. BA focuses on systematic bias in the mean, whereas Wilcoxon confirms median differences, therefore p-values may vary. Two methods give a more complete statistical assessment of model performance.

#### Generalization scope and domain robustness analysis

**Fig. 48:**
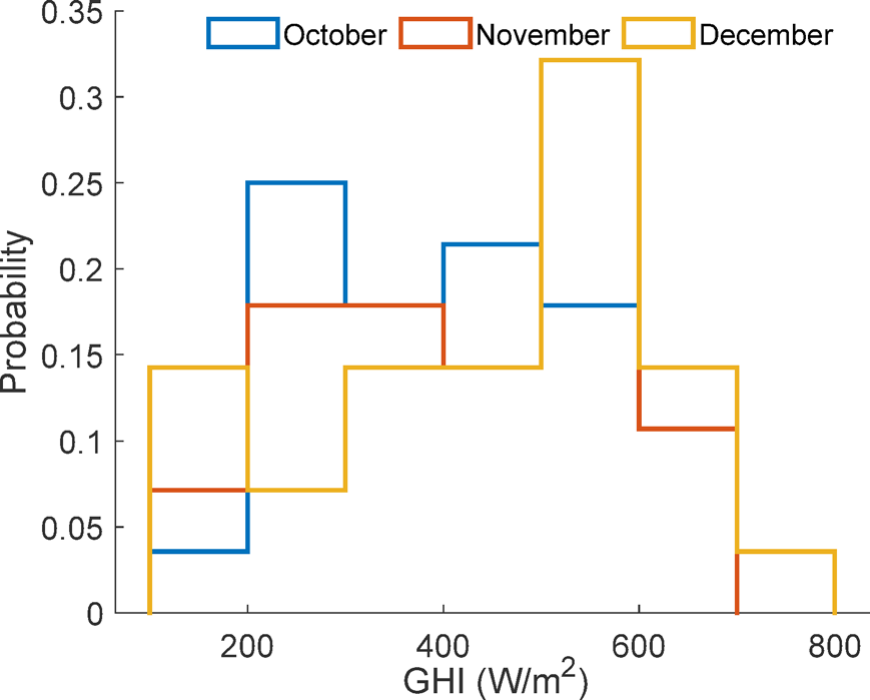
Covariate distribution shift of GHI across months.

Although the dataset originates from a single geographical site and season, meaningful domain shifts exist within the data due to temporal variation in environmental conditions and operational variation in cleaning frequency. Figure 48 illustrates covariate distribution shifts of global horizontal irradiance across months, confirming changes in the input feature space.

**Fig. 49:**
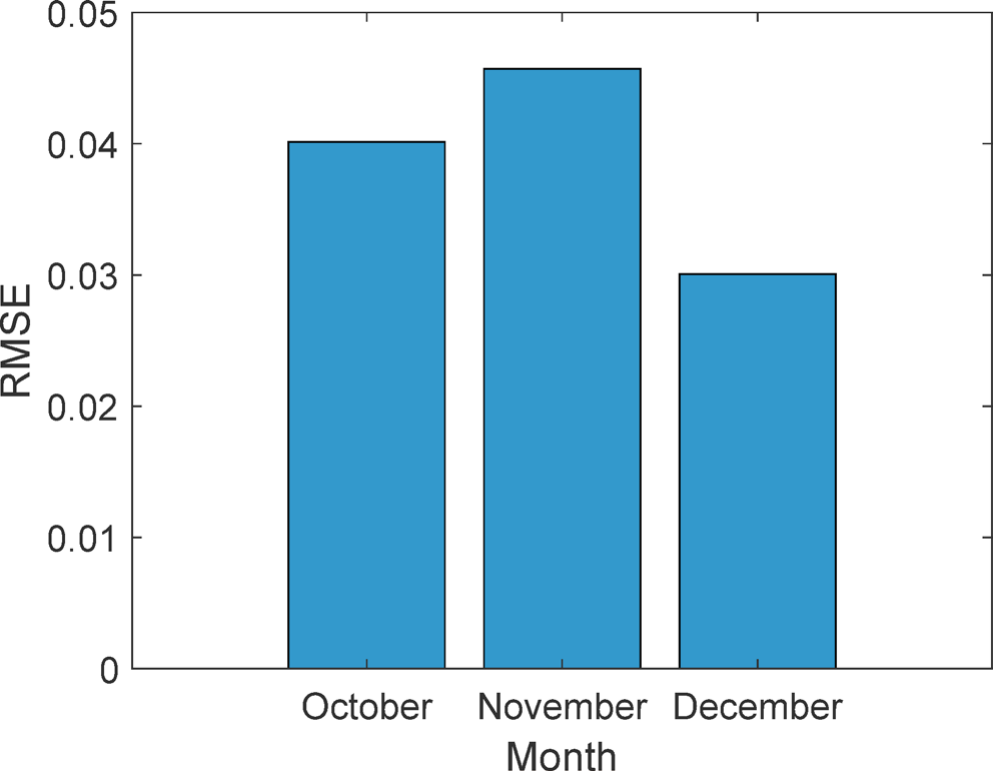
Temporal domain shift evaluation of stacking model.

**Fig. 50:**
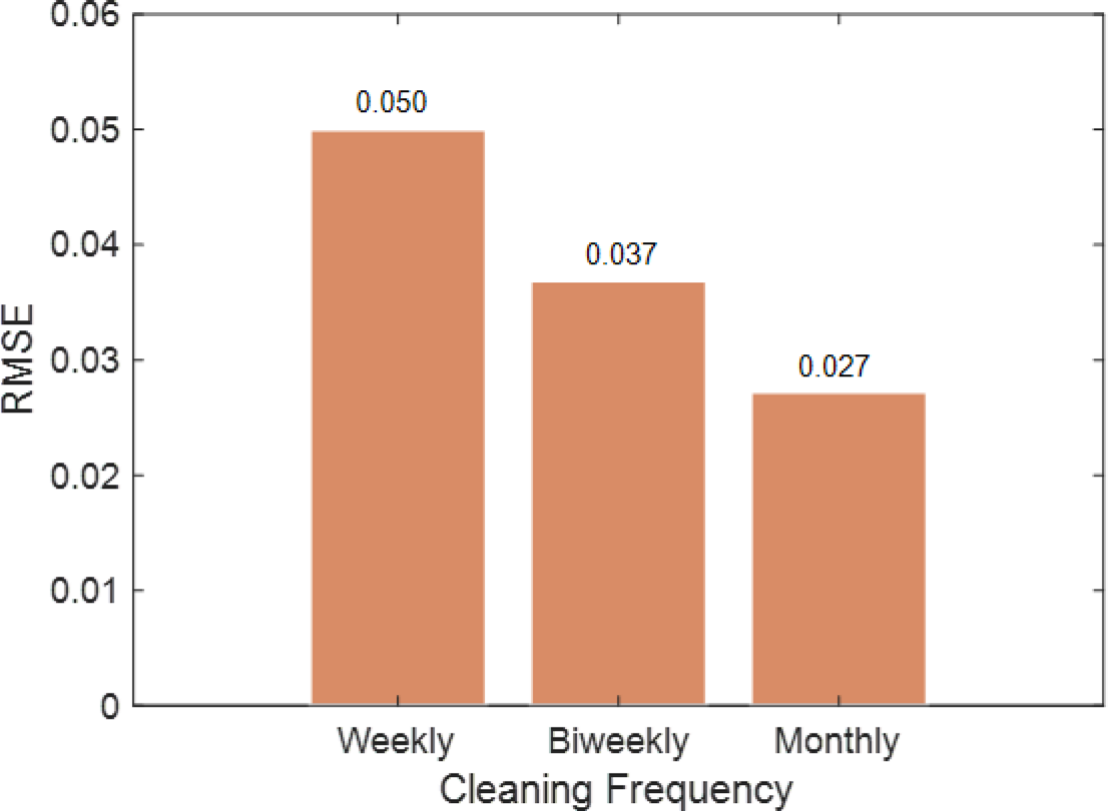
Operational domain shift evaluation of stacking model.

Figures 49 and 50 further demonstrate that the stacking model maintains stable RMSE across temporally distinct months and across different cleaning frequencies. The consistency of predictive performance under these distributional and operational shifts indicates robust within-domain generalization rather than simple interpolation of identical conditions. While the present study does not claim cross-climate transferability, the proposed framework demonstrates strong robustness within the studied domain.

#### Diebold–Mariano test results

To statistically validate the observed performance dominance of the stacking ensemble, paired Diebold–Mariano tests were conducted on squared prediction error sequences. The results indicate as shown in Table 15 that the stacking model significantly outperforms ANN, DT, SVM, KNN, and empirical models, with DM statistics ranging from − 3.97 to − 12.08 and corresponding p-values well below 0.05.

The negative DM statistics confirm that the stacking model consistently yields lower prediction errors than competing models. These findings demonstrate that the superior performance of the stacking ensemble is statistically significant and not attributable to random variation.

**Table 15:** Diebold–Mariano test results for statistical comparison of predictive models.

Comparison	DM statistic	*p*-value
Stacking vs. ANN	−4.43	9.43 × 10⁻⁶
Stacking vs. DT	−6.34	2.29 × 10⁻¹⁰
Stacking vs. SVM	−3.97	7.06 × 10⁻⁵
Stacking vs. KNN	−12.08	8.12 × 10⁻⁵
Stacking vs. Empirical	−10.31	7.56 × 10⁻⁵

## Conclusion

This study investigated natural soiling on solar panels subjected to different cleaning protocols and developed empirical and machine learning models for predicting soiling loss. We employ experimental analysis and data-driven methods to test, evaluate, and predict how well PV systems will work when they are dirty in the real world. Ensemble learning outperforms empirical approaches in accuracy and robustness. The experimental study on four PV panels with different cleaning frequencies (daily, weekly, biweekly, monthly) confirmed that natural soiling significantly impacts PV performance, with higher losses under longer intervals.

- This study contributes to the field by establishing a validated experimental–empirical–machine learning framework for real-world soiling prediction under controlled cleaning intervals.
- The proposed stacking model significantly outperforms the semi-empirical baseline, demonstrating improved predictive accuracy and robustness under identical validation conditions.
- Unlike purely simulation-based studies, the proposed approach is grounded in field measurements and incorporates temporal causality-aware validation, making it both scientifically rigorous and practically deployable.
- The findings provide a reproducible methodology for future PV degradation studies and open avenues for intelligent, data-driven operation and maintenance optimization in solar energy systems.
- Two empirical models were created one for $\:{I}_{SC\:}$prediction (dominated by GHI) and another for Soiling Loss (SL) (mainly impacted by RH and cleaning frequency). The SL model has R² = 0.978, but a high MAPE = 28%, suggesting insignificant nonlinear effects.
- Machine learning showed considerable increases, with the stacking ensemble obtaining the highest accuracy (R² = 0.9997, RMSE = 0.0456, MAE = 0.0333) and surpassing individual models (ANN, DT, SVM, KNN), among other models.
- Model accuracy falls according to decreased cleaning frequency, but stacking remains strong (MSE ≤ 0.003, MAE < 0.03) even with monthly cleaning.
- Statistical validation using Bland–Altman and Wilcoxon signed-rank tests confirmed stacking’s superiority, with minimal bias and narrowest limits of agreement, although minor statistically detectable differences were observed in some cases.
- Overall, the integrated experimental–empirical–ML framework demonstrates that ensemble-based data-driven models can reliably predict soiling loss, enabling optimized maintenance scheduling and predictive O&M strategies for PV systems.

While prediction error differences may appear numerically small, their operational significance becomes substantial when translated into cumulative energy losses over extended periods. As shown in Table 3, soiling losses exceeding 5% were observed under extended cleaning intervals. Accurate prediction of soiling progression enables optimized maintenance scheduling, improving energy yield and reducing operational costs. The proposed model is developed based on short-term dry-season data, during which module surface properties are assumed constant. Long-term surface degradation, coating wear, and micro-roughness evolution may influence dust adhesion behaviour and soiling accumulation rates. Such effects represent gradual structural drift and would require multi-season or multi-year datasets for comprehensive modelling. Therefore, the current framework is primarily applicable to short- and medium-term predictive maintenance planning.

The present model was developed using data collected during dry environmental conditions to ensure controlled soiling accumulation. Extreme events such as rainfall-induced natural cleaning or dust storms introduce regime shifts that require representative training data for accurate prediction. Future work will incorporate multi-season datasets including rainfall and extreme environmental conditions to enhance model robustness and generalization capability.

## Data Availability

Data is available based on request.

## List of symbols

AB: AdaBoost
AE: Autoencoder
ANN: Artificial neural network
AT: Ambient temperature
BPNN: Backpropagation neural network
CF: Cleaning frequency
CNN: Convolutional neural network
CT: Cell temperature
DHI: Diffuse horizontal irradiance
P: Atmospheric pressure
PM: Particulate matter (PM10 / PM2.5)
RH: Relative humidity
DNI: Direct normal irradiance
WS: Wind speed
GHI: Global horizontal irradiance
KNN: K-nearest neighbor
LR: Linear regression
LSTM: Long short-term memory
MAE: Mean absolute error
MAPE: Mean absolute percentage error
ML: Machine learning
MLP: Multilayer perceptron
MSE: Mean square error
RMSE: Root mean square error
RNN: Recurrent neural network
SARIMAX: Seasonal auto regressive integrated moving average with exogenous variables
SH: Sunshine hour
SL: Soiling loss
SR: Soiling ratio
SVM: Support vector machine
SVR: Support vector regression
RF: Random forest
RGB: RGB images of solar panels
DT: Decision tree
ELM: Extreme learning machine
GRU: Gated recurrent unit
XGB: Extreme gradient boosting
$\:{R}^{2}$: Coefficient of determination
$\:{I}_{mpp}$: Current at maximum power point
$\:{I}_{SC\:}$: Short-circuit current
$\:{I}_{SC\:\left(Clean\right)}$: Short-circuit current of clean reference panel
$\:{I}_{SC\:\left(soiled\right)}$: Short-circuit current of soiled panel
$\:{P}_{clean}$: Power output of clean panel
$\:{P}_{MPP}$: Maximum power output
$\:{P}_{soiled}$: Power output of soiled panel
$\:{T}_{d}$: Temperature of dusty panel
$\:{T}_{m}$: PV module temperature
$\:{V}_{mpp}$: Voltage at maximum power point
$\:{V}_{oc}$: Open-circuit voltage
